# Structural investigation of *Trypanosoma cruzi* Akt-*like* kinase as drug target against Chagas disease

**DOI:** 10.1038/s41598-024-59654-8

**Published:** 2024-05-02

**Authors:** Karina A. Stadler, Lesly J. Ortiz-Joya, Amit Singh Sahrawat, Christoph Buhlheller, Karl Gruber, Tea Pavkov-Keller, Treasa B. O’Hagan, Alba Guarné, Sergio Pulido, Marcel Marín-Villa, Klaus Zangger, Nina Gubensäk

**Affiliations:** 1 Institute of Chemistry/Organic and Bioorganic Chemistry, University of Graz, Graz, Austria; 2 Programa de Estudio y Control de Enfermedades Tropicales (PECET), Facultad de Medicina, Universidad de Antioquia, Medellín, Colombia; 3 Institute of Molecular Biosciences, University of Graz, Graz, Austria; 4 Innophore GmbH, Graz, Austria; 5 Field of Excellence BioHealth, University of Graz, Graz, Austria; 6 BioTechMed-Graz, Graz, Austria; 7 Department of Biochemistry, McGill University, Montreal, Canada; 8 LifeFactors ZF SAS, Rionegro, Colombia

**Keywords:** American trypanosomiasis, Parasitic disease, NMR, Akt/PKB, PH domain, Structural biology, NMR spectroscopy, Solution-state NMR, Computational models, Parasitology

## Abstract

According to the World Health Organization, Chagas disease (CD) is the most prevalent poverty-promoting neglected tropical disease. Alarmingly, climate change is accelerating the geographical spreading of CD causative parasite, *Trypanosoma cruzi*, which additionally increases infection rates*.* Still, CD treatment remains challenging due to a lack of safe and efficient drugs. In this work, we analyze the viability of *T. cruzi* Akt-*like* kinase (*Tc*Akt) as drug target against CD including primary structural and functional information about a parasitic Akt protein. Nuclear Magnetic Resonance derived information in combination with Molecular Dynamics simulations offer detailed insights into structural properties of the pleckstrin homology (PH) domain of *Tc*Akt and its binding to phosphatidylinositol phosphate ligands (PIP). Experimental data combined with Alpha Fold proposes a model for the mechanism of action of *Tc*Akt involving a PIP-induced disruption of the intramolecular interface between the kinase and the PH domain resulting in an open conformation enabling *Tc*Akt kinase activity. Further docking experiments reveal that *Tc*Akt is recognized by human inhibitors PIT-1 and capivasertib, and *Tc*Akt inhibition by UBMC-4 and UBMC-6 is achieved via binding to *Tc*Akt kinase domain. Our in-depth structural analysis of *Tc*Akt reveals potential sites for drug development against CD, located at activity essential regions.

## Introduction

CD is a potentially chronic and life-threatening disease caused by the protozoan parasite *Trypanosoma cruzi,* which is classified by the World Health Organization (WHO) as the most prevalent of poverty promoting neglected tropical diseases^1^. CD is endemic in Latin America and is gradually turning into a global health problem due to globalization and climate change^2–6^.

Parasite transmission from *T. cruzi* to the human host is primarily vector-borne via direct interaction with infected Triatominae bugs. Other transmission routes include oral ingestion of contaminated products, transplacental passage from mother to fetus, blood transfusions and organ transplantations from infected donors^7,8^. The clinical spectrum of CD is broad and potentially causes fatal chronic illness like cardiomyopathy, gastrointestinal or neurological diseases^1,7,8^.

The current treatment relies on two drugs developed more than five decades ago: nifurtimox and benznidazole^2,9^. These nitroheterocyclic drugs require long treatment periods, are ineffective in chronic stages of CD and are associated with a high prevalence of toxic side-effects leading to discontinued treatments^10^. Emerging resistant *T. cruzi* strains against both drugs also raise the demand for new molecular targets and treatment options against CD^11^.

Over the past few decades, many *T. cruzi* targets have been analyzed for drug development purposes^12–21^. However, until now none of those targets have led to new candidates in clinical trials yet^9,22^. In fact, the drug development pipeline for CD remains limited, even compared to other neglected diseases, like leishmaniasis^22^.

*Tc*Akt was previously proposed as a promising target for the development of drugs against CD^23,24^. Akt kinases play a central role in all organisms as they are key regulators involved in the balance between growth, proliferation and apoptosis^25,26^. *Tc*Akt inhibition causes apoptosis-like events in *T. cruzi*, confirming its essential role for the survival of the parasite^23,24,27^. Furthermore, *Tc*Akt is present in all stages of the parasite life cycle—epimastigotes, trypomastigotes and amastigotes—which is crucial for effective treatment of CD. Considerable effort has been made to inhibit *Tc*Akt based on bioinformatics and molecular docking studies^23,24^. However, the three-dimensional structure of the Akt-*like* kinase in trypanosomatids has not been determined yet, resulting in a lack of detailed information and understanding of its mechanism of action^27^.

Akt kinases, also called protein kinase B (PKB), contain an N-terminal pleckstrin homology (PH) domain, a catalytic (kinase) domain and a disordered, regulatory, C-terminal tail (C-tail)^28^. Regulation of human Akt (*Hs*Akt), comprising of the isoforms Akt1, Akt2 and Akt3, involves the interaction of the PH and the kinase domain through the so-called ‘autoinhibitory intramolecular interface’^25,26,28–30^. In this autoinhibitory state, the PH domain is packed against the kinase domain, resulting in a closed conformation of *Hs*Akt. In its autoinhibitory state, *Hs*Akt is constitutively phosphorylated at T450 and primed with adenosine triphosphate (ATP) bound in a deep cleft between the N-terminal (N-lobe) and C-terminal lobe (C-lobe) of the kinase domain^26,30^. Upon recruitment of *Hs*Akt to the plasma membrane, the PH domain binds to membrane-associated phosphatidylinositol-3,4,5-trisphosphate (PIP_3_) or phosphatidylinositol-3,4-bisphosphate (PI(3,4)P_2_), thereby inducing a disruption of the intramolecular interface^26,31^ and forming an open conformation of *Hs*Akt^30^. Dislodgement of the PH domain from the kinase domain, triggered by conformational changes in loop regions of the PH domain^28,32^, leads to subsequent phosphorylation of S473 and T308 and induces a re-orientation of the activation loop enabling high-affinity substrate binding and, in turn, full activation of *Hs*Akt^26,30^.

The active site of Akt kinases contains several structural elements that are necessary for substrate phosphorylation: the glycine-rich G-loop involved in ATP-binding, the activation loop binding the peptide substrate and the catalytic loop^33,34^. Precise interactions between these elements create an environment for phospho-transfer to substrates that contain the characteristic motif RXRXX(S/T)f, with f representing a large hydrophobic residue^35–41^. Phosphorylation of these targets on serine or threonine residues leads to activation or inhibition of the given protein^35,36^.

A crucial step in the activation represents the binding of the PH domain to membrane-associated PIPs to induce specific cellular processes^42–46^. *T. cruzi* expresses all necessary enzymes to synthesize PI(3,4,5)P_3_ as well as PI(3,4)P_2_, PI(3,5)P_2_ and PI(4,5)P_2_^44,47,48^. Each of those PIP residues is associated with distinctive molecular functions: PI(3,4,5)P_3_ is involved in actin polymerization, cell survival and cell growth, PI(3,5)P_2_ plays a crucial role in homeostasis and stimuli response, whereas PI(4,5)P_2_ is important for cell migration, gene expression and endo-/exocytosis^44^. Still, the understanding of the signaling mechanisms of PIPs and involved enzymes remains elusive and needs to be investigated further^44,47–49^.

Two putative phosphorylation sites (p-sites) could be identified in *T. cruzi* by multiple sequence alignment (MSA), T290 in the activation loop and S450 in the hydrophobic motif (h-motif)^27^. While these sites have not been experimentally confirmed in *T. cruzi*, the phosphorylation of an equivalent threonine in *Leishmania panamensis*—a closely related trypanosomatid parasite—could be experimentally detected^50^.

The phosphorylation motif RXRXX(S/T)f is a common consensus sequence recognized by protein kinases and was detected with a 1.74% abundance in the *T. cruzi* phosphoproteome at the epimastigotes, a replicative form of the parasite that colonize the digestive tract of the vector^37^. A short version of this motif, the RXXS sequence, represents a phosphorylation motif during the differentiation process from trypomastigotes to amastigotes. It could be identified in phosphoproteins associated with processes that are highly relevant to the differentiation stimulus–response such as cell communication, cellular organization, and biogenesis^51^. Analysis of the *T. cruzi* genome exhibits several homologs to identified human Akt substrates^38–40^, thus presenting putative *Tc*Akt targets: glycogen synthase kinase 3 GSK-3 (TritrypDB ID: TcCLB.507993.80), ribosomal protein S6 (ID: TcCLB.508277.120), Rab11 (ID: TcCLB.511407.60) and glyceraldehyde-3-phosphate dehydrogenase TcGAPDH (ID: TcCLB.506885.413)^41^. However, further in vitro and in vivo experiments are needed to identify and confirm putative *Tc*Akt substrates.

In this work, we present the first experimentally determined structure of an Akt-*like* protein domain of a protozoan parasite. Our NMR solution structure of *Tc*Akt-PH reveals a typical PH domain fold including a basic charged cleft. By using NMR Chemical Shift Perturbation (CSP) experiments^52,53^ we confirm the binding of inositol-1,3,4,5-tetraphosphate (Ins(1,3,4,5)P_4_), the soluble headgroup of PIP_3_, to *Tc*Akt-PH inducing local conformational changes of loop regions β1-β2, β3-β4 and β6-β7, affecting its dynamics. The presented experiments reveal that phosphorylation patterns of inositol headgroups induce distinctive conformational changes either stabilizing or destabilizing loop-to-helix transitions suggesting that dynamics of loop regions of *Tc*Akt-PH play a crucial role in regulating versatile Akt-*like* functionality. By combining experimental information with Alpha Fold (AF) calculations we present a model for the mechanism of action of *Tc*Akt involving a PIP-induced disruption of the autoinhibitory intramolecular interface between the *Tc*Akt kinase and PH domain, via bending of *Tc*Akt-PH loop β1-β2 upon ligand interaction. The molecular docking derived structure of  ATP-bound *Tc*Akt reveals strong similarities to *Hs*Akt unravelling activity essential residues. Further docking experiments show that human Akt inhibitors PIT-1^32,54^ and capivasertib^55,56^ recognize *Tc*Akt via similar binding modes compared to *Hs*Akt. Additionally, based on the AF structure of *Tc*Akt, the exact localization of the binding site of previously described *Tc*Akt inhibitors UBMC-4^23^ and UBMC-6^24^ could be determined at the kinase domain of *Tc*Akt, thus suggesting kinase-involved inhibition modes.

The presented experiments offer primary insights into the structure and function of the central protein Akt-*like* of a protozoan parasite and furthermore reveal potential drug target regions crucial for *Tc*Akt function but exhibiting significant structural differences to *Hs*Akt. The detailed understanding of the mechanism of action of *Tc*Akt forms the basis for the development of effective drugs against the expanding CD.

## Results

### N-terminal *Tc*Akt-PH forms a positively charged flexible cleft

**Figure 1:**
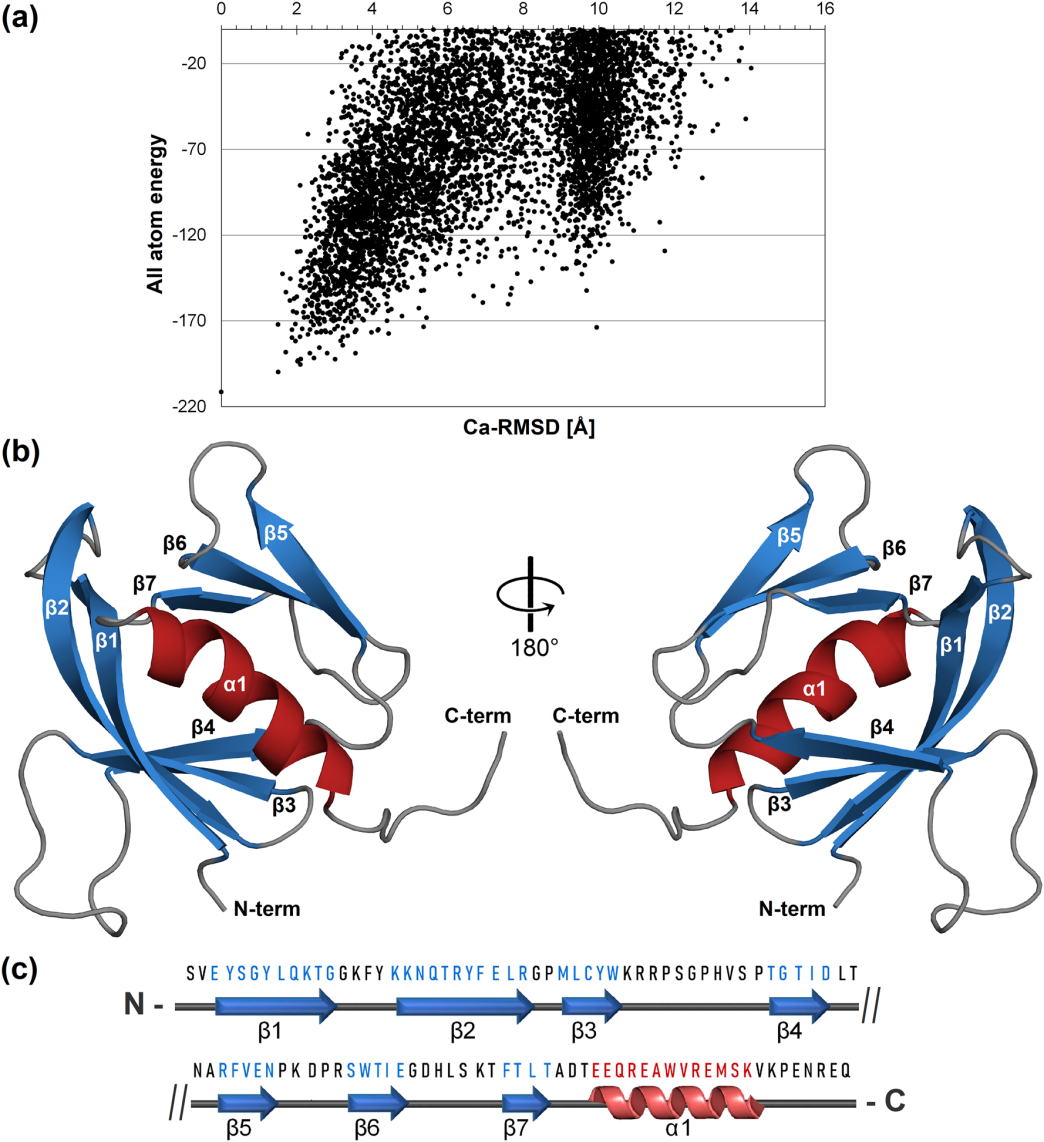
NMR derived structure of *Tc*Akt-PH (PDB: 8OZZ) (**a**) CS-Rosetta^57,58^ plot: All atom energies of *Tc*Akt-PH models with respect to their Cα-RMSD values relative to the lowest-energy model. 10 000 structures were calculated. For each structure the Cα-RMSD to the lowest-energy structure (S_07667) was calculated and plotted against the all-atom energy of each model. The run is designated as converged due to the shape of the plot and the averaged Cα-RMSD value of 1.7 Å of the final 10 structures to the lowest-energy model (S_07667). (**b**) Lowest energy structure as cartoon representation: *Tc*Akt-PH forms a typical PH domain fold, built of two antiparallel β-sheets shown in blue (β1-β4, β5-β7) and a C-terminal α-helix α1 (red). Loops are shown in grey. (**c**) Topology and amino acid sequence of *Tc*Akt-PH (aa 2–105).

The presented NMR solution structure of the membrane binding domain *Tc*Akt represents to date the only experimentally determined structure of an Akt-*like* protein domain of a protozoan parasite. The structural determination of *Tc*Akt-PH (11.7 kDa, 100 aa) was achieved by a combination of NMR experimental data including long range NOEs and CS-Rosetta^57^ (Figs. 1a, S1a, Tables S1, S2, and S3). The following structural analysis is based on the lowest energy structure of *Tc*Akt-PH (PDB: 8OZZ) (Fig. 1b).

*Tc*Akt-PH has a typical PH domain fold^46,59^ consisting of a C-terminal α-helix and two antiparallel β-sheets, formed by four and three β-strands, respectively (Fig. 1b,c). All structural elements (strands β1-β7, helix α1) contribute to the formation of the hydrophobic core (Fig. S1) resulting in a stable structure with an averaged rotational correlation time of 8.68 ns (Eq. 2), as observed by NMR relaxation experiments (Fig. S2a–d). Additionally, *Tc*Akt-PH exposes an intense network of seven surface-exposed intramolecular salt bridges, providing further stabilization of the fold (Fig. S3). Expression constructs of C-terminal truncated *Tc*Akt-PH (aa 1–95), results in a destabilization of the structure, probably due to missing helix-stabilizing residues S96 and K97.

**Figure 2:**
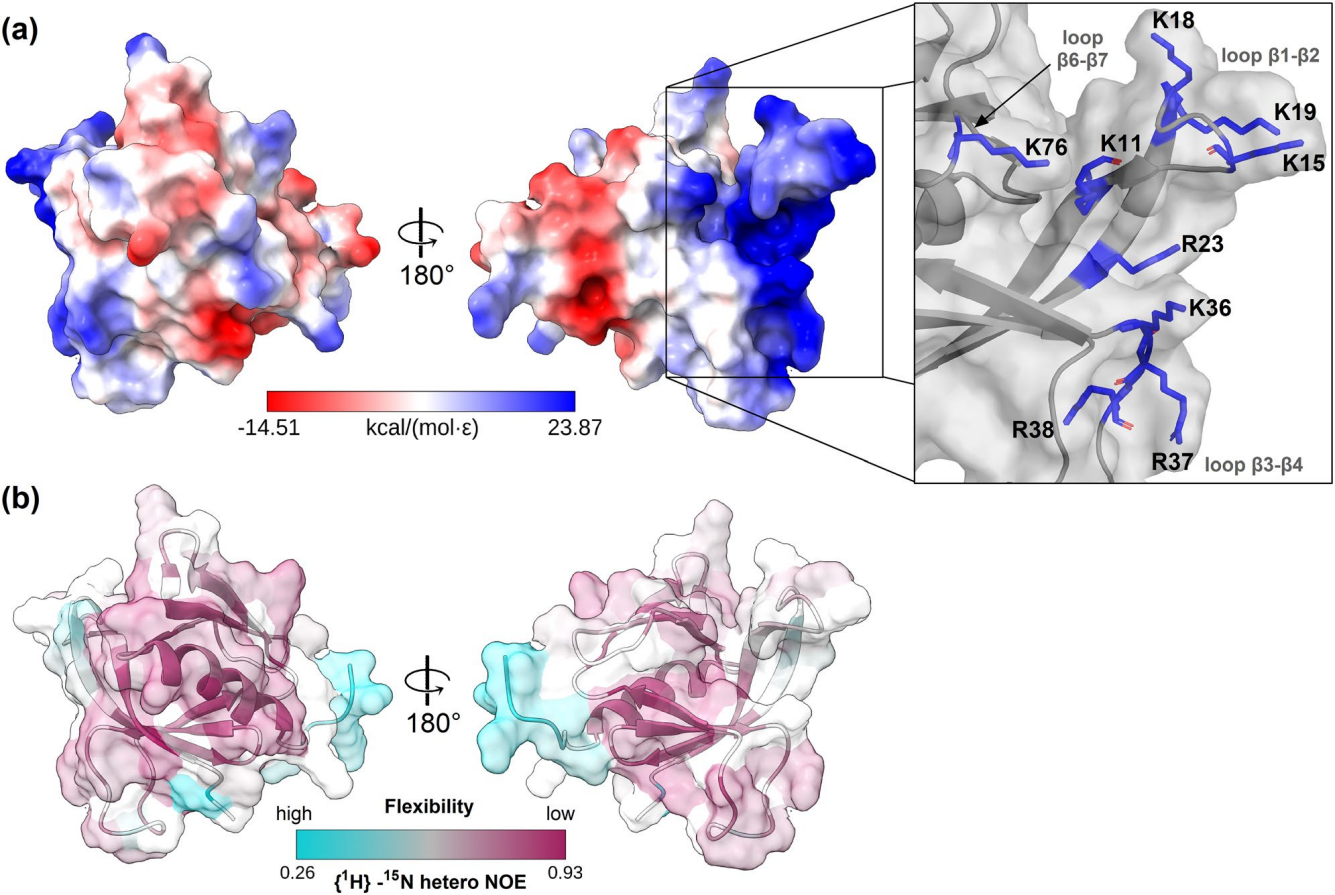
Surface representation of electrostatic potential and flexibility of *Tc*Akt-PH. (**a**) Electrostatic surface of *Tc*Akt-PH apo-form (PDB: 8OZZ) with zoomed details of nine basic amino acids: K11 (β1); R23 (β2); K15, K18, K19 (loop β1-β2); K36, R37, R38 (loop β3-β4); K76 (loop β6-β7). Coulombic electrostatic potential (kcal/(mol.ε) at 298 K) was calculated using ChimeraX^60–62^ (see Eq. 1). Negative electrostatic potential is shown in red (min: − 14.51), positive electrostatic potential in blue (max: 23.87). (**b**) Transparent surface with display of hetNOE values (see Eq. 3) of *Tc*Akt-PH apo-form: low hetNOE values are colored in cyan, high hetNOE values in purple, not assigned (n.a.) residues in white. Orientation is consistent with Fig. 1b.

Calculation of the electrostatic surface potential (see Eq. 1) in combination with NMR relaxation analysis (Figs. 2b and S2a) reveals a dynamic, positively charged cleft of *Tc*Akt-PH (Fig. 2a) gated by three flanking loops β1-β2, β3-β4 and β6-β7, which include nine basic charged residues (K11, R23, K15, K18, K19, K36, R37, R38, K76) (zoomed details in Fig. 2a).

### Phosphoinositides bind into the basic cleft of *Tc*Akt-PH

**Figure 3:**
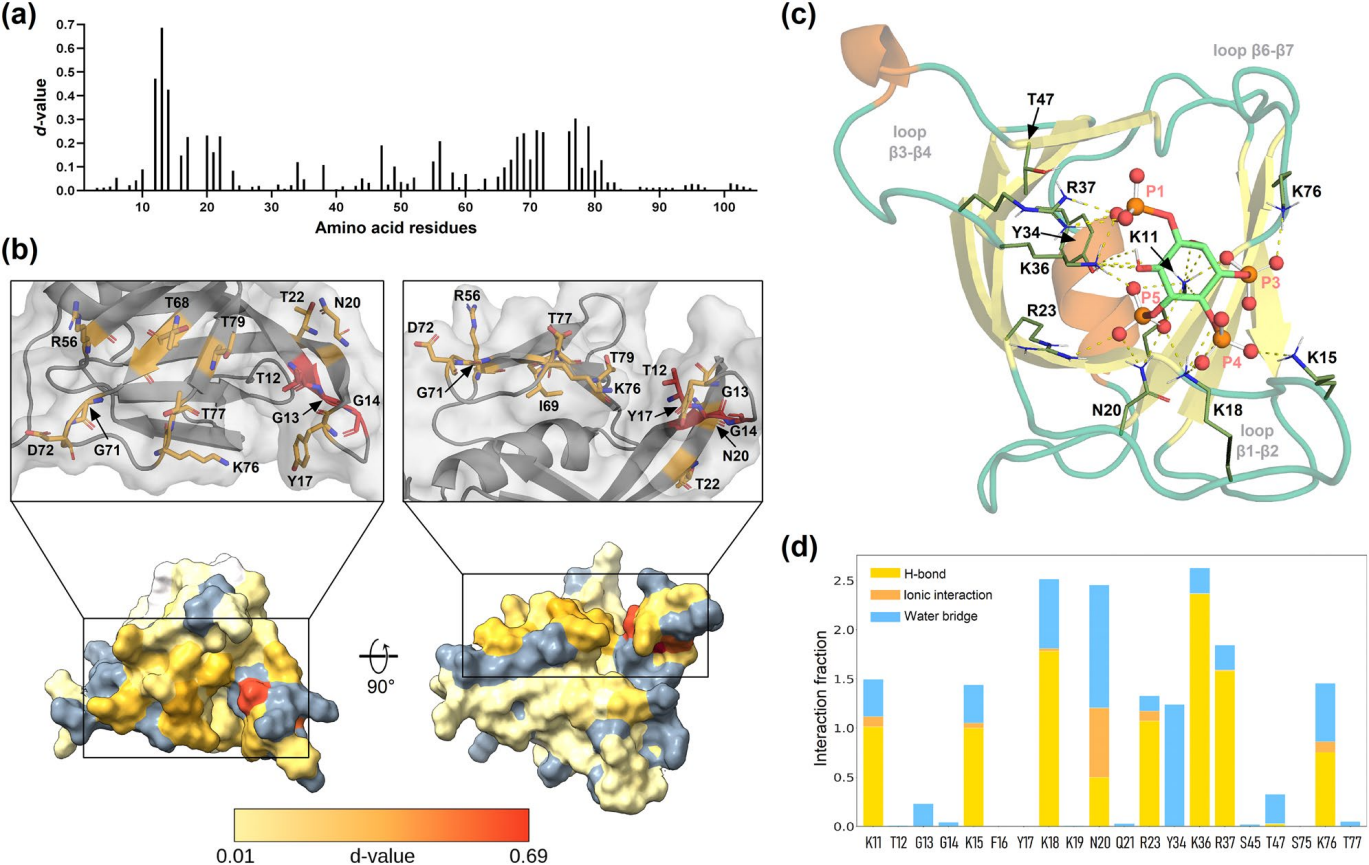
*Tc*Akt-PH PIP binding site evaluation by NMR and MD simulations. (**a**) Chemical shift perturbations of *Tc*Akt-PH. The graph includes calculated *d*-values (see Eq. 4) for each amino acid of *Tc*Akt-PH. (**b**) Surface display of *Tc*Akt-PH colored by determined *d*-values according to a gradient. Residues exhibiting high *d*-values are shown in red (max: 0.69), low *d*-values in light yellow (min: 0.01), not affected residues below threshold (< 0.008) in white, n.a. residues (due to overlap or missing peaks) are shown in grey. (**c**) MD derived structure of *Tc*Akt-PH interacting with Ins(1,3,4,5)P_4_ (a frame was carefully chosen from the last 1 µs MD simulation to showcase the interacting residues adequately): Interacting residues are shown in sticks, contacts in dashed yellow lines, phosphate groups in red (ball representation). (**d**) H-bonds, ionic interactions, and water bridges plotted as interaction fractions for each interacting residue: Bar charts are normalized over the course of the trajectory. A value of 1 indicates that the respective interaction type persists throughout 100% of the simulation time, while a value greater than 1 signifies multiple instances of the same interaction type between corresponding residue and ligand over the simulation duration (e.g., multiple hydrogen bonds between arginine and the ligand). Additional details on the computed interactions (H-bonds, ionic interactions, and water bridges) can be found in the supplementary information (SI Section 1 ‘Detailed description for computed protein–ligand interactions of *Tc*Akt-PH and Ins(1,3,4,5)P_4_ shown in Fig. 3d’).

To analyze if *Tc*Akt-PH binds to PIP ligands, we performed NMR CSP experiments using the soluble headgroup Ins(1,3,4,5)P_4_ of the proposed ligand PI(3,4,5)P_3_ (Figs. S4 and S5). The experiments confirmed an interaction of Ins(1,3,4,5)P_4_ with *Tc*Akt-PH exhibiting a dissociation constant of 40 ± 14 µM (Table S4). NMR ^1^H-^15^N HSQC spectra of *Tc*Akt-PH were recorded before and after the addition of increasing amounts of the ligand (Fig. S5). NMR chemical shifts are sensitive to their local chemical environment. Thereby, residues involved in direct interaction with a ligand can be detected via the degree of change of their chemical shift compared to the apo-form^52^. Residues directly located in the binding pocket generally exhibit a strong change of their chemical shift and/or peak intensity. Residues that do not bind to the ligand directly but experience ligand-induced conformational changes, can also be detected via CSP. For each amino acid of *Tc*Akt-PH, the Euclidean distance or *d*-value was calculated (Eq. 4) (Fig. 3a,b), defined as combined value of ^15^N- and ^1^H-shifts, thus representing the degree of change of the chemical shift of a specific residue upon ligand addition and its involvement in the ligand interaction (Fig. 3a,b).

The presented experimental data reveals that ligand-induced conformational changes affect mainly residues in or at close proximity to the positively charged cavity of *Tc*Akt-PH including loops β1‑β2, β3‑β4, and β6‑β7, thus facilitating the required opposite charge for interactions with phosphate groups of Ins(1,3,4,5)P_4_ (Figs. 2a and 3a,b). In detail, high *d*-values could be determined for strands β1, β2 and connecting β1-β2 loop involving residues: T12, G13, G14, F16, Y17, N20, Q21 and T22 (Fig. 3a). Significant chemical shift changes could be furthermore detected for residues located at strands β3, β4 and loop β3-β4 (Y34, R38, T47) as well as strands β6, β7 and loop β6-β7 (S66-T81). Residues of loop β4-β5 (A55, R56) are probably affected indirectly by the ligand interaction due to conformational changes since their location is spatially distant from the binding site. Chemical shift changes of basic residues K11, K15, K18, K19 and R23, located in strands β1, β2 and loop β1-β2, could not be analyzed in the ^1^H-^15^N HSQC spectrum due to missing signals or spectral overlap. Nevertheless, surrounding residues are highly influenced by ligand binding (Fig. 3a), thus suggesting that the mentioned basic amino acids play a crucial role in the interaction with Ins(1,3,4,5)P_4_.

Subsequent Molecular Dynamics (MD) simulations in combination with NMR experimental data reveal that the nature of the protein–ligand interactions between *Tc*Akt-PH and Ins(1,3,4,5)P_4_ is strictly polar including H-bonds, ionic interactions and water bridges (Fig. 3d, see SI Section 1, Fig. S7). Except for R38 and K19, all basic charged residues located in the positively charged cleft are interacting with the ligand (Figs. 2a and S6). Additionally, N20 has profound interactions with Ins(1,3,4,5)P_4_, as well as Y34 and T47, which form water-mediated interactions (Fig. 3c,d). Interestingly, N20 faces away from the binding site in the apo-form of *Tc*Akt but when bound to PIP_3_, N20 rotates towards the ligand, enabling interactions with PIP phosphate groups (Fig. 3b,c).

### ***Tc***Akt PH domain undergoes local conformational changes while hosting Ins(1,3,4,5)P_4_

**Figure 4:**
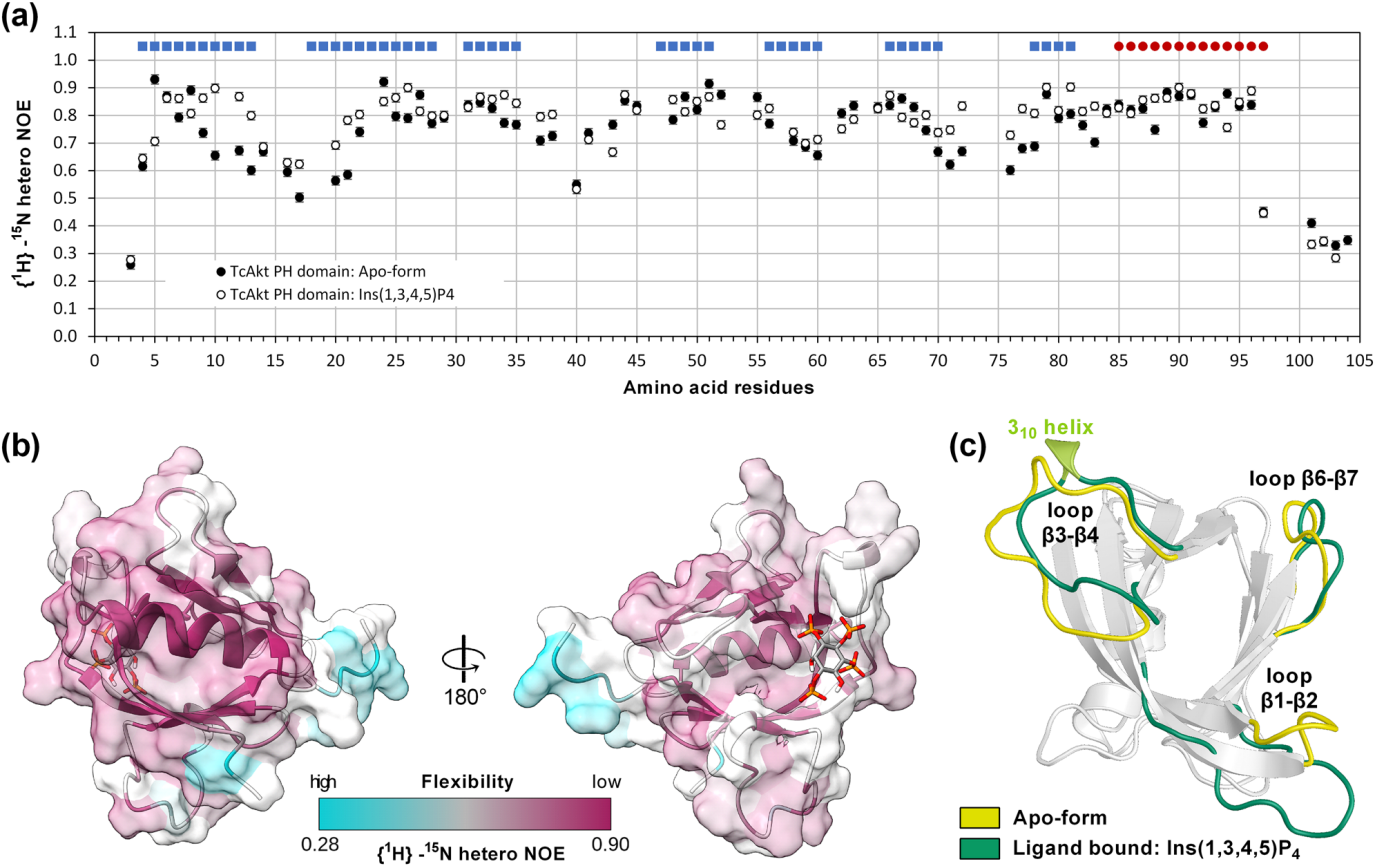
Conformational and dynamic changes of *Tc*Akt-PH initiated by Ins(1,3,4,5)P_4_ binding. (**a**) Dynamic analysis of apo *Tc*Akt-PH and Ins(1,3,4,5)P_4_ bound *Tc*Akt-PH. The hetNOE values (see Eq. 3) of residues of apo *Tc*Akt-PH are shown as black spheres. The hetNOE values of residues of *Tc*Akt-PH bound to Ins(1,3,4,5)P_4_ are shown as white spheres. *Tc*Akt-PH secondary structure elements are shown above the graph: β-strand forming residues are marked as blue squares, helix forming residues are represented as red spheres. (**b**) Structure of *Tc*Akt-PH colored according to determined hetNOE values of *Tc*Akt-PH bound to Ins(1,3,4,5)P_4_: low hetNOE values are shown in cyan (min: 0.28), high hetNOE values in purple (max: 0.90), n.a. in white. Orientation of *Tc*Akt-PH is consistent with Fig. 1b. (**c**) Structural superimposition of *Tc*Akt-PH apo-form (yellow loops) and *Tc*Akt-PH bound to Ins(1,3,4,5)P_4_ (green loops, light green 3_10_ helix).

For analyzing the impact of ligand interaction on *Tc*Akt-PH dynamics, {^1^H}-^15^N heteronuclear NOE (hetNOE) experiments were recorded before and after Ins(1,3,4,5)P_4_ addition (Fig. 4a). As shown in Fig. 4b, ligand binding induces a slight increase of rigidity of residues located at the binding site (loop regions β1-β2 and β6-β7) or close to the binding cleft of *Tc*Akt-PH (strand β3 and loop β3-β4), whereas the dynamics of the rest of the protein are not affected (Fig. 4b,c).

**Figure 5:**
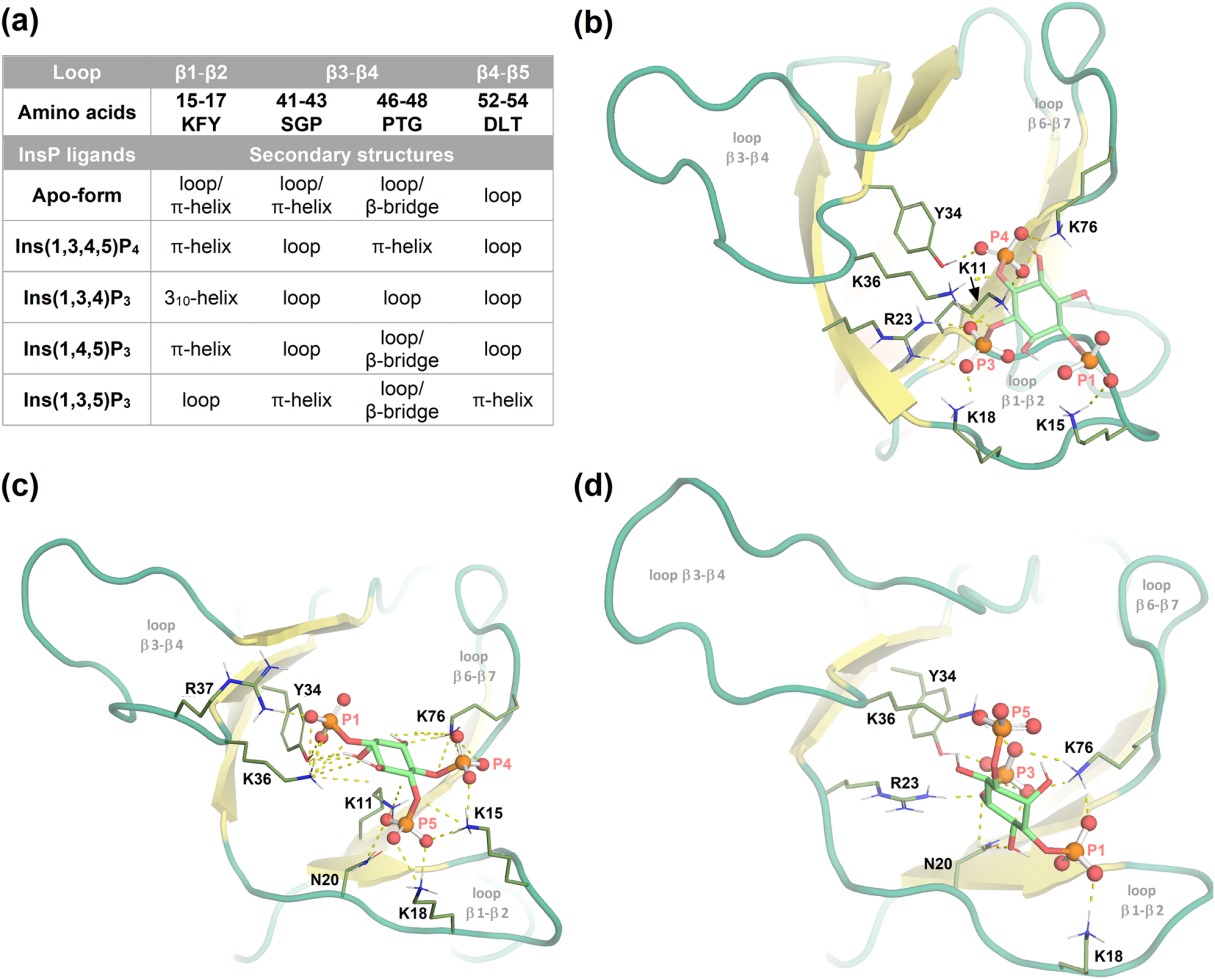
Conformational changes of *Tc*Akt-PH loop regions induced by binding of different InsP ligands. (**a**) Table of loop-to-helix transitions of *Tc*Akt-PH interacting with Ins(1,3,4,5)P_4_, Ins(1,3,4)P_3_, Ins(1,4,5)P_3_ or Ins(1,3,5)P_3_. As determined by NMR experiments, strongest binder Ins(1,3,4,5)P_4_, stabilizes loop-to-π-helix transitions in stretches 15–17 and 46–48 and simultaneously destabilizes region 41–43. Ins(1,3,5)P_3_ reduces the π-helix tendency of stretch 15–17 but induces π-helix formation in regions 41–43 and 52–54. Ligands Ins(1,3,4)P_3_ and Ins(1,4,5)P_3_ induce less conformational changes compared to Ins(1,3,4,5)P_4_ and Ins(1,3,5)P_3_, showing a significant impact mainly in region 15–17 of *Tc*Akt-PH. Ins(1,4,5)P_3_ stabilizes a π-helix in 13–15, similar to Ins(1,3,4,5)P_4_, whereas Ins(1,3,4)P_3_ represents the only ligand that induces a 3_10_-helix formation in stretch 15–17. (b-d) MD determined protein–ligand structures of *Tc*Akt-PH interacting with (**b**) Ins(1,3,4)P_3_, (**c**) Ins(1,4,5)P_3_ and (**d**) Ins(1,3,5)P_3_: Interacting residues are shown in sticks, contacts in dashed yellow lines, phosphate groups in red (ball representation). The presented frames were carefully chosen from the last 1 µs MD simulation to showcase the interacting residues adequately.

As shown by MD simulations the increase of rigidity observed by NMR can be explained by ligand-induced loop-to-helix transitions. The apo-form of *Tc*Akt-PH already reveals a tendency for loop-to-helix transitions (Figs. 5a, S8a, and S9). However, binding of Ins(1,3,4,5)P_4_ further stabilizes mentioned conformational changes for a more extended period (Figs. 5a, S8b, and S9).

Although regions 41–43 (SGP) and 46–48 (PTG) are both located in loop β3‑β4, Ins(1,3,4,5)P_4_ interaction seems to have different effects on the dynamics and conformations of the stretches. Stretch 41–34 is more solvent exposed and experiences an increase of flexibility upon ligand interaction (Figs. 4c and S9) whereas stretch 46–48 is closer to strand β4 and its helical tendency is stabilized when Ins(1,3,4,5)P_4_ is bound (Fig. S9).

Eventually, Ins(1,3,4,5)P_4_ pulls the loops β3‑β4 and β6‑β7 towards the binding site compared to their respective conformations in the apo-form (Fig. 4c). Consequently, loop β1‑β2 changes into a more open conformation upon Ins(1,3,4,5)P_4_ binding, compared to the compact, apo-form (Fig. 4c).

### Phosphorylation patterns of PIP ligands induce different conformational changes of *Tc*Akt-PH

As previously stated, PIPs act as signaling molecules involved in numerous cellular pathways depending on the position of phosphate groups on their inositol ring guiding broad Akt functionality^42–44^.

To monitor the binding behavior of different PIP ligands to *Tc*Akt-PH, MD simulations were performed using inositol phosphates (InsP): Ins(1,3,4,5)P_4_, Ins(1,3,4)P_3_, Ins(1,3,5)P_3_ and Ins(1,4,5)P_3_ (Fig. S4). According to MD data, all InsP ligands bind in the same cavity of *Tc*Akt-PH and remain bound throughout the 2 µs MD run (Figs. 3c and 5b–d), exhibiting RMSD values less than 1.5 Å (Fig. S10b) which indicates a stable interaction^63^. Ins(1,3,4,5)P_4_ shows the strongest binding due to an additional phosphate group. Similar interaction energies IE (Fig. S10a) could be calculated for other InsP ligands.

Although tested ligands bind into the same cavity of *Tc*Akt-PH, ligand-induced conformational changes of *Tc*Akt-PH loop regions are versatile and complex as shown in detail in Fig. 5a (Figs. S8 and S9). Phosphorylation patterns of PIP headgroups impact distinctive conformational changes of *Tc*Akt loops β1-β2, β3-β4 and β4-β5, resulting in ligand-specific structural rearrangements of the PH domain, which subsequently could influence the versatile functionalities of *Tc*Akt^43,44^. Still, all of the tested ligands induce a similar bending of loop β1-β2 when binding to *Tc*Akt-PH, independent on their phosphorylation pattern (Fig. S11).

### *Tc*Akt-PH prefers PIP ligands with P3 and P5 phosphorylations

In humans, a binding preference of Akt1 PH domain to PI(3,4,5)P_3_ and PI(3,4)P_2_^26,32,42,64^ was determined by monitoring its intrinsic tryptophane fluorescence upon binding to different inositol phosphates^65^, as well as by competitive HPLC binding experiments of ^32^P-labeled phosphoinositides^66^. To analyze *Tc*Akt binding preferences, we calculated the solvent-accessible surface area (SASA) and the buried surface area (BSA) of each of the tested ligands when bound to *Tc*Akt-PH (Fig. S10d). The more buried a ligand is in the binding site, the larger its BSA will be. The extent of BSA increase is an important descriptor of ligand binding^67,68^ and can therefore be used for analyzing the impact of different phosphorylation patterns of PIP ligands on the interaction with *Tc*Akt-PH.

The calculated averaged BSA (Fig. S10d) shows no significant differences between all InsPs, reflecting that the depth of the binding pocket remains consistent and is equally accessible to all InsPs irrespective of the number and relative positions of phosphate groups on the inositol ring. However, when analyzing each phosphate position individually, different BSA values could be observed (Fig. S10c). Irrespective of the type of InsP, the P1 phosphate group has the lowest BSA, meaning that it is more exposed to the solvent relative to other phosphate positions. In the membrane-bound PIP ligand, the fatty acid tail is attached on phosphate group P1 and is therefore oriented towards the membrane rather than the binding site and does not seem to form specific interactions with *Tc*Akt.

In comparison, the P3 phosphorylation has the highest BSA values in all InsPs. For Ins(1,4,5)P_3_, which lacks the P3 phosphate group, P5 seems to compensate for P3 with comparable BSA values. Thus, we propose a preference of *Tc*Akt-PH for PIP ligands containing P3 and/or P5 phosphorylations.

As previously shown, MD data reveals that all PIP ligands induce a bending of loop β1-β2 (Fig. S11). Nevertheless, different phosphorylation patterns of PIP ligands initiate different local conformational changes of *Tc*Akt and also vary in binding behavior, thus supporting a ligand specificity of *Tc*Akt. In contrast to *Hs*Akt1, which favors P3 and P4 phosphorylations of PIP^26,32,42,64^, *Tc*Akt shows a preference for P3 and P5 phosphate groups.

### The kinase domain of *Tc*Akt contains conserved motifs essential for Akt activity

**Figure 6:**
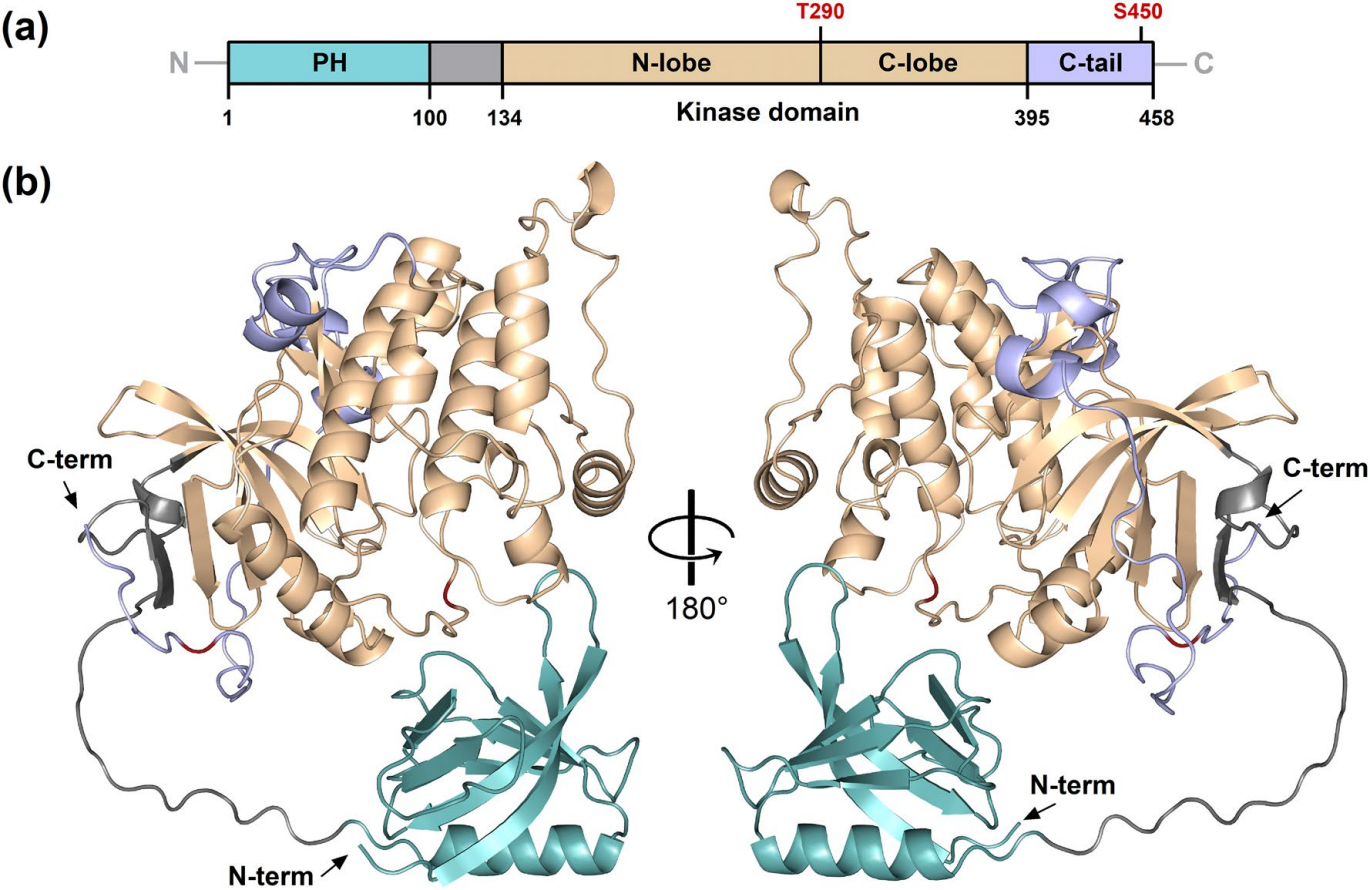
AF derived model of full-length *Tc*Akt. (**a**) Schematic representation of *Tc*Akt domains: *Tc*Akt consists of an N-terminal PH-domain (aa 1–100) (turquoise), a flexible linker (aa 100–134) (grey), a kinase domain divided in a N-lobe and C-lobe (aa 134–395) (sand) and a C-tail (aa 395–458) (lavender). Two putative p-sites are shown in red: T290, S450. (**b**) Cartoon representation of full-length *Tc*Akt model. Same color scheme as in (**a**).

Since structure determination of full-length *Tc*Akt by X-ray crystallography was not successful, we used AF^69^ for calculating the structure of *Tc*Akt (Figs. 6 and S12). According to internal quality parameters, the calculation runs were designated as successful (see Materials and Methods section ‘AF calculations’).

Crucial residues in regions associated with Akt activation are typically highly conserved among kinases and are also present in the AF *Tc*Akt structure (Figs. 6  and S14): The glycine-rich G-loop in the kinase C-lobe is crucial for ATP binding and was stated to have the consensus sequence GXGXΦG, with Φ as hydrophobic residue^33,34,56^. While this is consistent with the G-loop in *Hs*Akt1, in *Tc*Akt the third glycine within this sequence is replaced by a serine (Fig. S14), an exception that is also found in other protein kinases^70,71^. The catalytic loop, also present in the C-lobe, is responsible for the phospho-transfer from ATP to the substrate and carries a conserved aspartic acid (in *Tc*Akt D257) that interacts directly with the target S/T p-site (*Hs*: T308, *Tc*: T290) (Figs. S13 and S14)^27,34,56^. The activation loop starts with the highly conserved DFG motif and ends with the APE motif^33,34^, whereas the crucial threonine p-site (in *T. cruzi* T290) is located in between (Fig. S14). The h-motif is part of the C-tail and usually contains the sequence FXXF(S/T)(Y/F)^27^ and a p-site. *Tc*Akt shows a shortened version of the h-motif (FSF) including the putative p-site S450 (Figs. S13 and S14). Other p-sites in the C-tail that were linked to *Hs*Akt1 activation (*Hs:* S477, T479) are absent in *Tc*Akt^25,28,29^.

**Figure 7:**
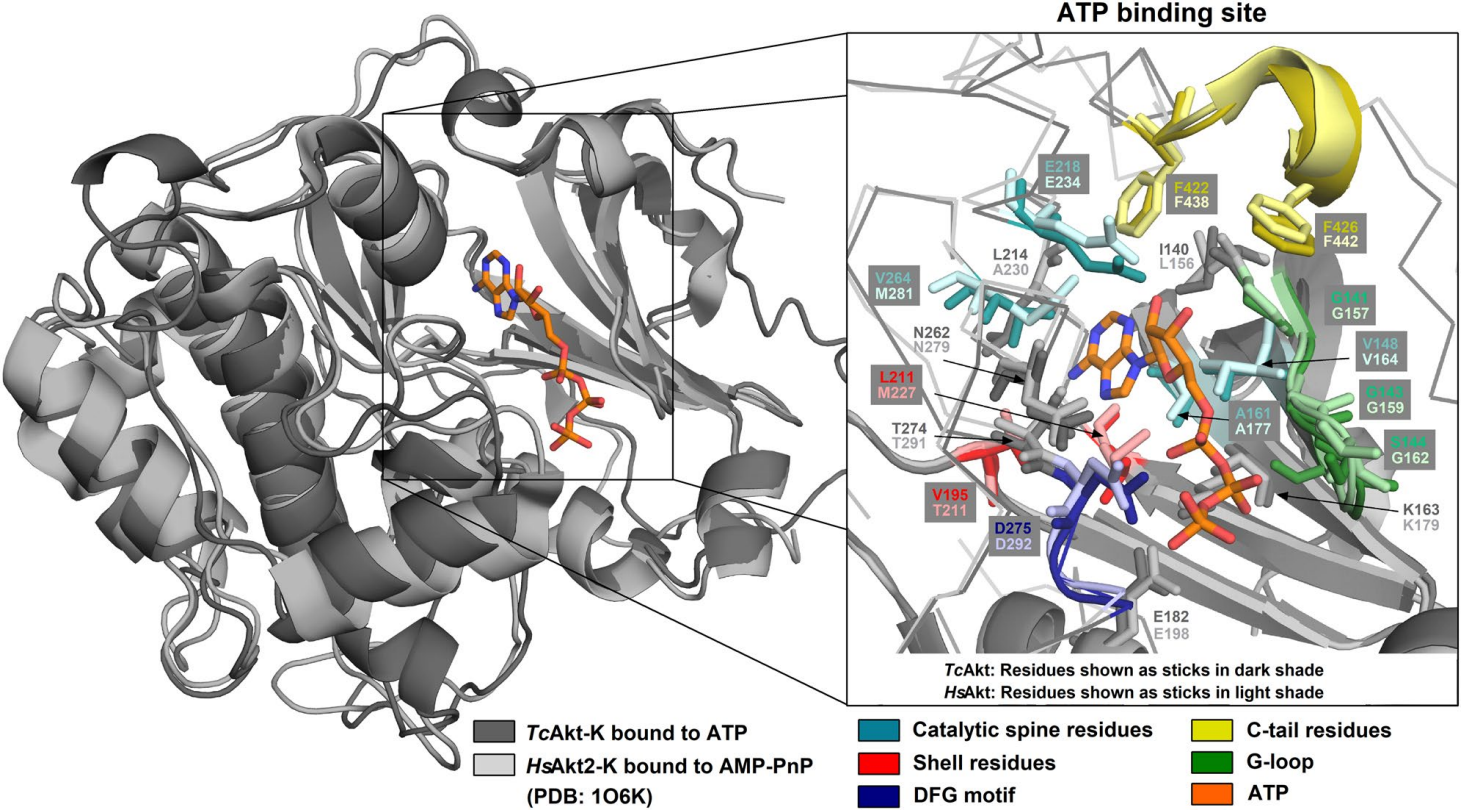
ATP bound to *Tc*Akt and *Hs*Akt2 kinase domains. *Tc*Akt kinase domain (dark grey) bound to ATP (orange sticks) was calculated with RoseTTAFold^72^. *Hs*Akt2 kinase domain (light grey) was complexed with ATP analog, AMP-PnP (not shown) (PDB: 1O6K)^73,74^ (all-atom RMSD of aligned structures 0.883 Å). Zoomed details show ATP binding site. *Tc*Akt residues are shown in dark sticks and *Hs*Akt residues are shown in light sticks. Colors refer to specific regions of the kinase domain: Catalytic spine residues (turquoise), shell residues (red), DFG motif (blue), C-tail residues (yellow), G-loop (green), other residues interacting with ATP (grey).

### Full-length *Tc*Akt retains kinase activity and possesses a similar ADP/ATP binding pocket compared to human Akt

To evaluate functional properties in a full-length context, we expressed and purified recombinant *Tc*Akt (Fig. S15a) and tested its enzymatic activity in vitro using a polyclonal antibody that recognizes its phosphorylated form. The synthetic peptide RPRAATF was used as a substrate. As shown in Fig. S15b, *Tc*Akt efficiently phosphorylates the threonine residue of the peptide substrate as did *Hs*Akt3, which was used as a positive control. This indicates that *Tc*Akt is capable of recognizing the consensus recognition motif RXRXX(S/T)f associated with Akt-mediated phosphorylation^35–37^.

Based on the presented AF structure, an ATP bound model of *Tc*Akt was calculated using the recent release of RoseTTAFold All-Atom^72^. The protein–ligand model proposes that *Tc*Akt binds ATP in a similar manner compared to *Hs*Akt2 in complex with AMP-PnP, an ATP analog, and Mn^2+^ (PDB: 1O6K) (Fig. 7), thus emphasizing a strong structural and sequential conservation in this region. Figure 7 shows the ATP binding site of *Tc*Akt kinase domain with involvement of residues from the G-loop, the C-tail, the DFG motif, catalytic spine residues and shell residues^56,75,76^. According to Kornev et al.^76^ catalytic spine residues of protein kinases, as well as the DFG motif, are essential for positioning ATP and Mn^2+^, while shell residues were identified to have a stabilizing effect^77^ (Fig. 7).

Differential scanning fluorimetry (DSF) experiments revealed an increased melting temperature (Tm) upon ADP and ATP binding only in the presence of Mn^2+^ (Figs. S16 and S17). These findings match observations reported by Pascuccelli et al.^71^, showing that *Tc*Akt requires Mn^2+^ to phosphorylate substrates in vitro, but is not dependent on Mg^2+^ (Figs. S16 and S17). The Mn^2+^ binding residues in the crystal structure of *Hs*Akt2 (N293 and D280) align with the corresponding residues in *Tc*Akt (N262 and D275), confirming a conserved binding mechanism of *Tc*Akt and *Hs*Akt (see SI Section 2).

### Interdomain interface of *Tc*Akt is established via hydrophobic and aromatic interactions

The interface between the kinase and the PH domain represents a favorable target for the inactivation of *Hs*Akt1 due to its autoinhibitory functionality^25,26,28–30^. To analyze *Tc*Akt potential as drug target against CD, a detailed examination of this region is therefore of high interest.

**Figure 8:**
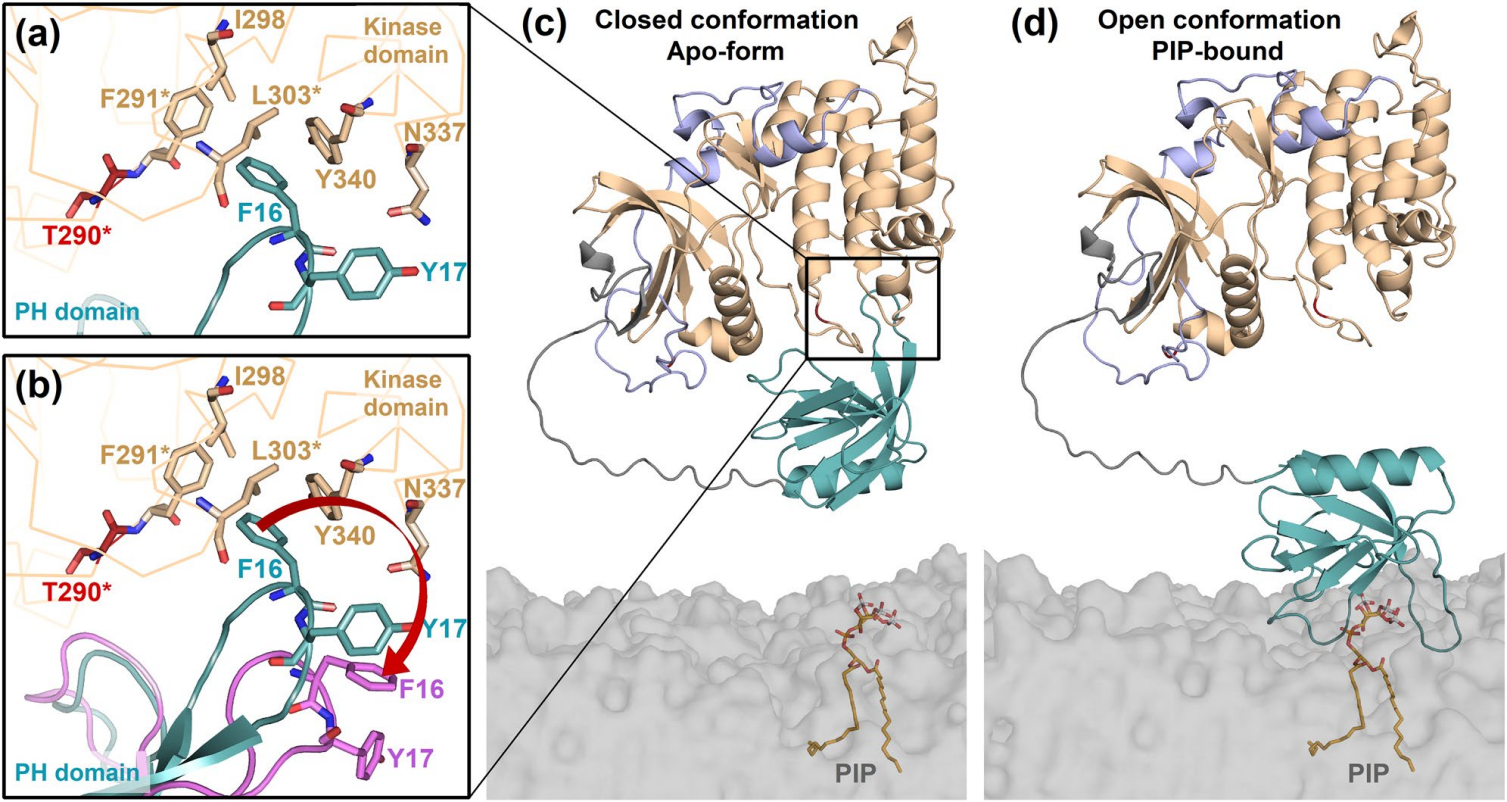
Proposed model of *Tc*Akt activation via disruption of its autoinhibitory interface upon PIP-binding. *Tc*Akt PH domain is shown in turquoise, *Tc*Akt kinase domain in sand and the C-tail in lavender. (**a**) Interdomain contacts between the hydrophobic tip of the PH domain (sticks colored in turquoise) and the kinase domain (sticks colored in sand) are shown as zoomed detail. Phosphorylation site T290 is colored in red. Conserved residues are marked with an asterisk (*). (**b**) Ligand induced bending of *Tc*Akt loop β1-β2. Superposition of *Tc*Akt apo-form (**a)** and PIP-bound *Tc*Akt  determined by MD simulations (pink). Red arrow indicates conformational changes upon binding to PIPs. (**c**) *Tc*Akt is present in a closed conformation (inactive) and gets recruited to the membrane. The PH domain and the kinase domain are interacting via an autoinhibitory interface. (**d**) The binding of the *Tc*Akt PH domain to PIP molecules in the membrane induces conformational changes of the hydrophobic tip (F16, Y17) of the PH domain loop β1-β2, leading to an opening of the structure and the disruption of the intramolecular autoinhibitory interface including a surface exposure of otherwise buried phosphorylation site T290.

**Figure 9:**
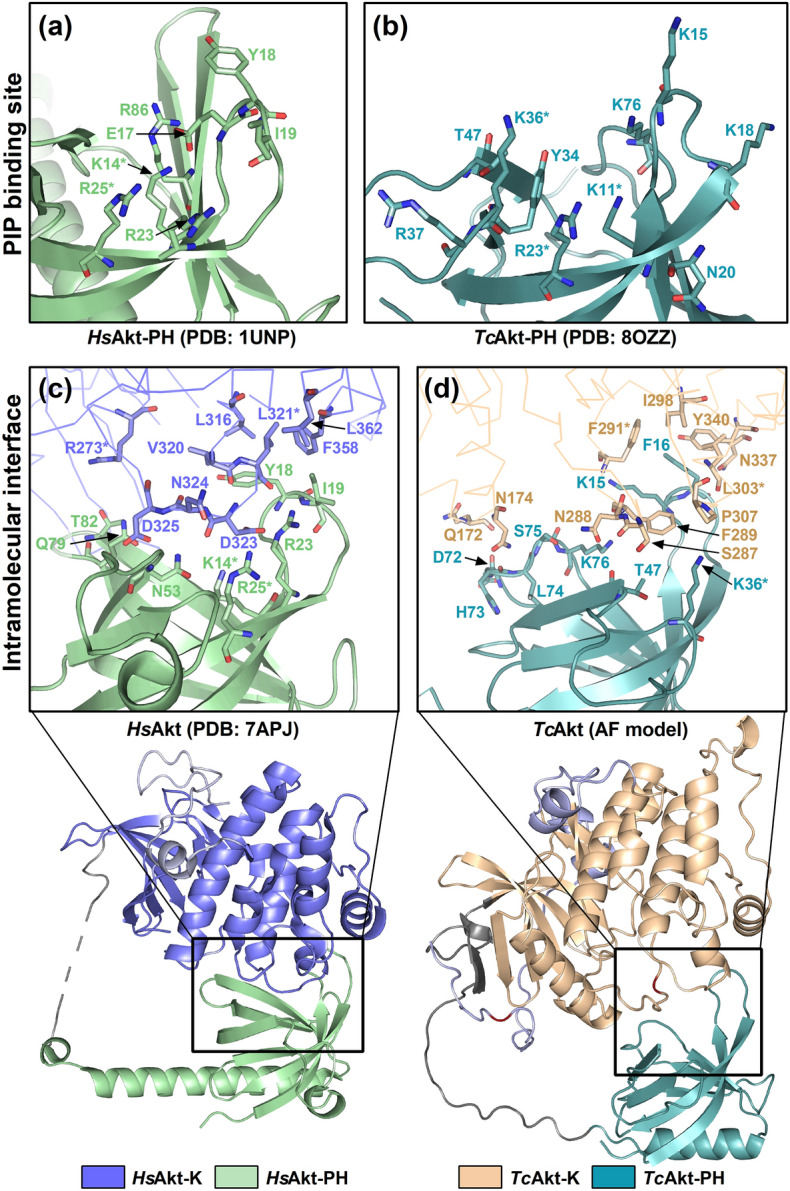
Structural analysis of activity essential regions of *Tc*Akt and *Hs*Akt1. Due to high differences in the residual arrangements the structures are not superimposed. *Hs*Akt PH domain is shown in green, *Hs*Akt kinase domain in blue and the C-tail in grey. *Tc*Akt PH domain is shown in turquoise, *Tc*Akt kinase domain in sand and the C-tail in lavender. Flexible interdomain linkers are shown in light grey. PIP binding site: (**a**) PIP binding site of *Hs*Akt1: PIP interacting residues are shown as green sticks (derived from the crystal structure of *Hs*Akt1-PH in complex with Ins(1,3,4,5)P_4_ ;PDB: 1UNQ) and presented on the apo structure (PDB: 1UNP) for comparative reasons. (**b**) PIP binding site of *Tc*Akt: PIP interacting residues are shown as turquoise sticks (derived from NMR analysis and MD simulations) and presented on the apo structure (PDB: 8OZZ). Intramolecular interface: (**c**) Intramolecular interface of *Hs*Akt1 as zoomed detail from full-length *Hs*Akt1 (PDB: 7APJ): Involved residues of the kinase (*Hs*Akt1-K: blue) and the PH domain (*Hs*Akt1-PH: green) are shown as sticks. (**d**) Intramolecular interface of *Tc*Akt as zoomed detail from full-length *Tc*Akt (AF model): Involved residues of  the kinase (*Hs*Akt1-K: sand) and the PH domain (*Hs*Akt1-PH: turquoise) are shown as sticks. Conserved residues are marked with an asterisk (*).

The AF model of full-length *Tc*Akt is present in a closed conformation forming interdomain interactions between the *Tc*Akt-PH and the kinase domain (Figs. 6 and 8c). The interface between loop β1-β2 of *Tc*Akt-PH and the C-lobe of the *Tc*Akt- kinase domain is constructed from a network of non-bonded interactions (33 contacts between 20 residues) (Figs. 9d and S18) and has a surface area of 412–460 Å^2^. The tip of loop β1-β2 of *Tc*Akt-PH carries two aromatic residues, F16 and Y17, which contribute to the hydrophobic aromatic cluster of the kinase domain built of F289, F291, F302 and Y340 (Figs. 8a, S14, and S19). F16 interacts directly with F291 and Y340, and additionally forms a weak π-stacking interaction with Y340 (ring-to-ring distance 4.2 Å) (Fig. S19). The involvement of aromatic residues in the formation of the interdomain interface between loop β1-β2 of the PH domain and the C-lobe of the kinase domain is also observed in *Hs*Akt1 (Figs. 9c and S19).

### Model of PH domain-mediated Akt autoinhibition in *T. cruzi*

In *Hs*Akt1 PIPs recruit the PH domain to the membrane by direct interactions, thus disrupting the autoinhibitory interface between the kinase and the PH domain^25,26,28–30^. This conformational rearrangement exposes the activation loop and the h-motif of the kinase domain, thereby enabling phosphorylation of T308 and S473 and activation of Akt^26,30^. In the absence of PIPs, *Hs*Akt1 stays in its inactive closed conformation, maintaining the autoinhibitory interface between the kinase and the PH domain^78^.

We propose a similar mechanism of action for *Tc*Akt. As shown in the presented AF model, hydrophobic interdomain interactions between the activation loop of the kinase domain and loop β1-β2 of the PH domain, cause a closed conformation of *Tc*Akt (Fig. 8a,c). In this conformation, the putative phosphorylation site of *Tc*Akt T290^27^ is not accessible, as it is shielded by the PH domain (Figs. 8a and S13). Binding of the *Tc*Akt-PH domain to a PIP headgroup induces a bending of loop β1-β2, as observed by MD simulations (Fig. S11). An opening of loop β1-β2 involves a change of the position of residue F16, which is thus pulled away from the interdomain interface as shown in Fig. 8b. As a consequence, the hydrophobic cluster of the kinase domain needs to be rearranged to shield the non-polar residues from the solvent exposure. This rearrangement of the region around the activation loop, including T290, could lead to an exposed position of T290, thereby enabling its phosphorylation and consequently the activity enhancement of *Tc*Akt (Fig. 8b). The described disruption of the interface is accelerated as residues K76, T47, K15 and K36, previously involved in the interface, are switching interaction partners upon PIP contact (Fig. 8b,d). The disruption of the autoinhibitory interface consequently leads to an open conformation (Fig. 8d) that is associated with activation of Akt.

### The interdomain interface of *Tc*Akt differs from *Hs*Akt

For evaluation of *Tc*Akt’s potential as drug target against CD, it is inevitable to characterize similarities and differences to its human ortholog in order to highlight putative regions for the inhibition of *Tc*Akt. The following analysis concentrates on two activity essential regions: the autoinhibitory interface and the PIP binding site, both revealing clear structural and sequential differences between *Tc*Akt and *Hs*Akt1 as shown in Fig. 9.

Overall, the presented AF model of *Tc*Akt is in good alignment with the crystal structure of *Hs*Akt1 (PDB: 7APJ) and the *Hs*Akt1 AF model (AF-DB: AF-P31749-F1), where loop regions of the kinase domain are visible^25^ (Fig. S12a,b). In contrast to the kinase domains which usually share a high conservation, the sequences of PH domains are generally more diverse among different species^45^. The sequence similarity (SS) of full-length sequences of *Tc*Akt and *Hs*Akt1 is 52.6%, while kinase domains share a 64.4% SS and PH domains have a SS of only 36.2% (Fig. S21).

As previously described, the interdomain interface of *Tc*Akt represents a promising target for Akt inhibition. As shown in Fig. 9, the structural built of the interdomain interface differs significantly between *Hs*Akt and *Tc*Akt. Despite the distinctive structural arrangements, the interdomain interface of *Tc*Akt and *Hs*Akt1 is basically constituted via a hydrophobic interface, including an aromatic cluster (Figs. 9c,d and S19). In both organisms, the hydrophobic tip of loop β1-β2 of the PH domain interacts with hydrophobic residues of the kinase domain (Fig. 9c,d). In *Tc*Akt, the hydrophobic tip of β1-β2 in *Tc*Akt is formed by residues F16 and Y17, compared to residues Y18 and I19 in *Hs*Akt1. In *Hs*Akt1, Y18 was stated to form a π-stacking interaction with F309 of the kinase domain^25,29^. In the presented AF model of *Tc*Akt, a weak π-stacking interaction is established between F16 and Y340. In *Hs*Akt1, functional relevant residues D323 and D325, as well as E17, have been described, which are involved in the interdomain interface^29^. Mentioned residues are not present in *Tc*Akt. In *Hs*Akt1 D323 and D325 form interfacial contacts to the PH domain (loop β6-β7, loop β3-β4, strands β1 and β2) and mutations result in *Hs*Akt1 hyperphosphorylation^29^. *Hs*Akt1 E17 forms a salt bridge with R86 stabilizing the autoinhibitory interface^29^. Oncogenic *Hs*Akt1 mutant E17K results in enhanced membrane binding^29^. In *Tc*Akt, the interdomain interface is not stabilized by salt bridges. *Tc*Akt contains K15, at a similar position to *Hs*Akt1 E17, but forming a hydrogen bond with F291.

### The PIP_3_ binding site of *Tc*Akt is structurally different to *Hs*Akt but is recognized by human PIP_3_ competitor PIT-1 due to its basic charge

**Figure 10:**
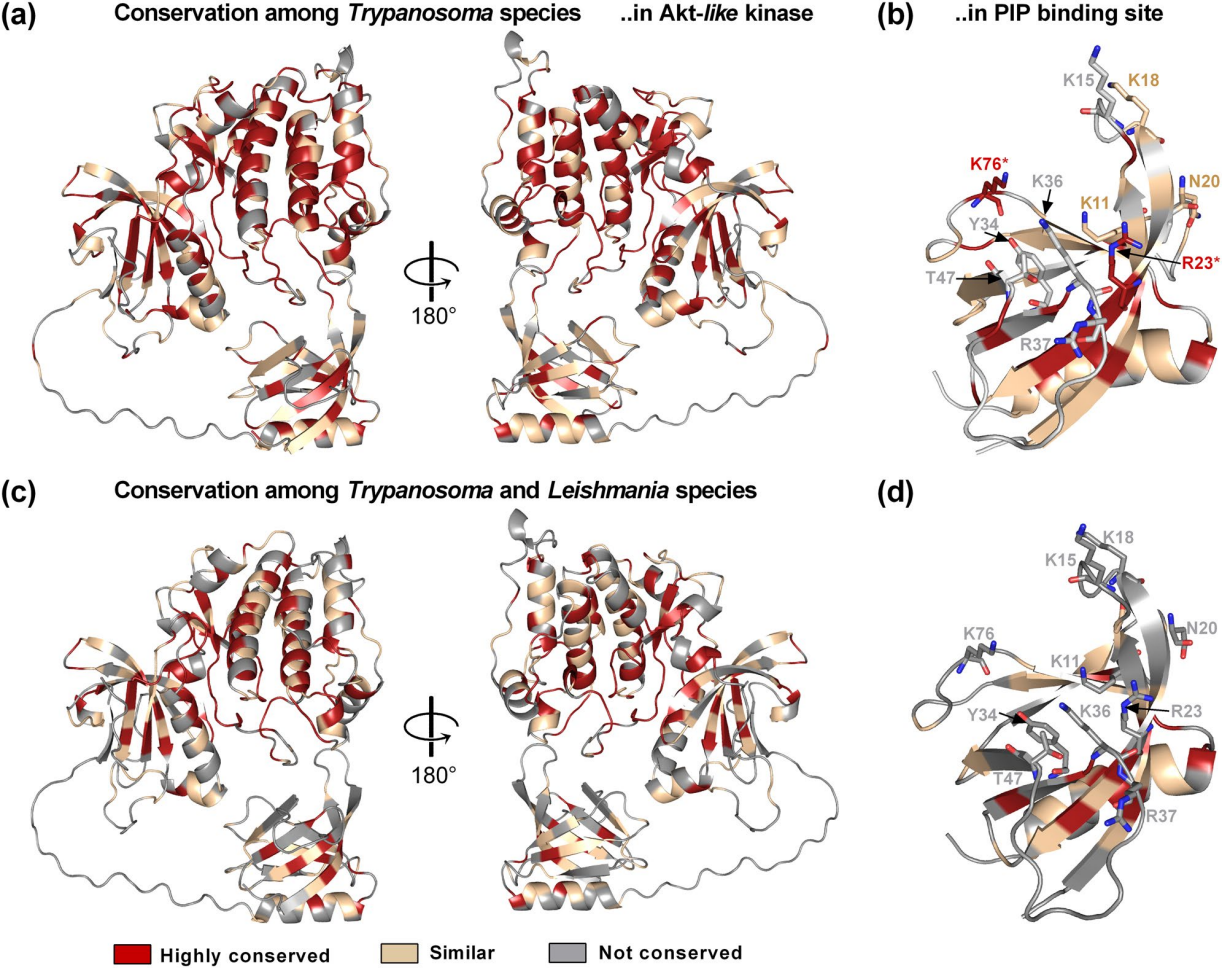
MSA derived conserved regions of Akt-*like* proteins in *Trypanosoma* and *Leishmania* species highlighted on full-length *Tc*Akt (MSA: Figs. S22 and S23). Conserved residues are shown in red (in MSA residues are marked with an asterisk (*)), residues which have similar properties are colored in sand (in MSA residues marked with dots (:,.)), non-conserved residues are shown in grey. Residues involved in PIP interaction, are shown as sticks. (**a**) Conserved regions of Akt-*like* proteins among *Trypanosoma* spp. (Fig. S22) (**b**) Conserved residues in PIP binding site among*Trypanosoma* spp. (**c**) Conserved regions of Akt-*like* proteins among *Trypanosoma* and *Leishmania* spp. (Fig. S23) (**d**) Conserved residues in PIP binding sites of *Trypanosoma* spp. and *Leishmania* spp. Further details are described in SI Section 3.

A putative region for *Tc*Akt inhibition represents the PIP binding site which regulates its activity and guides Akt function^42–44^. Despite two conserved basic residues (*Tc*: K11, R23; *Hs*: K14, R25), the binding clefts of *Hs*Akt1-PH and *Tc*Akt-PH are structurally and sequentially diverse as described in Fig. 9. The proposed consensus sequence KXn(K/R)XR located in loop β1-β2 and strand β2 to predict interactions with PIPs phosphorylated at position 3, matches with *Hs*Akt1 but not with *Tc*Akt^42,46^ (Fig. S14). Despite the differences in the amino acid composition of the binding site, the binding mode to PIP ligands is also established in distinctive manners. In mammalian PH domains mainly strands β1 and β2 (including loop β1‑β2) provide primary contact sites to PIP ligands^45,46^, involving also hydrophobic residues (loop β1-β2: Y18, I19) (PDB: 1UNQ) (Fig. 9a). In contrast, NMR and MD experiments performed with *Tc*Akt-PH reveal strictly polar protein–ligand interactions and furthermore show that besides strands β1 and β2, also loops β3‑β4 and β6‑β7 are involved in PIP interaction (Figs. 3, 4, and 5). Interestingly, according to MSA analysis, the PIP binding site also differs among closely related *Trypanosoma* and *Leishmania* species (see SI Section 3, Figs. 10, S22, and S23) proposing that PIP binding mechanisms are species-specific.

**Figure 11:**
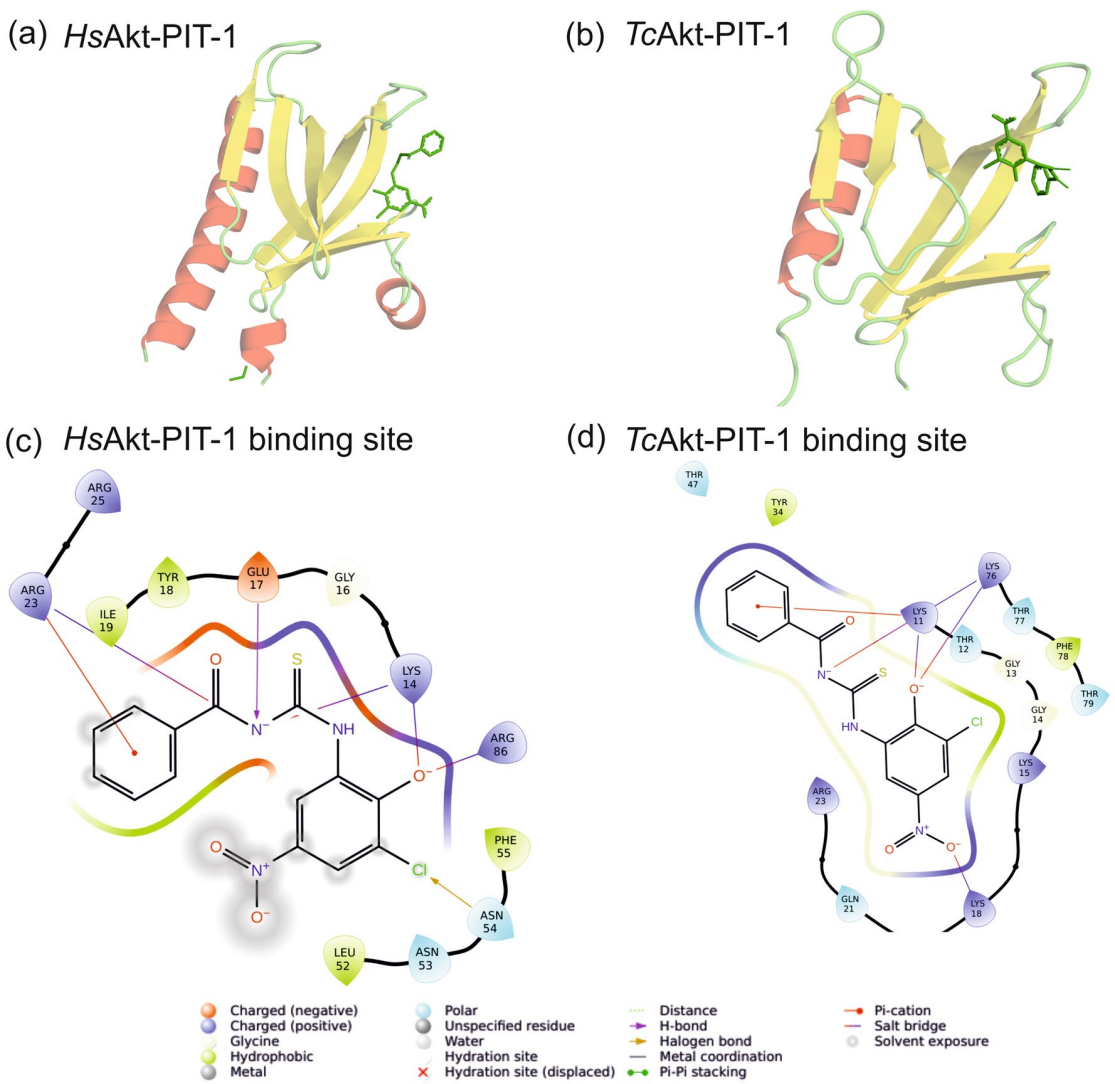
Docking studies using human PIP_3_ competitor PIT-1 with *Hs*Akt and *Tc*Akt. Docking derived model of PIT-1 bound to (**a**) *Hs*Akt-PH and (**b**) *Tc*Akt-PH. 2D representation of interacting residues within 4 Å to PIT-1 of (**c**) *Hs*Akt and (**d**) *Tc*Akt. Basic charged residues enable interaction with PIT-1. In *Hs*Akt residues K14, R23, and R86 are involved in ionic interactions with PIT-1. In *Tc*Akt PIT-1 residues K11, K18 and K76 enable PIT-1 interaction.

PIT-1 is a non-phosphoinositide small molecule antagonist of PIP_3_ that shows inhibition of Akt without affecting other PIP_2_-selective PH domains^32,54^. The PIT-1 binding site of human Akt overlaps with the PIP interaction area, involving residues W22, Y26, and N54 (N54 interacts with the phenyl group of PIT-1, W22 and Y26 interact with nitro-group of PIT-1)^54^. Using the presented AF model of *Tc*Akt, we initiated docking experiments with human PIP_3_ competitor PIT-1 revealing that the inhibitor binds to human and trypanosomal Akt in a similar manner (Fig. 11, see SI Section 4 ‘Docking studies of *Tc*Akt and human Akt inhibitors capivasertib and PIT-1’). The overall basic charge of the PIP_3_ binding pocket seems to compensate for the sequential and structural differences of the PIP_3_ interaction sites of human and trypanosomal Akt.

### General Akt inhibitor capivasertib binds to *Tc*Akt

**Figure 12:**
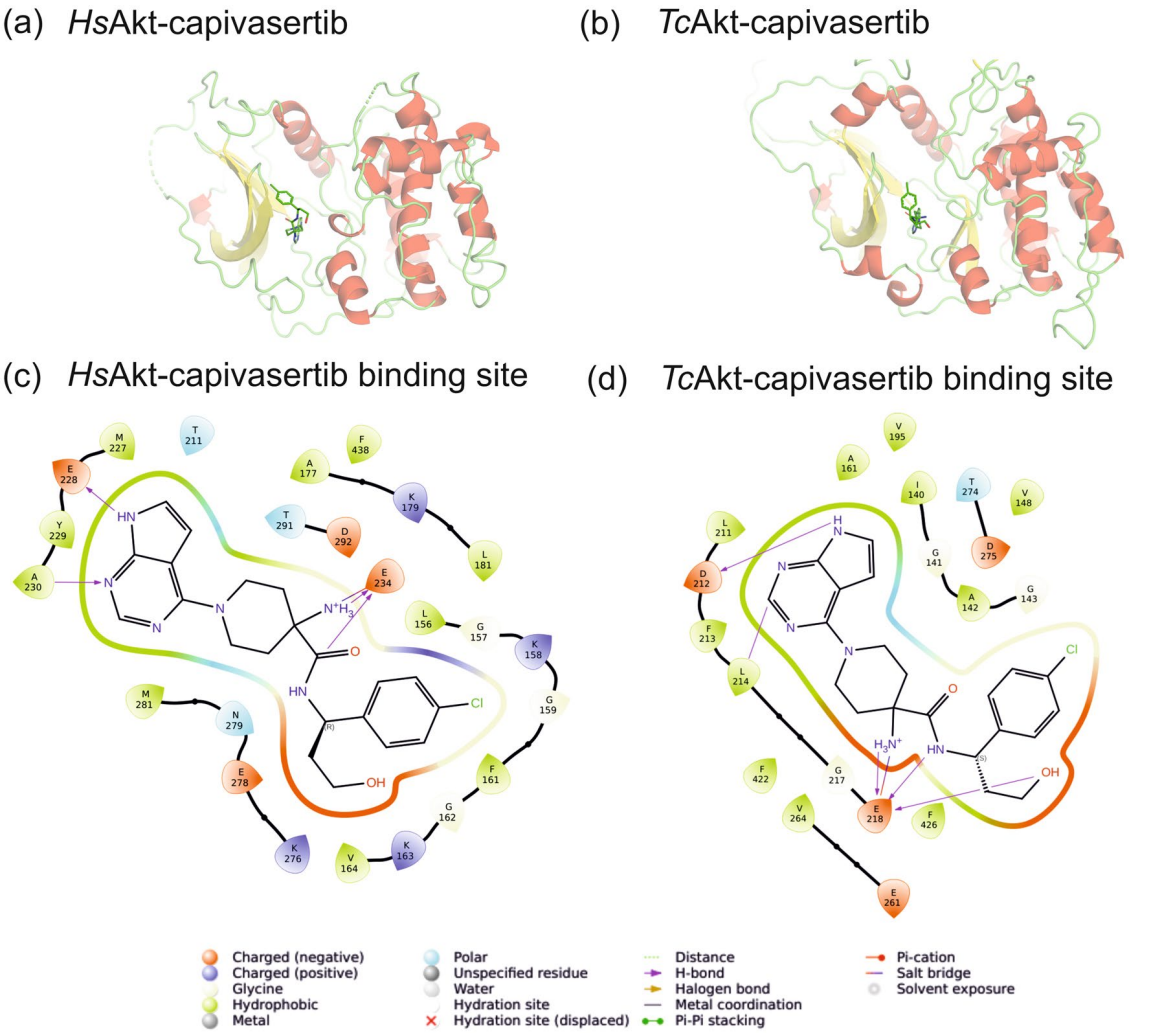
Docking studies with human Akt inhibitor capivasertib and *Hs*Akt and *Tc*Akt. Docking derived model of capivasertib bound to (**a**) *Hs*Akt-K and (**b**) *Tc*Akt-K. 2D representation of interacting residues within 4 Å of capivasertib of (**c**) *Hs*Akt-K and (**d**) *Tc*Akt-K. (see SI Section 4 ‘Docking studies of *Tc*Akt and human Akt inhibitors capivasertib and PIT-1’).

One of the best-studied examples of ATP-competitive Akt inhibitors is capivasertib^55^. Capivasertib is a pyrrolo[2,3-d]pyrimidine derivative that acts as a pan-Akt inhibitor, inhibiting all human Akt isoforms, by binding into the ATP binding site. Only recently, capivasertib was approved by the FDA for breast cancer treatment, being the first ATP-competitive Akt inhibitor on the market^55,56^. Based on the calculated AF structure of *Tc*Akt, we generated protein–ligand models of capivasertib to human and trypanosomal Akt using molecular docking approaches (Fig. 12, see SI Section 4 ‘Docking studies of *Tc*Akt and human Akt inhibitors capivasertib and PIT-1’). Although binding sites are similar between the proteins, *Tc*Akt reveals a strong stereospecifity for (S)-capivasertib over (R)-capivasertib, which is not observed to that extent for *Hs*Akt (see SI Section 4). Whereas in *Hs*Akt E234, A230 and E228 are most profound residues for interaction with (S)-capivasertib and (R)-capivasertib, in *Tc*Akt E218, L214 and D212 are responsible for the inhibitor’s binding (Fig. 12). Further experiments may be needed for evaluating the inhibitory potential of capivasertib on *Tc*Akt activity.

### *Tc*Akt inhibition by UBMC-4 and UBMC-6 involves the kinase domain

**Figure 13:**
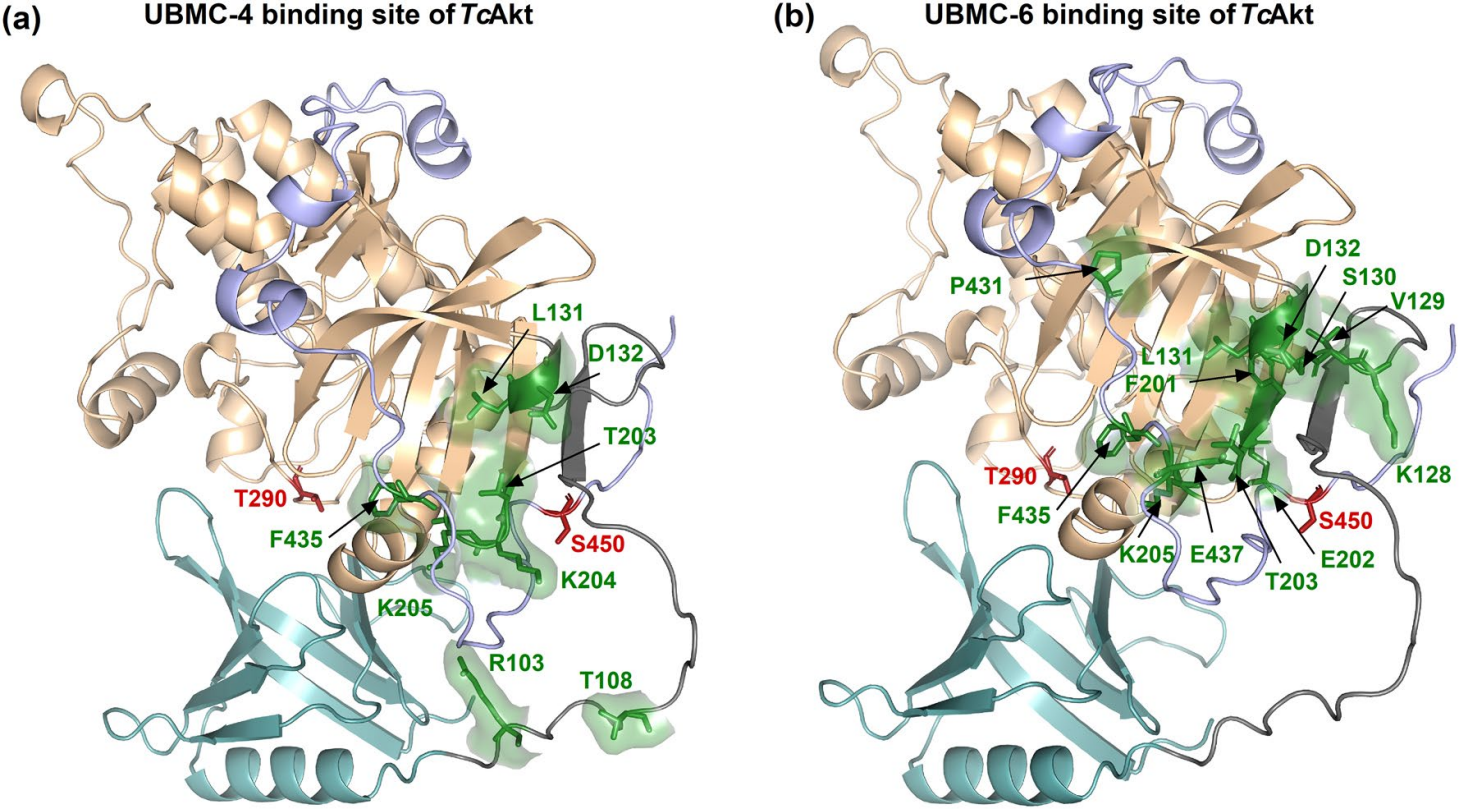
*Tc*Akt inhibitors UBMC-4 and UBMC-6 binding sites mapped on the AF model of *Tc*Akt. *Tc*Akt PH domain is shown in turquoise, the flexible linker in grey, the *Tc*Akt kinase domain in sand and the C-tail (including the h-motif) in lavender. P-sites are colored in red and shown as sticks. Ligand interacting residues of *Tc*Akt are shown in green and presented as sticks. (**a**) UBMC-4 binding site determined by Bustamante et al.^23^ (**b**) UBMC-6 binding site determined by Ochoa et al.^24^.

To date, two *Tc*Akt inhibitors UBMC-4^23^ and UBMC-6^24^ have been defined, both detected via virtual screening and molecular docking approaches. Nevertheless, the lack of structural information restricts a detailed understanding of the inhibitory mechanisms. The presented AF-model enables the determination of the binding location of previously described *Tc*Akt inhibitors, thus offering insights into their mechanism of action (Fig. 13).

UBMC-4 has been recently described as a potent *Tc*Akt inhibitor in *T. cruzi* cell structures, targeting vital cellular processes and resulting in various severe effects, including apoptosis-like events^23^. It furthermore reveals relatively low cytotoxicity on human cell lines (LC50 > 40 μM) and effective absorption in mice models^23^. *Tc*Akt inhibitor UBMC-4 was assumed to bind to the PH domain according to published MD studies^23^. Nevertheless, combining published data with the presented AF model, localizes the UBMC-4 binding region at the linker region between the PH and the kinase domain (R103, T108) and at the kinase domain (L131, D132, T203, K204, F435) (Fig. 13). The binding position of UBMC-4 suggests that inhibition of *Tc*Akt is achieved by restricting the flexibility of the interdomain linker region und thereby preventing PIP induced conformational changes as well as an opening of *Tc*Akt.

UBMC-6 represents another putative *Tc*Akt inhibitor, having an inhibitory effect on *T. cruzi* amastigotes and was also tested for its toxicity on human monocyte-derived macrophages^24,79^. According to the presented AF structure, UBMC-6 binds close to the h-motif of *Tc*Akt and the p-sites T290 and S450 (K128, V129, S130, L131, D132, F201, T203, K205, P431, F435, E437), an important region to regulate Akt stability^80^. Likely, UBMC-6 binding blocks the activity essential h-motif and may lead to Akt protein degradation.

## Discussion

The presented NMR structure, in combination with interaction experiments, MD simulations, Molecular Docking and AF-calculations, provides primary insights into the structure and function of a putative target against CD: *Tc*Akt. The experiments reveal a PIP binding area, including ligand-induced versatile conformational changes of *Tc*Akt regions, which likely guide the broad functionality of this central protein. The experimentally derived information combined with a full-length model of *Tc*Akt and sequential analysis exposes activity essential regions of the proteins, leading to a proposed model of *Tc*Akt activation via PIP-induced disruption of its interdomain interface. Detailed structural information reveil activity crucial regions of *Tc*Akt with clear structural differences to the human ortholog *Hs*Akt1 and thus highlights putative regions for targeting *Tc*Akt inhibition.

### Conformational changes induced by PIP ligands depend on phosphorylation patterns and play a crucial role in Akt activity

*Tc*Akt binds PIP headgroups differing in phosphorylation of the inositol ring in the same binding pocket with comparable binding affinities but with clear preferences for P3 and P5 phosphorylations (Fig. S10). Phosporylation patterns of PIP headgroup inositol rings furthermore induce distinctive patterns of conformational changes, mainly regarding loop regions β1-β2, β3-β4 and β4-β5 of *Tc*Akt-PH (Figs. 5a, S8, and S9). Nevertheless, all tested PIP headgroups result in an opening of loop β1-β2 upon binding to the PH domain, potentially inducing the disruption of the autoinhibitory interface (Fig. 8b).

In *Hs*Akt1, activation is strictly limited to lipid second messenger molecules PI(3,4,5)P_3_ and PI(3,4)P_2_, revealing a different ligand specificity compared to *Tc*Akt^26,32,42,64,65,81,82^. In *Hs*Akt1, Ins(1,3,4,5)P_4_ induces a loop-to-helix transition of residues 44–46 (DVD) located in loop β3-β4^32,81,83^. Destabilization of the helical formation by mutagenesis (DVD to GPG) consequently resulted in poor kinase activity of S473-phosphorylated Akt, suggesting that dynamics of the DVD motif are essential for the displacement of the PH domain from the kinase domain in response to pS473^28^.

Similarly, Ins(1,3,4,5)P_4_ also stabilizes a loop-to-helix transition of residues 41–43 (SGP) in loop β3-β4 of *Tc*Akt (Figs. 5a, S8, and S9). Other tested ligands did not induce these conformational changes. Three of the four tested ligands result in helical conformations of region 15–17 (KFY) located at loop β1‑β2 at the direct binding site of PIP, whereas only Ins(1,3,5)P_3_ destabilizes the helix transition. Destabilizing effects of Ins(1,3,5)P_3_ lead to increased flexibility, which could enable interactions with other molecules or regulate functionality^84^. Nevertheless, further experiments are needed to determine the exact effect of local conformational changes on the activity of *Tc*Akt.

The presented experiments show a complex pattern of diverse stabilizing or destabilizing effects of PIP ligands on *Tc*Akt, proposing, similarly to *Hs*Akt1, that the dynamics of loop regions of the PH domain are crucial for the functionality of *Tc*Akt. Targeting the PIP-affected loop regions via binding of a ligand could offer another possibility for *Tc*Akt inactivation by preventing subsequent structural changes essential for activity.

### Potential strategies for *Tc*Akt inhibition

*Hs*Akt has undergone extensive investigation as a therapeutic target in oncology^85^ and more recently it has been studied as drug target for treatment of cardiovascular diseases^86^, metabolic syndrome^54^, Parkinson’s disease^87^ and schistosomiasis, caused by the parasite *Schistosoma mansoni*^88^. To inhibit Akt kinases, several inhibitors have been developed based on various approaches, which potentially guide the way for *Tc*Akt inhibition.

ATP-competitive inhibitors target the active site in the open conformation of Akt kinases^89–92^. However, a high similarity of the ATP-binding site in *Tc*Akt, *Hs*Akt1 and potentially other AGC family kinases needs to be considered (Fig. 7)^93,94^. Presented docking studies could confirm that ATP-competitive inhibitor capivasertib recognizes human and trypanosomal Akt via similar binding areas (Fig. 12, SI Section 4).

Another target region for Akt inhibition represents the PIP-binding site of the PH domain. Small molecule antagonists of PIP_3_ showed activity against PIP_3_-dependent PI3K/Akt signaling^54^ supporting that this represents a promising region for Akt inhibition. Although the structure and sequence of the PIP binding region seems to be species-specific (Figs. 5, 9, 10, and 11), the general basic charge of this area may compensate for structural differences as shown by docking experiments with human PIP_3_ competitor PIT-1 and human Akt and *Tc*Akt (Fig. 11, SI Section 4).

Allosteric inhibitors bind to the inactive closed conformation of Akt by stabilizing the PH-kinase domain interface thus preventing Akt recruitment to the membrane and consequent Akt activation^89,95^. By targeting both domains, allosteric inhibitors reveal a high selectivity also among Akt isoforms^96–98^. Exploiting the presented significant differences between *Hs*Akt1 and *Tc*Akt interdomain interfaces (Fig. 9), this class holds potential for the development of *Tc*Akt-specific inhibitors.

## Conclusion

In 2019 the WHO established a ‘World Chagas Disease Day’ on 14^th^ of April in order to raise awareness for this neglected tropical disease and its global spread due to factors like climate change. The current lack of safe and efficient treatment in combination with the rising drug resistance of causative protozoan parasite *T. cruzi*, emphasizes the need for new strategies in order to fight this potentially chronic disease. This work offers a detailed analysis of a central protein of *T. cruzi* (Akt-*like*) as potential drug target against CD and includes atomic resolution data of activity essential regions, thereby forming the basis for structure-based rational drug design.

## Materials and methods

### Chemicals

All reagents were purchased in analytical grade. Ammonium chloride (^15^N, 99%) 98% (CAS 39466-62-1) was purchased from Eurisotop. D-Glucose (U-^13^C_6_, 99%) 98% (CAS 110187-42-3) was purchased from Cambridge Isotope Laboratories, Inc. Inositol 1,3,4,5-tetrakisphosphate > 95% (CAS 210488-61-2) was purchased from Echelon Biosciences Inc.

### Constructs

The gene encoding *Tc*Akt (TriTrypDB: TcCLB.509047.110, NCBI GeneID: Tc00.1047053509047.110) (1374 bp) originates from the *T. cruzi* CL Brener strain. The sequence is coding for full-length *Tc*Akt (52.1 kDa). The *Tc*Akt (aa 1–458) and *Tc*Akt-PH (aa 1–105) constructs were codon-optimized for *E. coli*, synthesized and inserted into the pET-28a(+) standard vector with a C-terminal 6xHis-tag (see SI Cloning).

### Protein expression and purification

All experiments were performed with *E. coli* BL21(DE3) (Novagen^®^). Plasmids were transformed into BL21 cells by electroporation (1.70 kV, 600 Ohm, 10 µF). Single colonies were selected from agar plates, cultured overnight at 37 °C in 10 mL LB medium containing kanamycin (50 mg/mL) and stored in cryo stocks at − 80 °C.

For isotopic labeling, 1000 mL M9 minimal medium (Tables S7  and S8) containing 0.15% ^15^N-NH_4_Cl, 0.3% ^13^C-Glucose and 50 mg/mL kanamycin were inoculated with the overnight-culture (ONC) and incubated at 37 °C, 180 rpm until the OD_600_ reached 0.6–0.8. Induction of protein expression was performed by adding 0.5 mM isopropyl 1-thio-ß-D-galactopyranoside (IPTG) and cells were incubated overnight at 25 °C in a baffled shaking flask (180 rpm).

Cells containing *Tc*Akt-PH protein were pelleted at 6000 x*g*, resuspended in 20 mL lysis buffer (50 mM KPi buffer pH 8.0, 10 mM imidazole, 300 mM NaCl) with protease inhibitor (Mix HP, Serva) and disrupted by sonication (45% amplitude, 2 s on, 2 s off) for 15 min on ice. Cell lysates were centrifuged at 4 °C for 1 h at 20,000 x*g* and the supernatant was loaded onto a pre-equilibrated Ni^2+^-NTA gravity column (Cube Biotech GmbH). The column was washed with 5 column volumes (CV) wash buffer W1 (50 mM KPi pH 8.0, 10 mM imidazole, 1 M NaCl), W2 (50 mM KPi pH 8.0, 20 mM imidazole, 300 mM NaCl) and W3 (50 mM KPi pH 8.0, 35 mM imidazole, 300 mM NaCl), respectively. The elution of His-bound protein was done with 10–12 mL elution buffer (50 mM KPi pH 8.0, 350 mM imidazole, 300 mM NaCl). The sample was loaded onto a pre-equilibrated size exclusion chromatography (SEC) column (HiLoad 26/600 Superdex 75, GE Healthcare) and the protein was isocratically eluted with SEC buffer (50 mM KPi pH 7.0, 150 mM NaCl, 0.02% NaN_3_) into 4 mL fractions. Fractions containing the pure protein were pooled, dialyzed against NMR buffer (50 mM KPi pH 6.5, 100 mM NaCl, 0.02% NaN_3_) in 3.5 kDa by dialysis tubes (Spectra/Por, Repligen) and concentrated with ultrafiltration devices (Amicon Ultra, MWCO 3000, Millipore).

Cells containing full-length *Tc*Akt protein were pelleted at 6000 x*g*, resuspended in 20 mL lysis buffer (50 mM Tris–HCl pH 8.0, 500 mM NaCl) supplemented with protease inhibitor cocktail containing 100 mM PMSF (BioBasic), 100 mM Benzamidine, 0.5 mg/ml Leupeptin, and 70 mg/ml Pepstatin A (BioShop), and sonicated on ice for 4 min (18% amplitude, 60 s on, 60 s off). The mixture was centrifuged at 26,800 × *g* at 4 °C for 1 h, and the soluble protein sample was subjected to Ni^2+^ affinity chromatography (HisTrap, GE Healthcare Life Sciences). The column was washed with 5%, 7%, and 12% (v/v) imidazole of the elution buffer (50 mM Tris–HCl pH 8.0, 500 mM NaCl, 250 mM imidazole) 5 CV each. The protein was obtained with 100% elution buffer in 1.2 mL fractions. Then, the sample was diluted with 50 mM Tris–HCl pH 8.0 buffer to a final concentration of 125 mM NaCl, filtered using a 0.2 µm filter, and subjected to anion exchange chromatography on a Mono Q™ 10/100 GL column (Cytiva). The column was pre-equilibrated with buffer (50 mM Tris–HCl pH 8.0, 125 mM NaCl) and the protein was eluted with 500 mM NaCl using a stepwise gradient (125–500 mM) with a flow rate of 1.0 mL/min for 60 min. Pure protein fractions were collected, buffer exchanged (50 mM Tris–HCl pH 8.0, 125 mM NaCl), and concentrated using ultrafiltration devices (Amicon Ultra, MWCO 3000, Millipore). Finally, the concentrated protein fraction was loaded onto the SEC column, pre-equilibrated with buffer (50 mM Tris–HCl pH 8.0, 150 mM NaCl), and protein elution was performed utilizing the same buffer at a flow rate of 0.2 mL/min.

Protein concentration was determined by measuring the absorbance at 280 nm with the specific extinction coefficients for full-length *Tc*Akt (ɛ = 55,700 M^−1^ cm^−1^) and *Tc*Akt-PH (ɛ = 23,900 M^−1^ cm^−1^). The purity of the proteins was confirmed by SDS-PAGE, followed by Coomassie blue staining.

### Akt activity assay

The kinase activity of purified recombinant *Tc*Akt-6His was measured using a solid phase enzyme-linked immuno-absorbent assay kit (Abcam, ab139436), according to the manufacturer’s instructions. Briefly, 200 ng purified protein was incubated with ATP (1 µg/µl) for 60 min at 30 °C. The phosphorylation of the synthetic peptide was detected with a phospho-specific substrate antibody incubated 60 min at 21 °C. Subsequently, multiple washes were performed, and anti-rabbit IgG:HRP conjugate (1 µg/mL) and TMB substrate were added. The colorimetric detection was measured at 450 nm in a spectrophotometer (Varioskan Flash Multimode Reader, Thermo Scientific). The human *Hs*Akt3 was used as a positive control and kinase assay dilution buffer was used as blank. Each reaction was performed in triplicate, and the results were expressed as relative kinase activity. Data were analyzed using GraphPad Prism 8.0.1.

### DSF experiments

The Tm of *Tc*Akt-6His was determined by monitoring the fluorescence intensity of SYPRO Orange dye (Thermo Fisher) bound to protein as a function of temperature. The protein was diluted to 1.6 µM in a buffer containing 50 mM Tris–HCl (pH 8.0) and 150 mM NaCl in the presence or absence of the indicated divalent cations (2 mM MgCl_2_ and 2 mM MnCl_2_ ∙ 4H_2_O) with Sypro 5 × at a final volume of 25 μl into the wells of a 96-well thin wall PCR plate. The nucleotides were evaluated at 16 μM. Thermal scanning (20–95 °C at 1.0 °C/min) was carried out in the real-time PCR equipment (CFX Connect, Biorad) measuring the intensity of fluorescence every 10 s with the SYBR channel. The melting temperature and the first derivative curve were calculated using the software of the equipment. Data are shown as mean from three independent experiments. Reactions without protein, in the presence of reaction buffer, and SYPRO, were included as controls.

### NMR experiments

All spectra were recorded in 3 mm NMR tubes at 25 °C with a 700 MHz Bruker Avance III NMR spectrometer, equipped with a cryogenically cooled 5 mm TCI probe. ^13^C-NOESY^99,100^, ^13^C-HSQC^101^ and HCCH-TOCSY^102–104^ were recorded from a 500 µM ^15^N- and ^13^C-labeled sample in 100% D_2_O, all other experiments for backbone and side chain assignment were recorded with a 500 µM ^15^N- and ^13^C-labeled sample in 90% NMR buffer (50 mM KPi pH 6.5, 100 mM NaCl, 0.02% NaN_3_) and 10% (v/v) D_2_O. Spectra were processed with NMRPipe (NMRDraw v5.6 Rev)^105^ and analyzed with CcpNmr Analysis 2.4.2.^106^. Molecular images were created with PyMOL (v2.0 Schrödinger, LLC) and UCSF ChimeraX (RBVI)^60^.

### Backbone and side chain assignment

Sequential backbone assignments were determined from the following 3D experiments: ^15^N-HSQC^107^, HNCO^108,109^, HN(CA)CO^109,110^, HNCA^108,109,111^, HN(CO)CA^108,109^, HNCACB^112,113^, HN(CO)CACB^114^, CC(CO)NH^115,116^. For side chain resonance assignments (^13^C and ^1^H) we used ^13^C-HSQC, H(CCO)NH^115,116^, HCCH-TOCSY^102–104^ and CC(CO)NH spectra.

### Interproton distance restraints

Interproton distance restraints (NOEs) were obtained from a 3D ^15^N-NOESY^99,100,117^ (80 ms mixing time) and a 3D ^13^C-NOESY (130 ms mixing time) experiment. NOE assignment was achieved by a combination of CYANA-automated NOE assignment^118,119^ and manual assignment. Secondary structure predictions were done using backbone assignments and TALOS+^58^ (Fig. S24).

### CS-Rosetta structure calculation

For the CS-Rosetta fragment generation we used the provided CS-Rosetta server as described by Shen et al.^57^ using the following input parameters: backbone chemical shifts (Cα, Cβ, C’, Hα and HN), TALOS+ restraints^58^ and NOEs between the following protons: HN-HN, Hα-HN and Hα-Hα (82 NOEs, including 26 long-range NOEs: *i *− *i* + (10–100)) (Table S1). Using TALOS+ predictions, the flexible termini were excluded from the subsequent calculations of *Tc*Akt-PH (aa 2–105) (Fig. S24). For the structure generation we used an installed version of Rosetta 3.13^57,58^ and additionally included side chain NOEs (59 NOEs including 50 long-range NOEs:* i *− *i* + (10–100)) (Table S2). 10 000 structures were calculated. The output was validated by plotting the Cα-RMSD of each structure to the lowest-energy structure (S_07667), against the all-atom energy of each structure (Fig. 1a). The plot reveals a clear funnel towards the lowest-energy model, thereby indicating that the CS-Rosetta structure calculation is converged. Ten structures were selected according to the lowest Cα-RMSD to the lowest-energy structure resulting in an averaged Cα-RMSD value of 1.7 Å, additionally confirming successful structure calculation. Refinement statistics were determined via the PSVS server (PMID: 17186527) (Table S3). The best-scored model (S_07667) was used for further structural analysis.

### Calculation of electrostatic surface

The coulombic electrostatic potential was calculated from atomic partial charges and coordinates according to Coulomb’s law:

1 $$\mathrm{\varphi }=\sum \left[{{\text{q}}}_{{\text{i}}}/\upvarepsilon {{\text{d}}}_{{\text{i}}}\right]$$

φ… potential, q…atomic partial charges, d…distances from the atoms, ε…dielectric constant.

The resulting potential is in units of kcal/(mol·e) at 298 K. Standard amino acids are assigned atomic partial charges and types from the recommended force field versions in AmberTools 20 (for proteins: ff14SB)^61,62^.

### NMR relaxation experiments

^15^N T1 spin–lattice relaxation times were determined from an inversion recovery experiment (Bruker pulse sequence: hsqct1etf3gpsi3d.2) with delay times of 0.05, 0.1, 0.2, 0.3, 0.4, 0.5, 0.6, 0.7, 0.8, 1.0, 1.5, 2.0, 3.0 and 3.5 s. ^15^N T2 spin–spin relaxation times were determined from a spin echo CPMG experiment (Bruker pulse sequence: hsqct2etf3gpsi3d) with delay times of 0.017, 0.034, 0.051, 0.068, 0.085, 0.102, 0.119, 0.136, 0.153, 0.170, 0.187, 0.204, 0.237 and 0.330 s. Spectra were analyzed using CcpNmr Analysis 2.4.2.^106^. The rotational correlation times τ_C_ were calculated for each residue from Eq. (2) (Fig. S2b) and the mean value was calculated:

2 $${\uptau }_{{\text{C}}} \approx \frac{1}{4\uppi {\upnu }_{{\text{N}}}} \sqrt{6\frac{{{\text{T}}}_{1}}{{{\text{T}}}_{2}}-7}$$

${\uptau }_{{\text{C}}}$…rotational correlation time [s], ${\upnu }_{{\text{N}}}$…^15^N resonance frequency [Hz], ${{\text{T}}}_{1}$…T1 relaxation time [s], ${{\text{T}}}_{2}$…T2 relaxation time [s].

HetNOE values were measured with the pulse sequence hsqcnoef3gpsi. Spectra were analyzed using CcpNmr Analysis 2.4.2.^106^. HetNOE values were determined from the measured intensities of a saturated ^15^N-HSQC spectrum relative to a reference spectrum:

3 $${{\text{NOE}}}_{{\text{het}}}=\left(\frac{{{\text{I}}}_{{\text{sat}}}}{{{\text{I}}}_{{\text{ref}}}}\right)-1$$

${{\text{NOE}}}_{{\text{het}}}$…{^1^H}-^15^N heteronuclear NOE, ${{\text{I}}}_{{\text{sat}}}$…Intensities of saturated spectrum, ${{\text{I}}}_{{\text{ref}}}$…Intensities of reference spectrum.

### NMR CSP experiments

For CSP experiments, a 170 µM ^15^N-labeled sample and a 30 mM stock of Ins(1,3,4,5)P_4_ in NMR buffer were prepared. Small volumes of Ins(1,3,4,5)P_4_ were added stepwise to the protein sample up to a ratio of 1:35 (protein:ligand). ^15^N-HSQC spectra were recorded after each step to follow the shift changes. Spectra were analyzed using CcpNmr Analysis 2.4.2.^106^.

Euclidean distances, also called *d*-values, were calculated as described by Williamson^53^:

4 $$d=\sqrt{\frac{1}{2}\left[{{\delta }_{H}^{2}+(\alpha *{\delta }_{N})}^{2}\right]}$$

$d$…Euclidean distance, ${\delta }_{N}$…^15^N chemical shift changes, ${\delta }_{H}$…^1^H chemical shift changes, $\alpha$…scaling factor (glycines $\alpha$=0.2, all other amino acids $\alpha$=0.14).

A threshold value was determined according to the procedure described by Schumann et al.^120^ to exclude residues with very small shift changes.

The ^15^N chemical shifts were weighted with a scaling factor α = 0.14. The dissociation constant (K_d_) was then fitted for each amino acid individually with the following equation:

5 $$\Delta {\updelta }_{{\text{obs}}}=\Delta {\updelta }_{{\text{max}}}\left\{\left({[{\text{P}}]}_{{\text{t}}}+{[{\text{L}}]}_{{\text{t}}}+{{\text{K}}}_{{\text{d}}}\right)-\sqrt{{\left({\left[{\text{P}}\right]}_{{\text{t}}}+{\left[{\text{L}}\right]}_{{\text{t}}}+{{\text{K}}}_{{\text{d}}}\right)}^{2}-4{\left[{\text{P}}\right]}_{{\text{t}}}{\left[{\text{L}}\right]}_{{\text{t}}}}\right\}/2{\left[{\text{P}}\right]}_{{\text{t}}}$$

$\Delta {\updelta }_{{\text{obs}}}$…change in observed shift, $\Delta {\updelta }_{{\text{max}}}$…maximum shift change on saturation, ${[{\text{P}}]}_{{\text{t}}}$…total protein concentration, ${[{\text{L}}]}_{{\text{t}}}$…total ligand concentration, ${{\text{K}}}_{{\text{d}}}$…dissociation constant.

K_d_ values with *d*-values below the calculated threshold (*d*-value < 0.008) were excluded from the dataset. Outliers were identified via boxplot analysis and excluded from the dataset (K_d_ > 90 µM). Mean and standard deviation were calculated from the resulting 64 values (Table S4).

### MD simulations

To model the *Tc*Akt-PH structure with ligands (Ins(1,3,4,5)P_4_, Ins(1,4,5)P_3_, Ins(1,3,4)P_3_, Ins(1,3,5)P_3_), we have used the experimental structure of the PH domain of *Hs*Akt (PDB: 1UNQ) with bound Ins(1,3,4,5)P_4_ as a template. The coordinates of the *Tc*Akt-PH structure were aligned with the *Hs*Akt-PH structure. Subsequently, using the builder tool in Maestro (Schrödinger, LLC)^121^, we have utilized the coordinates of the bound ligand to manipulate the structure of the original ligand and model other ligands with the aligned structure of *Tc*Akt-PH. Subsequently, hydrogen atoms were added using the protein preparation wizard^122^ in Maestro. Each modeled *Tc*Akt-PH structure without ligands and with ligands then undergoes an energy minimization step only for the H atom, followed by solvation and neutralization.

For equilibration, the system was subjected to 100 ps of Brownian Dynamics NVT at 10 K with restraints on solute-heavy atoms, followed by short 12 ps NVT and 12 ps NPT at 10 K with restraints on solute-heavy atoms. Later, the temperature was increased to 300 K for another 12 ps NPT run with restraints on solute-heavy atoms. Finally, all restraints were removed, and a short 24 ps NPT run was performed, followed by another 1 µs long NPT at 300 K.

The equilibrated system was then further simulated for 1 µs long production runs at 300 K. In total, each system was simulated for 2 µs. For these simulations, the program Desmond^123^ was used with the OPLS4^124^ all-atom forcefield. A time step of 2 fs was used throughout the simulations, employing a Nose–Hoover^125,126^ thermostat and a Martyna-Tobias-Klein^127^ barostat, with relaxation times of 1.0 and 2.0 ps, respectively. The particle mesh Ewald^128^ method was used to treat long-range interactions, and a nonbonded cutoff of 9.0 Å was used for short-range interactions. For analysis, the last 1 µs production run has been used.

### AF calculations

*Ab-initio* models for *Tc*Akt were calculated using an AlphaFold 2.3 installation in standard configuration for monomers with full databases and monomer model weights^69^. With the full-length sequence (aa 1–458) as input, 25 models were generated and ranked by the highest pTM score. The top-ranked model (see Fig. 6) reached a pTM score of 80.6.

### RoseTTAFold All-Atom assembly calculation

The models of the *Tc*Akt kinase domain bound to ATP were calculated on a RoseTTAFold All-Atom^72^ installation in standard configuration. A set of 5 models was generated with the domain sequence (331 aa) and the chemical structure of ATP as input. All 5 predicted models show ATP at the same binding site. The models were ranked by the lowest pae_inter score, the top-ranked model reached a score of 7.6. The models were relaxed using amber99sb^129^ and GAFF^130^ force fields with parameters oriented to the relaxation algorithm of AlphaFold-Multimer^69,131^. The highest ranked model is shown in Fig. 7.

### Molecular docking

The structure of *Hs*Akt was retrieved from the RCSB PDB under accession code 4GV1^55^. Non-protein atoms were then removed from the structure, leaving only the bound inhibitor, Capivasertib. Subsequently, protein preparation was conducted using Maestro^121^ which involved the addition of hydrogen atoms following an energy minimization process to refine the positions of the added hydrogen atoms. The coordinates of a modeled *Tc*Akt structure were aligned with those of the 4GV1 structure, utilizing the bound inhibitor’s coordinates for grid generation. A receptor grid was generated around the bound inhibitor to facilitate docking. The inhibitor molecule underwent a separate ligand preparation step to explore its possible conformations and stereoisomers. Generated ligand conformations were then subjected to docking into the receptor using the extra precision protocol of Glide software^132^. Furthermore, a similar procedure was repeated for docking the PIT-1 (CAS 53501-41-0) inhibitor with the PH-domain of *Hs*Akt, employing both the PDB:1UNQ structure and the PH domain of the AF structure of *Tc*Akt.

### Supplementary Information

Supplementary Information.

## Data Availability

The NMR solution structure of *Tc*Akt-PH has been deposited in the PDB with ID 8OZZ. NMR chemical shift assignments have been deposited in the BMRB data bank with accession number 52088. Most data generated and/or analyzed during the current study are included in this article and its supplementary information files. Corresponding raw data tables are available upon request to the corresponding authors.
