# Supplementary material for: Structural investigation of Trypanosoma cruzi Akt-like kinase as drug target against Chagas disease
Source: Sci Rep. 2024 May 2;14:10039. doi: 10.1038/s41598-024-59654-8 (PMC11063076; doi:10.1038/s41598-024-59654-8)
Supplement: Supplementary file 1 — Supplementary Information. [file 41598_2024_59654_MOESM1_ESM.docx]

Supplementary Information

Structural investigation of *Trypanosoma cruzi* Akt-*like* kinase as drug target against Chagas disease

Karina A. Stadler ^1,+^, Lesly J. Ortiz-Joya ^1,2,7,+^, Amit Singh Sahrawat ^3,4^, Christoph Buhlheller ^3^, Karl Gruber ^3,4,5,6^, Tea Pavkov-Keller ^3,5,6^, Treasa B. O'Hagan ^7^, Alba Guarné ^7^, Sergio Pulido ^2,8^, Marcel Marín-Villa ^2^, Klaus Zangger ^1,5,6,^*, Nina Gubensäk ^3,^*

^1^ Institute of Chemistry/Organic and Bioorganic Chemistry, University of Graz, Graz, Austria

^2^ Programa de Estudio y Control de Enfermedades Tropicales (PECET), Facultad de Medicina, Universidad de Antioquía, Medellín, Colombia

^3^ Institute of Molecular Biosciences, University of Graz, Graz, Austria

^4^ Innophore GmbH, Graz, Austria

^5^ Field of Excellence BioHealth, University of Graz, Graz, Austria

^6^ BioTechMed-Graz, Graz, Austria

^7^ Department of Biochemistry, McGill University, Montreal, Canada

^8^ LifeFactors ZF SAS, Rionegro, Colombia

*Corresponding authors: nina.gubensaek@uni-graz.at, klaus.zangger@uni-graz.at

Table S1: List of backbone NOEs including HN-HN, Hα-HN and Hα-Hα (82 NOEs including 26 long-range NOEs: *i* - *i* + (10-100))

| **Atom 1** | **Residue 1** | **Atom 2** | **Residue 2** |  | **Atom 1** | **Residue 1** | **Atom 2** | **Residue 2** |
| --- | --- | --- | --- | --- | --- | --- | --- | --- |
| **3 very long-range NOEs** *(i - i + 25-100)* | | | |  | **42 sequential NOEs** (*i - i + 1*) | | | |
| HA | 28 | HA | 3 |  | H | 85 | H | 86 |
| H | 10 | H | 81 |  | H | 59 | H | 60 |
| H | 29 | H | 4 |  | H | 83 | H | 84 |
| **23 long-range NOEs** (*i - i + 10-24*) | | | |  | H | 62 | H | 63 |
| H | 35 | HA | 25 |  | H | 4 | H | 5 |
| H | 80 | H | 69 |  | H | 68 | H | 69 |
| HA | 69 | HA | 57 |  | H | 87 | H | 88 |
| H | 82 | H | 67 |  | H | 88 | H | 89 |
| HA | 81 | HA | 66 |  | H | 93 | H | 94 |
| H | 50 | H | 34 |  | H | 95 | H | 96 |
| HA | 49 | HA | 33 |  | H | 90 | H | 91 |
| H | 25 | HA | 8 |  | H | 89 | H | 90 |
| H | 50 | H | 32 |  | H | 104 | H | 105 |
| HA | 26 | HA | 6 |  | H | 101 | H | 102 |
| H | 27 | HA | 6 |  | H | 71 | H | 72 |
| H | 29 | H | 4 |  | H | 57 | H | 58 |
| H | 5 | H | 27 |  | H | 105 | H | 106 |
| H | 7 | H | 25 |  | H | 48 | H | 49 |
| H | 32 | H | 50 |  | H | 47 | H | 48 |
| H | 34 | H | 48 |  | H | 40 | H | 41 |
| H | 34 | H | 48 |  | H | 77 | H | 78 |
| H | 56 | H | 70 |  | H | 86 | H | 87 |
| H | 67 | H | 80 |  | H | 34 | H | 35 |
| H | 67 | H | 80 |  | H | 5 | H | 6 |
| H | 24 | H | 35 |  | H | 43 | H | 44 |
| H | 58 | H | 69 |  | H | 94 | H | 95 |
| H | 58 | H | 68 |  | H | 26 | H | 27 |
| **7 middle range** (*i - i + 4-9*) | | | |  | H | 78 | H | 79 |
| H | 69 | H | 78 |  | H | 96 | H | 97 |
| HA | 25 | HA | 34 |  | H | 81 | H | 82 |
| H | 26 | H | 34 |  | H | 51 | H | 52 |
| H | 60 | HA | 67 |  | H | 101 | H | 102 |
| H | 84 | H | 88 |  | HA | 56 | HA | 57 |
| H | 68 | H | 60 |  | H | 67 | HA | 66 |
| H | 78 | H | 69 |  | H | 58 | HA | 57 |
| **7 short range** (*i - i + 2-3*) | | | |  | H | 7 | HA | 6 |
| H | 29 | H | 31 |  | H | 84 | H | 83 |
| H | 84 | H | 87 |  | H | 28 | HA | 27 |
| HA | 92 | HA | 95 |  | H | 68 | HA | 67 |
| H | 84 | H | 87 |  | H | 33 | HA | 32 |
| H | 96 | H | 94 |  | H | 28 | HA | 27 |
| H | 92 | H | 90 |  | H | 26 | HA | 25 |
| H | 87 | H | 84 |  |  |  |  |  |

Table S2: List of side-chain NOEs (59 NOEs including 50 long-range NOEs: *i* - *i* + (10-100))

| **Atom 1** | **Residue 1** | **Atom 2** | **Residue 2** |  | **Atom 1** | **Residue 1** | **Atom 2** | **Residue 2** |
| --- | --- | --- | --- | --- | --- | --- | --- | --- |
| **26 very long-range NOE** *(i - i + 25-100)* | | | |  | **24 long-range NOEs** (*i - i + 10-24*) | | | |
| HA | 50 | HG3 | 80 |  | 1HB | 35 | 1HD1 | 46 |
| 1HG1 | 92 | HA | 57 |  | 1HD1 | 35 | HB2 | 46 |
| 1HG1 | 92 | HB3 | 57 |  | HB2 | 69 | HA | 80 |
| 2HD1 | 92 | 2HD1 | 57 |  | 1HD1 | 47 | 1HD1 | 35 |
| 1HD1 | 92 | 1HD1 | 57 |  | 1HD1 | 47 | 2HD1 | 35 |
| HA | 92 | HA | 57 |  | 2HD1 | 57 | 1HD2 | 69 |
| 1HD1 | 92 | HB2 | 57 |  | 1HD1 | 55 | 1HD1 | 69 |
| 2HD1 | 92 | 1HD1 | 57 |  | HB3 | 81 | HA | 66 |
| HA | 92 | 1HD1 | 57 |  | 1HD1 | 81 | 1HD1 | 66 |
| HB2 | 92 | 2HD1 | 57 |  | HG | 33 | 1HD1 | 49 |
| HA | 92 | 2HG1 | 57 |  | 1HD1 | 9 | 2HD1 | 25 |
| H | 52 | 1HG1 | 9 |  | HA | 9 | HB2 | 25 |
| 2HD1 | 32 | HB2 | 80 |  | HA | 9 | HB3 | 25 |
| 2HD1 | 32 | 1HD1 | 80 |  | 1HD1 | 32 | HB2 | 50 |
| HA | 32 | HB2 | 80 |  | 1HB | 26 | HA | 6 |
| HA | 32 | HG2 | 80 |  | HB3 | 26 | 2HG2 | 6 |
| 1HD1 | 69 | HB3 | 9 |  | 2HG1 | 6 | HB2 | 26 |
| HA | 9 | HB3 | 80 |  | 2HG1 | 6 | HB3 | 26 |
| 2HG1 | 9 | HB3 | 80 |  | HA | 6 | HB | 26 |
| 2HG1 | 9 | HB2 | 80 |  | 1HG1 | 6 | HB3 | 26 |
| 2HD1 | 9 | 1HD1 | 80 |  | 1HD1 | 32 | 1HD1 | 9 |
| 1HD1 | 80 | 1HD1 | 9 |  | 1HG1 | 3 | HA | 26 |
| 1HB | 9 | 2HG1 | 82 |  | HB2 | 50 | HA | 74 |
| 1HG1 | 82 | HB2 | 9 |  | 1HG1 | 27 | HB2 | 3 |
| 1HB | 9 | HB3 | 91 |  | **4 middle-range NOEs** (*i - i + 4-9*) | | | |
| HA | 9 | HB2 | 91 |  | 1HD1 | 27 | HB2 | 32 |
|  |  |  |  |  | 1HD1 | 32 | HB3 | 25 |
|  |  |  |  |  | HB | 32 | HA | 25 |
|  |  |  |  |  | 2HG1 | 34 | HB2 | 25 |
|  |  |  |  |  | **5 short-range NOEs** (*i - i + 2-3*) | | | |
|  |  |  |  |  | 1HG1 | 82 | HB3 | 80 |
|  |  |  |  |  | 2HD1 | 55 | HA | 52 |
|  |  |  |  |  | HA | 46 | HB3 | 49 |
|  |  |  |  |  | 1HD1 | 46 | HB3 | 49 |
|  |  |  |  |  | HB2 | 3 | 2HG2 | 6 |

Table S3: Refinement statistics of the *Tc*Akt-PH structure determined via PSVS server ^1^

| **Model** |  |
| --- | --- |
| PDB code | 8OZZ |
| BMRB accession number | 52088 |
| Total number of residues | 109 |
| **Summary of conformationally restricting experimental constraints ^a^** | |
|  | |
| **Distance constraints** | |
| Total | **117** |
| Sequential constraints (\|*i - j*\| = 1) | 39 |
| Medium range constraints (1 < \|*i - j*\| < 5) | 12 |
| Long range constraints (\|*i - j*\| ≥ 5) | 66 |
| Ambiguous constraints | 0 |
| Distance constraints per restrained residue ^b^ | 1.6 |
| Total number of restricting constraints ^b^ | 117 |
| Total number of restricting constraints per restrained residue ^b^ | 1.6 |
| Restricting long-range constraints per restrained residue ^b^ | 0.9 |
| **Residual constraint violations ^a^** |  |
| Average number of distance violations per structure |  |
| 0.1-0.2 Å | 3 |
| 0.2-0.5 Å | 6.4 |
| > 0.5 Å | 5.7 |
| Average RMS distance violation/constraint (Å) | 1.03 |
| Maximum distance violation (Å) ^c^ | 4.9 |
| **Deviations from ideal geometry** |  |
| RMS deviation for bond angles (°) | 0.4 |
| RMS deviation for bond lengths (Å) | 0.01 |
| **RMSD from average coordinates (Å)** |  |
| Backbone atoms (Å) (all residues / ordered residues) | 2.3 / 1.5 |
| Heavy atoms (Å) (all residues / ordered residues) | 2.9 / 1.0 |
| **Ramachandran statistics** |  |
| Favored regions (%) | 93.4 |
| Allowed regions (%) | 6.6 |
| Generously allowed regions (%) | 0 |
| Disallowed regions (%) | 0 |

^a^ Analyzed for residues 3 to 106

^b^ 71 residues with conformationally restricting constraints

^c^ Largest constraint violation among all 10 reported structures


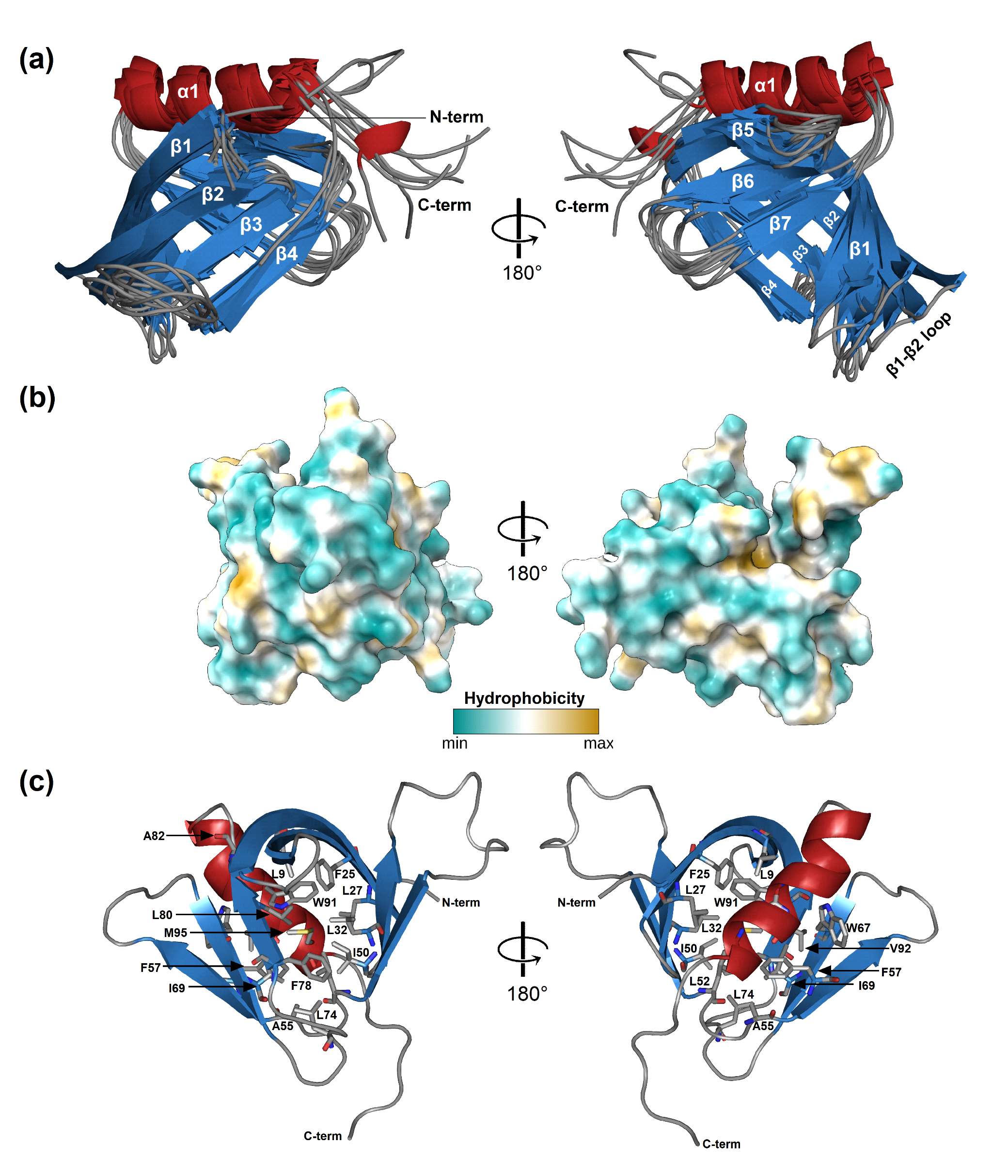


Fig. S1: Structure of *Tc*Akt-PH. (a) Bundle of 10 lowest energy structures of *Tc*Akt-PH calculated by CS-Rosetta ^2,3^: averaged Cα‑RMSD of 10 best structures compared to the lowest-energy structure is 1.7 Å. (b) Surface display of hydrophobicity: Molecular lipophilicity potential calculated in ChimeraX ^4^ with hydrophilic regions shown in turquoise and hydrophobic regions shown in gold. (c) Hydrophobic core of *Tc*Akt‑PH: Residues contributing to the hydrophobic core are shown in sticks (grey). Atom colors: nitrogen (blue), oxygen (red), hydrogen (white), sulfur (yellow).


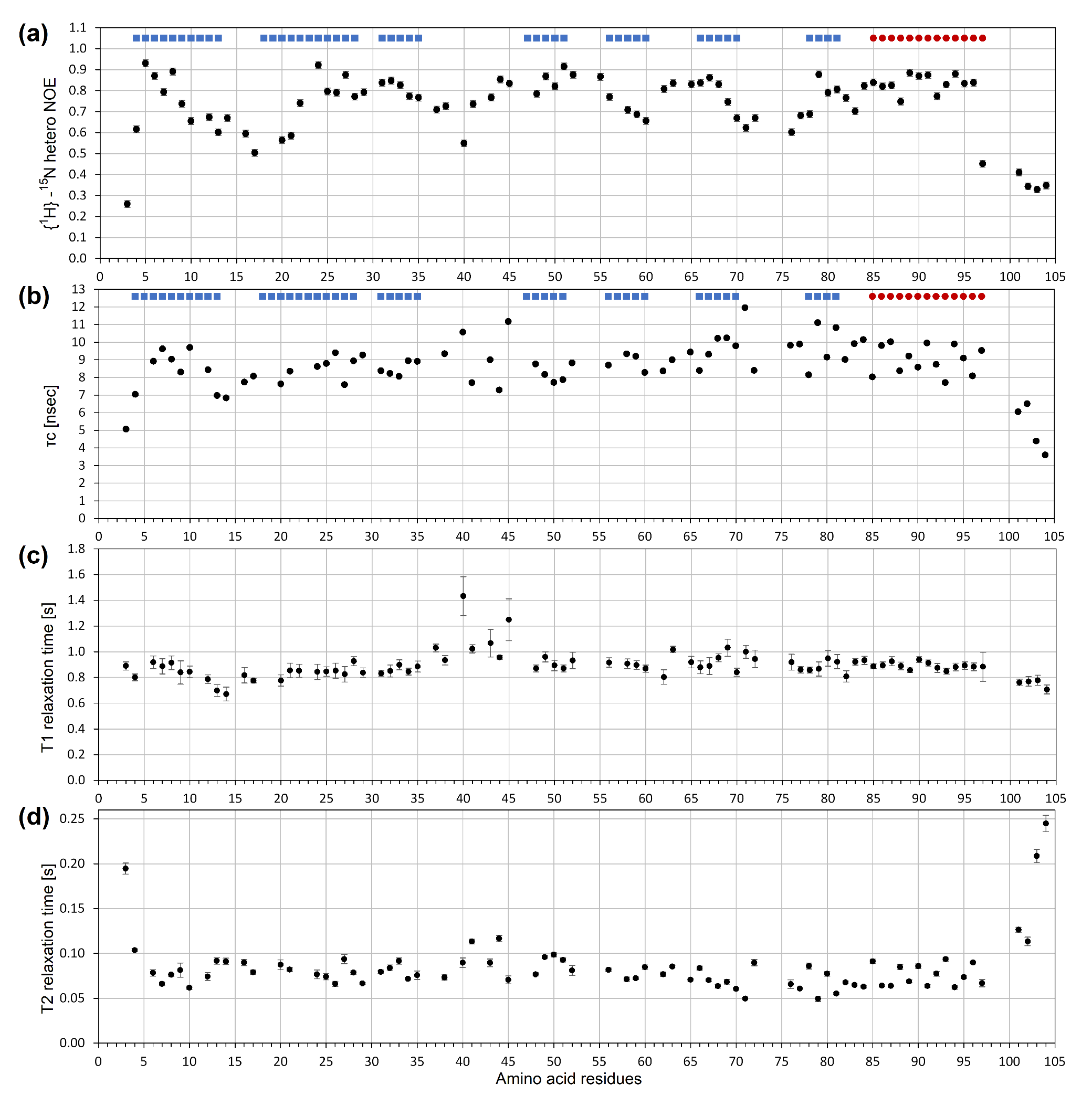


Fig. S2: NMR relaxation analysis of *Tc*Akt-PH for amino acid residues 1-105. (a) Graph of {^1^H}-^15^N heteronuclear NOE values (see equation III): Ratio from 0 to 1: Value 0 corresponds to highest flexibility, value 1 to lowest flexibility (β-strands represented as blue squares, helix represented as red spheres). (b) Rotational correlation time τ_C_ was calculated for each residue based on T1 and T2 values (see equation II) (β-strands represented as blue squares, helix represented as red spheres) and mean value was calculated: 8.68 ± 1.39 ns). (c) T1 relaxation time for each residue. (d) T2 relaxation time for each residue. HetNOE, T1 and T2 values were calculated in CcpNmr.


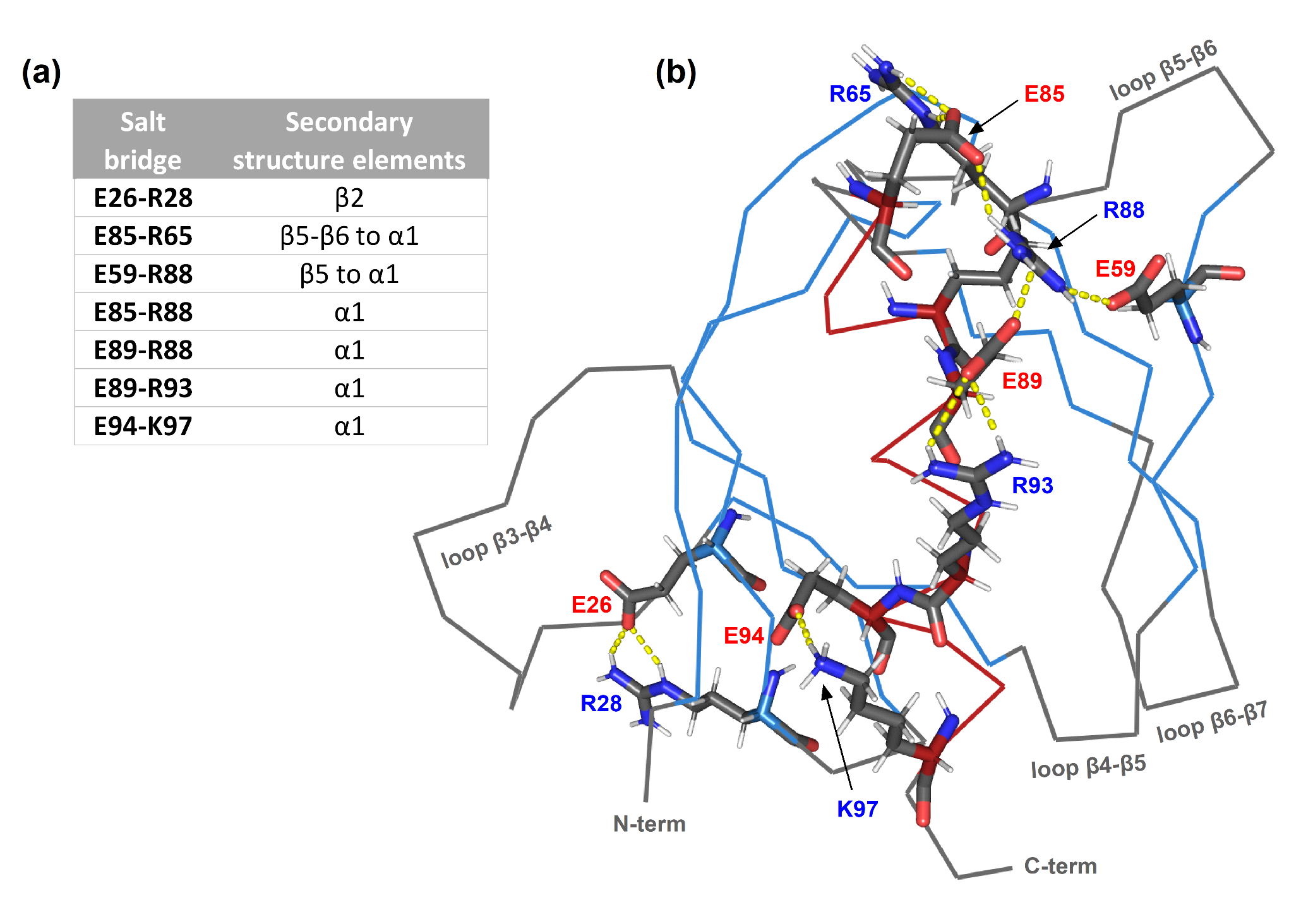


Fig. S3: Surface exposed salt bridge network of *Tc*Akt-PH. (a) Table of salt bridges with involved residues and secondary structure elements. (b) *Tc*Akt-PH with secondary structure shown in ribbon (helix in red, β-strands in blue, loops in grey): Residues involved in formation of salt bridges are shown in sticks. Salt bridges (yellow dashed lines) are formed between anionic carboxylate of glutamic acid (E) and cationic ammonium of lysine (K) or guanidinium of arginine (R). Atom colors: nitrogen (blue), oxygen (red), hydrogen (white).


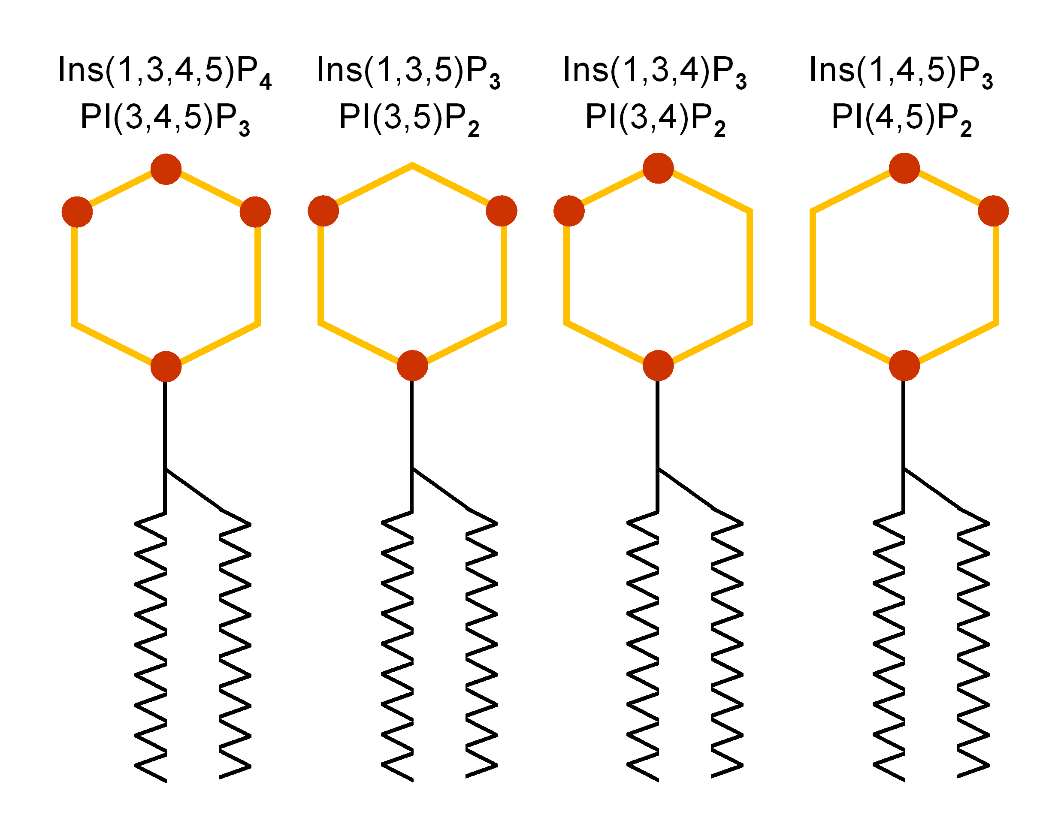


Fig. S4: Schematic overview of PIP ligands differing in the phosphorylation pattern of the inositol headgroup (yellow): Phosphate groups shown as red spheres. Fatty acid tail (black) is attached to PIPs on phosphate group P1.


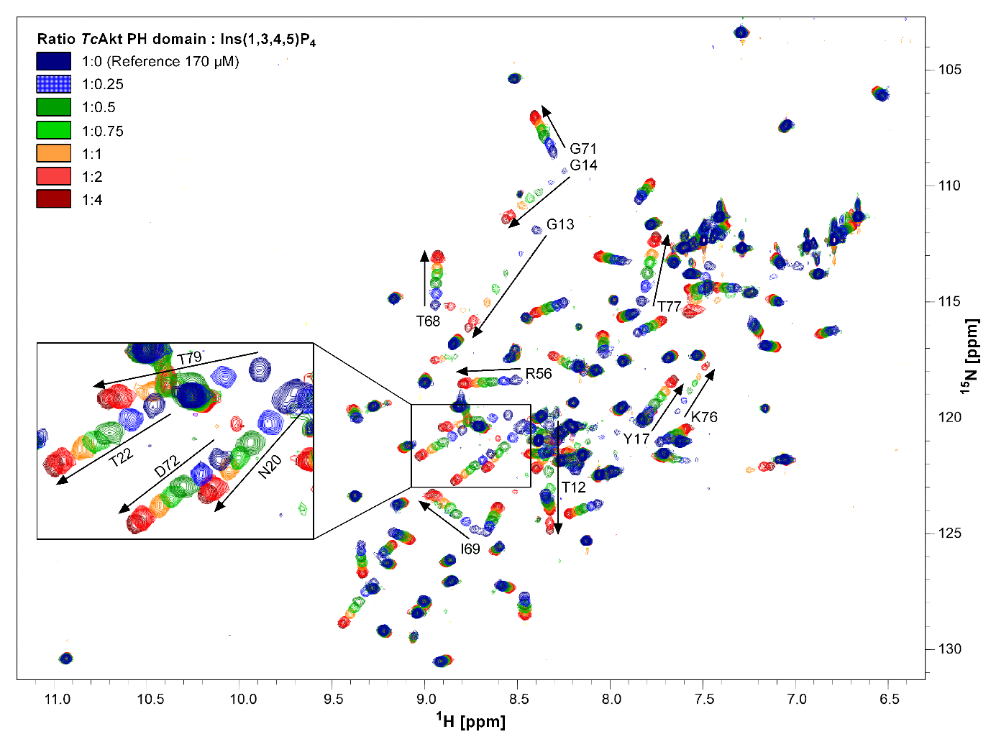


Fig. S5: Chemical shift mapping of TcAkt-PH with Ins(1,3,4,5)P4. NMR titration of TcAkt-PH and Ins(1,3,4,5)P4 revealing chemical shift changes of residues located in the binding site and residues affected by conformational changes. Strongly affected residues are highlighted. Arrows mark chemical shift perturbations upon ligand addition.

Table S4: NMR derived calculation (CcpNMR analysis output according to equation V) of dissociation constants K_d_ of *Tc*Akt-PH bound to Ins(1,3,4,5)P_4_. Residues resulting in very small *d*-values (< 0.008) and K_d_-values > 90 µM were excluded from K_d_‑calculation. An averaged K_d_ of 40 ± 14 µM was calculated defining an interaction of medium binding strength.

| Residues | K_d_ [µM] | Residues | K_d_ [µM] |
| --- | --- | --- | --- |
| 3 | 28.4204 | 55 | 55.9091 |
| 4 | 50.8756 | 56 | 23.9737 |
| 5 | 38.6537 | 58 | 28.6700 |
| 6 | 20.7637 | 59 | 53.3862 |
| 8 | 69.8903 | 60 | 26.3280 |
| 9 | 41.6556 | 63 | 35.2028 |
| 10 | 30.6152 | 65 | 59.8010 |
| 12 | 32.3848 | 66 | 30.7625 |
| 13 | 24.3201 | 67 | 30.9403 |
| 14 | 37.0535 | 68 | 25.8792 |
| 16 | 35.3303 | 69 | 26.6656 |
| 17 | 42.1927 | 70 | 36.0410 |
| 20 | 23.3281 | 71 | 31.1652 |
| 21 | 41.2371 | 72 | 78.7218 |
| 22 | 44.8170 | 76 | 27.7548 |
| 24 | 50.0521 | 77 | 28.5779 |
| 25 | 43.2265 | 78 | 44.6412 |
| 27 | 46.4175 | 79 | 29.3993 |
| 28 | 44.3731 | 80 | 26.0208 |
| 31 | 51.8746 | 81 | 33.6331 |
| 32 | 67.5143 | 82 | 73.7084 |
| 33 | 42.7062 | 83 | 60.4136 |
| 34 | 36.5712 | 84 | 37.5809 |
| 35 | 63.1563 | 87 | 30.6541 |
| 38 | 21.9287 | 88 | 53.4415 |
| 47 | 26.1787 | 90 | 30.7518 |
| 48 | 34.9618 | 91 | 53.6573 |
| 49 | 48.3543 | 92 | 52.1839 |
| 51 | 33.6849 | 94 | 58.4210 |
| 52 | 27.0115 | 95 | 44.2901 |


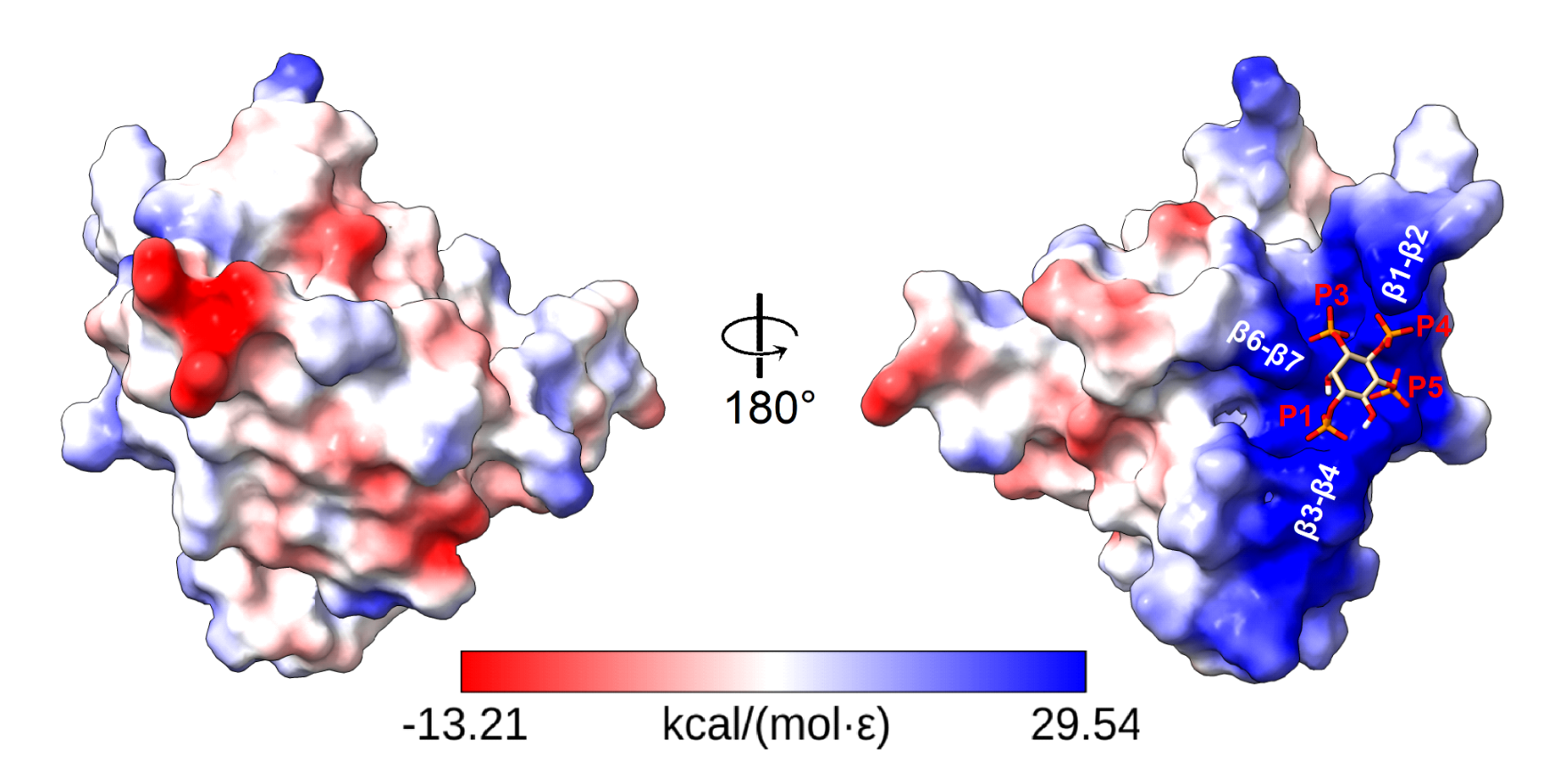


Fig. S6: Electrostatic surface of *Tc*Akt-PH bound to Ins(1,3,4,5)P_4_. Coulombic electrostatic potential in kcal/(mol.ε) at 298 K (calculated in ChimeraX ^4^). Molecular surface colors: negative electrostatic potential in red (min: -13.21), positive electrostatic potential in blue (max: 29.54). Atom colors: phosphorus (orange), oxygen (red), hydrogen (white). Loops surrounding the binding site are labelled in white.

**SI Section 1:**

**Detailed description for computed protein-ligand interactions of *Tc*Akt-PH and Ins(1,3,4,5)P_4_ shown in Fig. 3d:**

1. **Hydrogen bond (H-bond)**: A distance of 2.5 Å between the donor and acceptor atoms (D—H···A), a donor angle of ≥120 between the donor-hydrogen-acceptor atoms (D—H···A) and an acceptor angle of ≥ 90° between the hydrogen-acceptor-bonded atoms (H···A—X).
2. **Ionic or polar** **interaction**: Between two oppositely charged atoms that are within 3.7 Å distance and do not involve an H-bond.
3. **Water bridge**: H-bonded protein-ligand interactions mediated by a water molecule. The H-bond geometry is slightly relaxed from the standard H-bond definition, which is as follows: a distance of 2.8 Å between the donor and acceptor atoms (D—H···A), a donor angle of ≥ 110° between the donor-hydrogen-acceptor atoms (D—H···A) and an acceptor angle of ≥ 90° between the hydrogen-acceptor-bonded atoms (H···A—X).


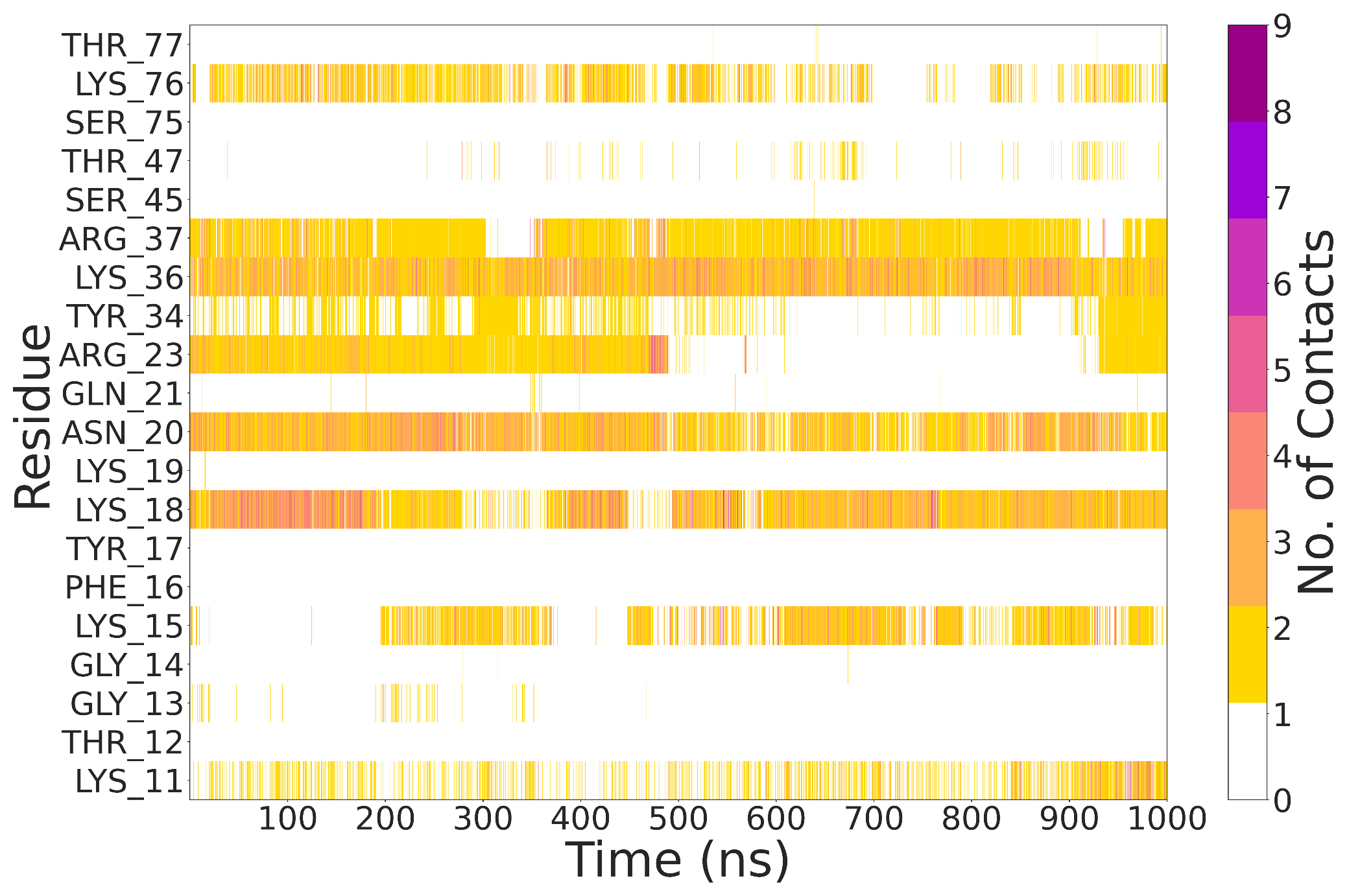


Fig. S7: Summary of Ins(1,3,4,5)P_4_ interactions with *Tc*Akt-PH (last 1000 ns MD simulation): Time dependent interactions landscape for each interacting residue. Residues revealing frequent and/or high numbers of contacts are located in the binding site and are directly involved in ligand binding.


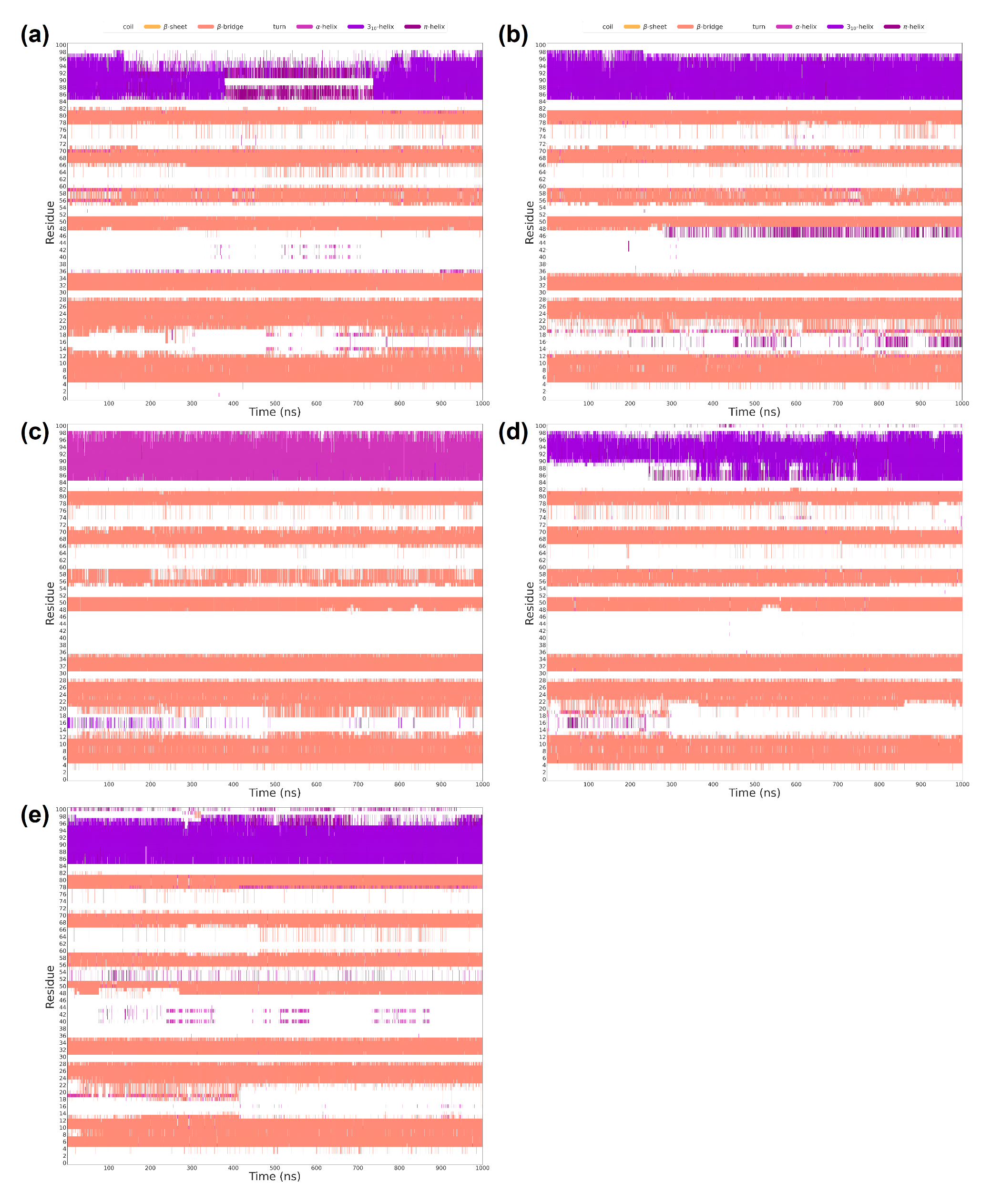


Fig. S8: Calculated secondary structure elements of *Tc*Akt-PH for each residue vs. time for the last 1000 ns MD simulation: (a) Apo-form (b) Ins(1,3,4,5)P_4_ bound (c) Ins(1,3,4)P_3_ bound (d) Ins(1,4,5)P_3_ bound (e) Ins(1,3,5)P_3_ bound. Coil and turn are shown in white, β-sheets in yellow, β-bridges in orange and α-/π-/3_10_-helices in different purple shades (see legend). The secondary structure for each residue has been calculated using the STRIDE ^5^ method implemented in VMD ^6^.


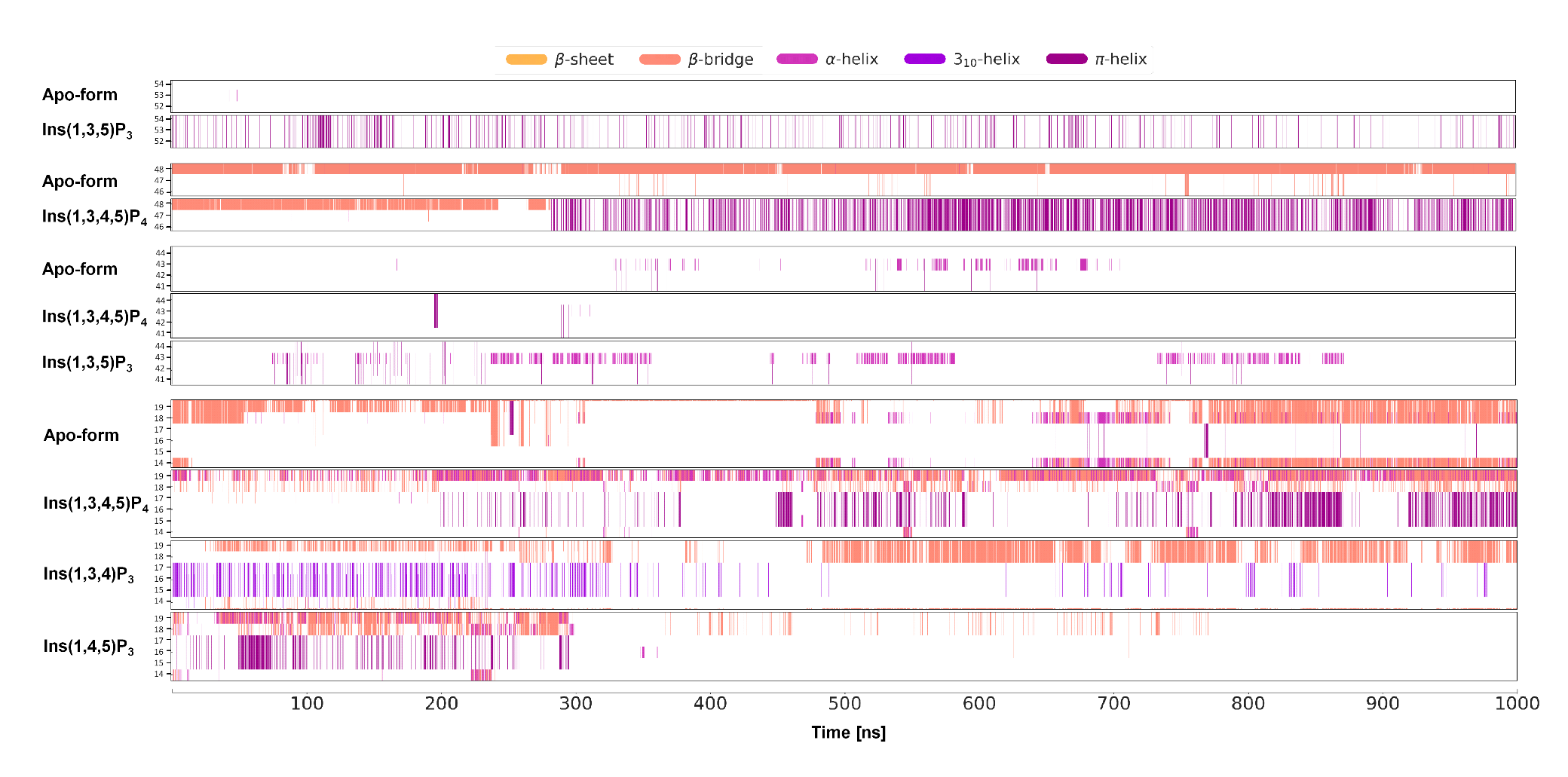


Fig. S9: Summary of calculated secondary structure elements of *Tc*Akt-PH for each residue vs. time for the last 1000 ns MD simulation: Overview over region 14-19 in loop β1-β2, region 41-44 and 46-48 in loop β3-β4 and region 52-54 in loop β4-β5. Ins(1,3,4,5)P_4_, Ins(1,3,4)P_3_, Ins(1,4,5)P_3_ and Ins(1,3,5)P_3_ bound structures are compared to the *Tc*Akt-PH apo-form, respectively. Coils and turns are shown in white, β-sheets in yellow, β-bridges in orange and α‑/π-/3_10_-helices in different purple shades. The secondary structure for each residue has been calculated using the STRIDE ^5^ method implemented in VMD ^6^.


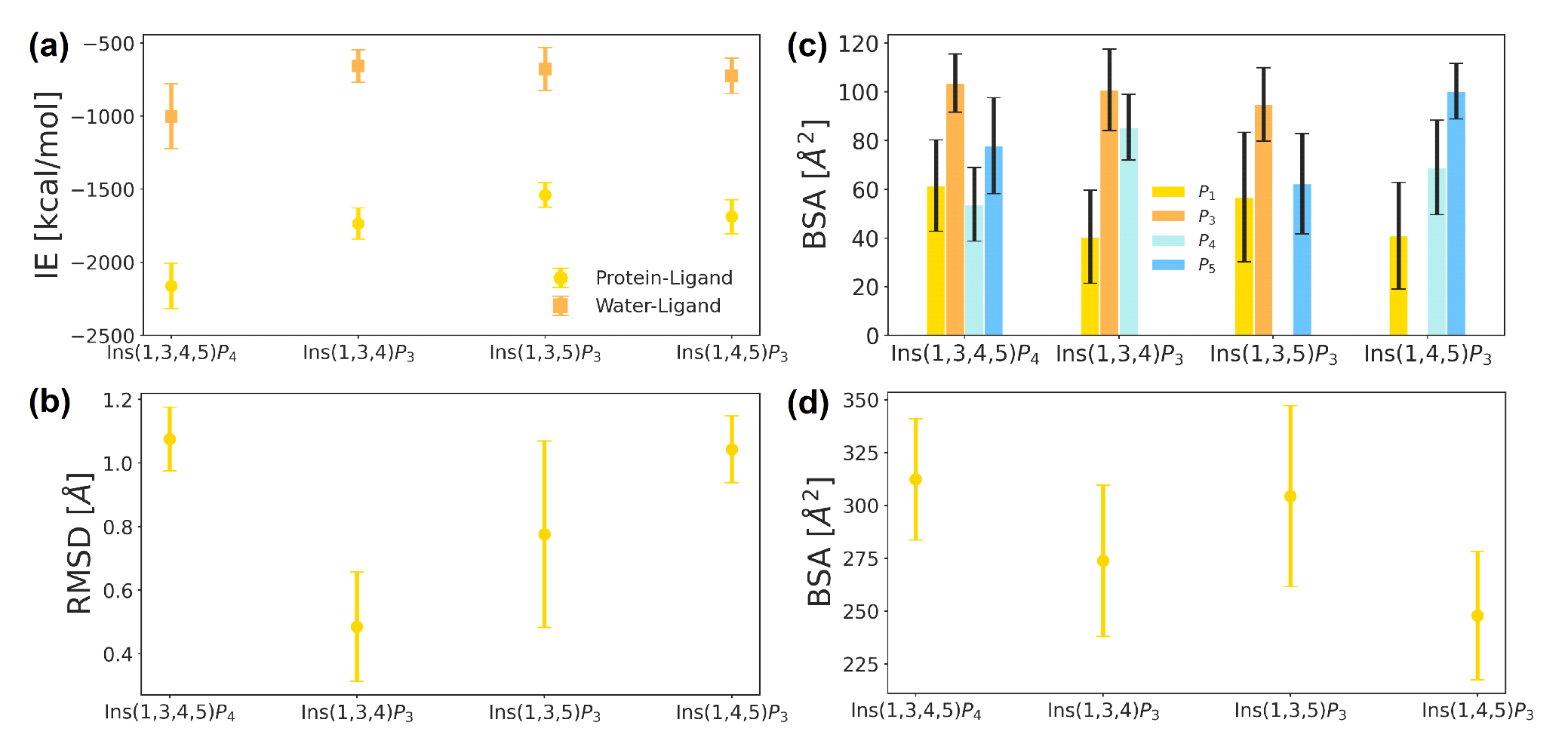


Figure S10: Comparison of systems of *Tc*Akt-PH bound to different inositol phosphates (InsP): (a) Interaction Energy (IE) in kcal/mol for protein-ligand and water-ligand (b) RMSD of InsP in binding site (c) Buried surface area (BSA) for each phosphate (PO_4_) group at different positions on inositol ring of different InsPs (d) BSA for each InsP. All figures show the analysis from the last 1000 ns MD simulation. Capped vertical lines represent standard deviation.


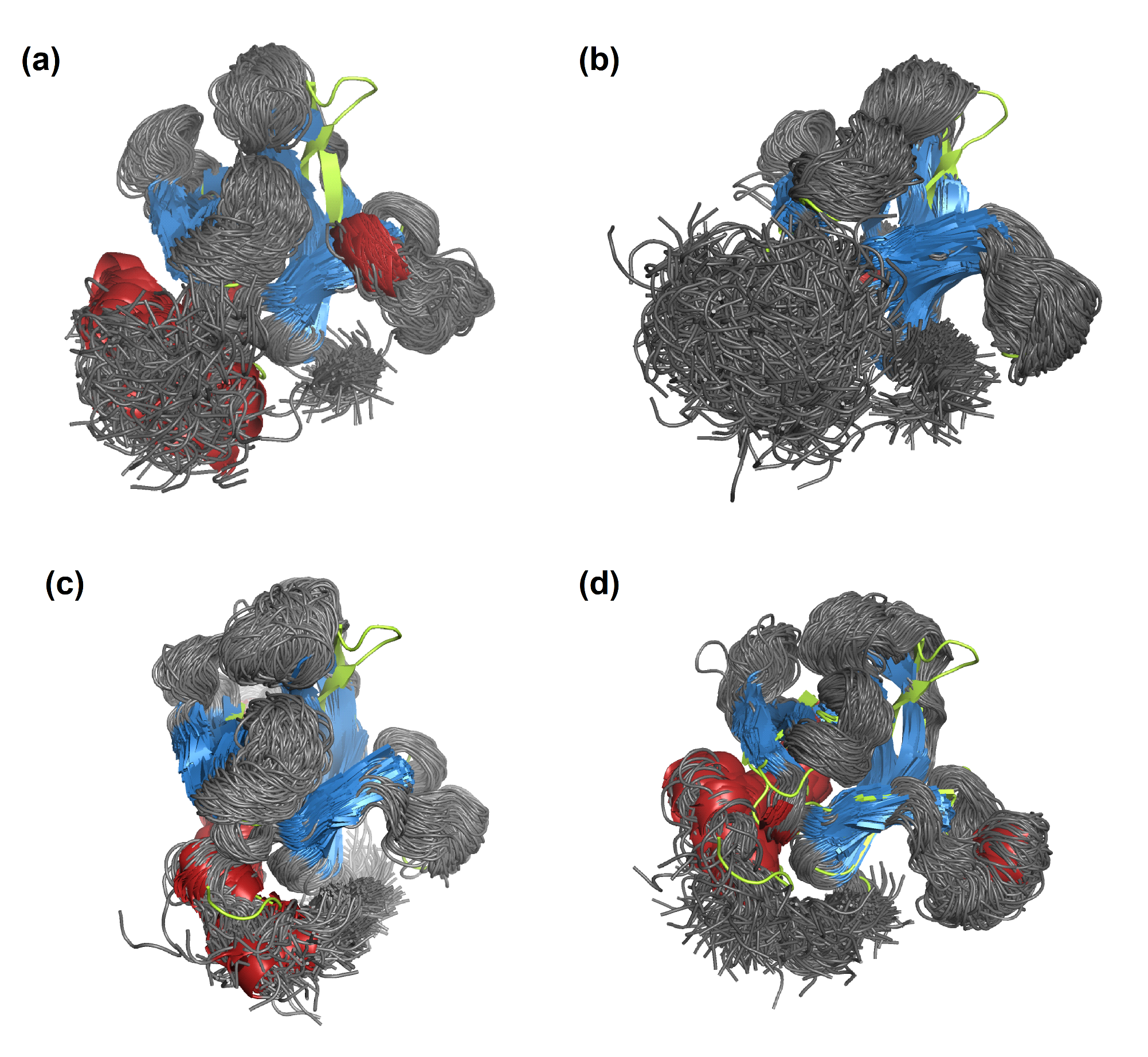


Fig. S11: Bundle of conformations from MD simulations superimposed with NMR derived *Tc*Akt-PH apo-form structure (PDB: 8OZZ) shown in green (a) Ins(1,3,4,5)P_4_ bound (b) Ins(1,3,4)P_3_ bound (c) Ins(1,4,5)P_3_ bound (d) Ins(1,3,5)P_3_ bound. Bundle of MD structures follows the same color scheme: β-strands in blue, α-helix in red, loops in gray. Loop β1-β2 of PIP-bound structures cannot bend to the same extent as loop β1-β2 of the apo-form.


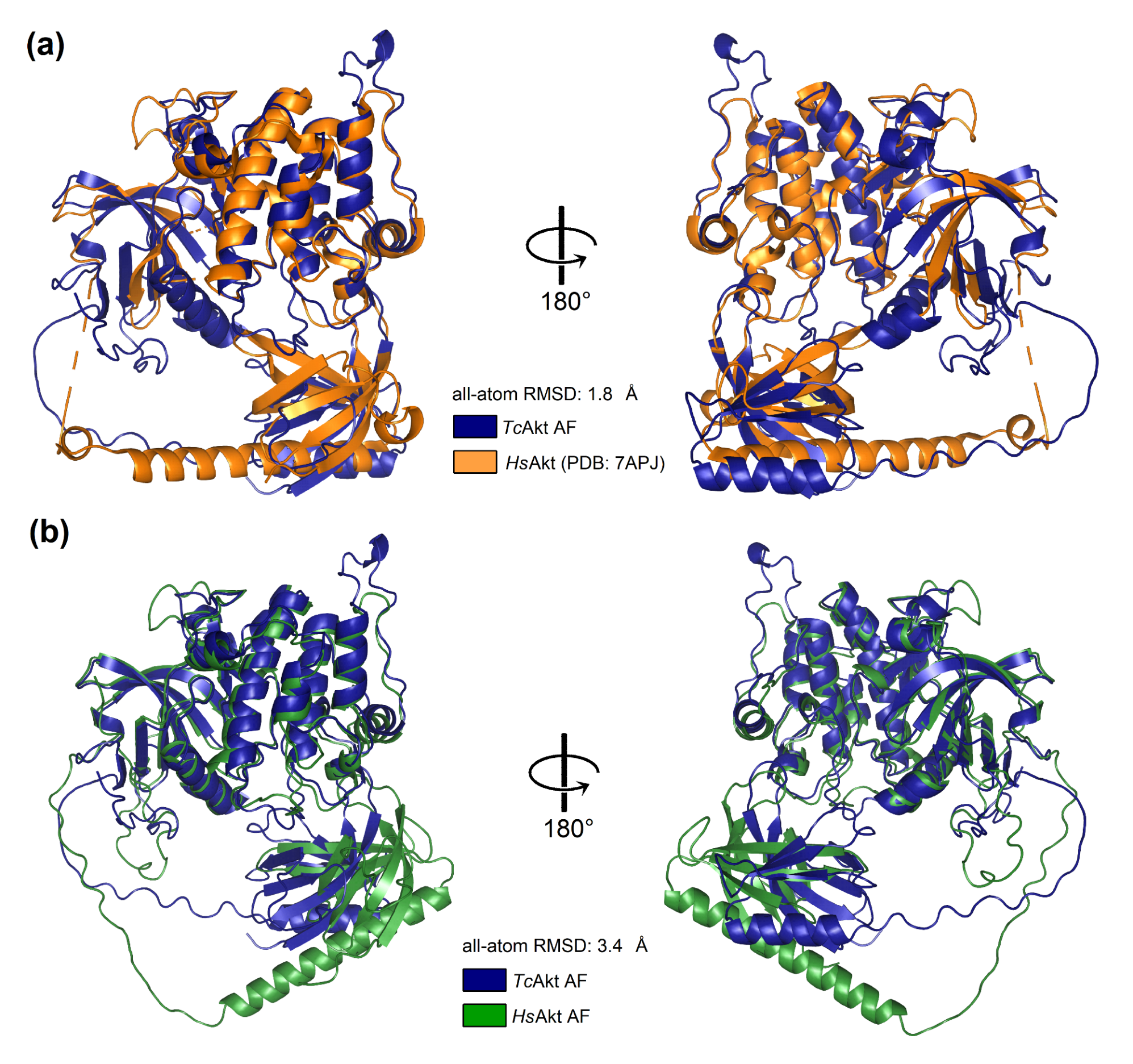


Fig. S12: Structural superimposition of AF ^7^ derived *Tc*Akt (dark blue) with human Akt: (a) Superimposition with crystal structure of human Akt1 (*Hs*Akt) (PDB: 7APJ) resulting in an all-atom RMSD of 1.8 Å. (b) Superimposition with AF structure of *Hs*Akt resulting in an all-atom RMSD of 3.4 Å.


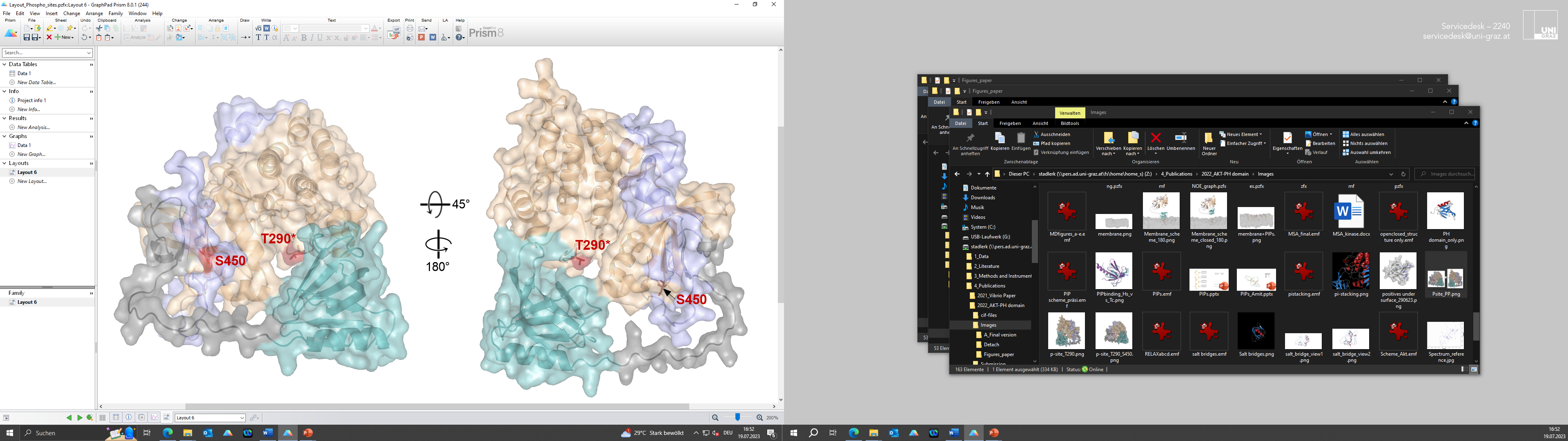


Fig. S13: *T. cruzi* putative phosphorylation sites T290 and S450 (red). T290 is located at the activation loop in the kinase domain (sand). In the closed conformation of *Tc*Akt, T290 is shielded by the PH domain (turquoise). S450 is located at the C-tail (lavender) in the hydrophobic motif and accessible also in the closed conformation. Conserved residues are marked with an asterisk.


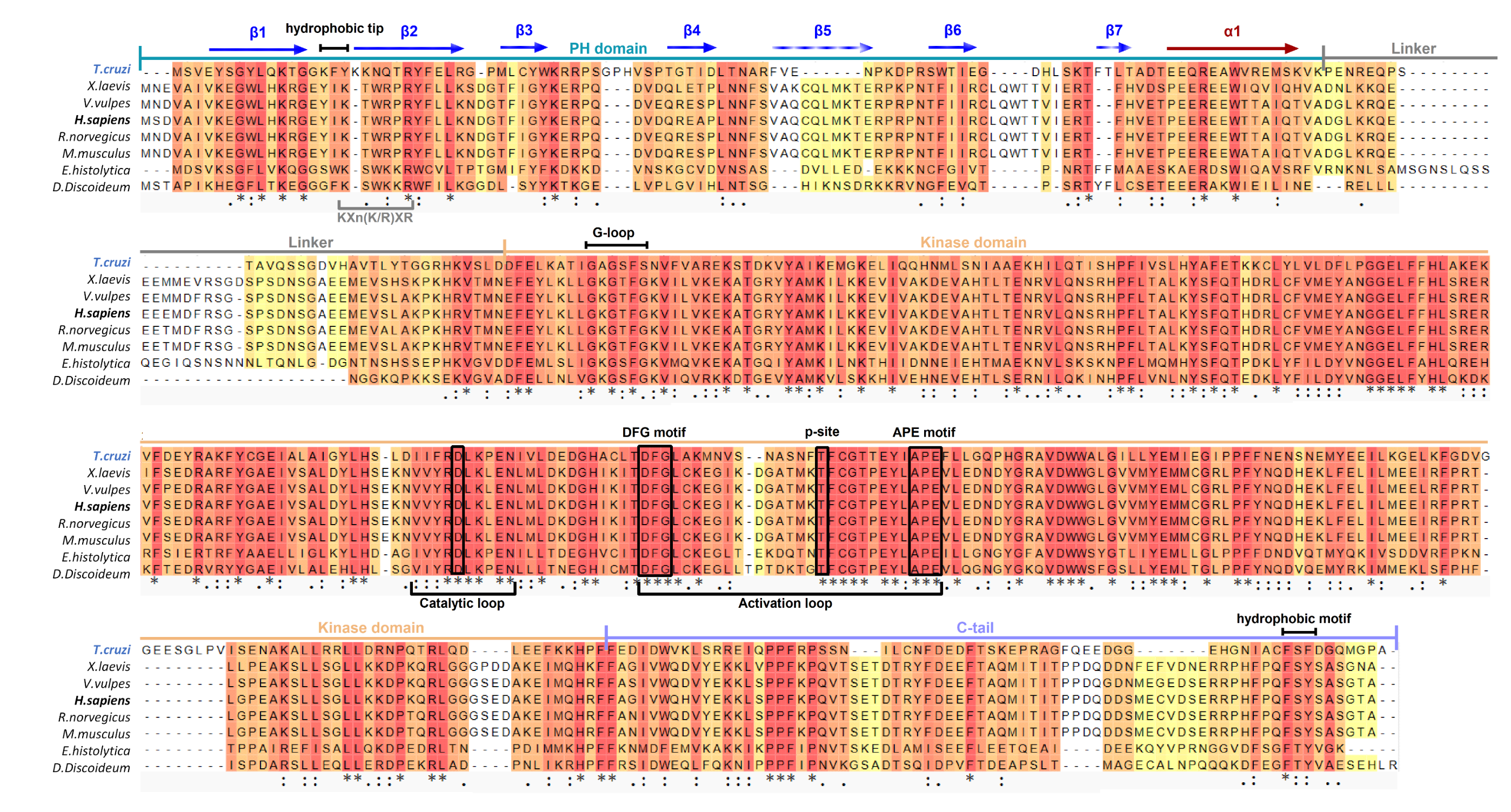


Fig. S14: Multiple sequence alignment (MSA) of Akt/Akt-*like* sequences from *T. cruzi* (Q4D6D3), frog (Akt1 *X. laevis*: Q98TY9), fox (*V. vulpes*: A0A3Q7SWZ3), human (Akt1 *H. sapiens*: P31749), rat (Akt1 *R. norvegicus*: P47196), mouse (Akt1 *M. musculus*: P31750), *E. hystolytica* (Q761W9) and *D. discoideum* (P54644): Residues are colored by conservation with a gradient from high conservation (red) to low conservation (yellow). Conserved residues are marked with an asterisk (*). Less conserved residues are marked with dots (:,.). Gaps are shown in white. Domains are marked in different colors: PH domain (turquoise), linker (grey), kinase domain (sand), C-tail (lavender). Activity essential regions are marked in black. Secondary structures of PH domain are shown with arrows: . β-strands are shown with blue arrows, α-helix is shown with red arrow. KXn(K/R)XR motif is indicated in grey.

**SI Section 2:**

**Full-length *Tc*Akt retains kinase activity and binds ADP/ATP in a Mn^2+^ dependent manner *in vitro***

The increase in affinity for manganese is not clear, however, other trypanosome enzymes as ATPases Cet1 from *T. cruzi* (*Tc*Cet1) and *T. brucei* have been characterized and shown a strong influence of Mn^2+^ on its activity, with 100-fold increase in affinity for this cation compare with its ortholog in *S. cerevisiae* ^8^. In *Tc*Cet1, the ATP-metal complexes (Mg^2+^/Mn^2+^) compete for the same site with a remarkable preference for the manganese complex ^8^. Additionally, it has been demonstrated that Tor1 kinase (TORC1), a PI3K/mTOR/Akt signaling pathway member, is substantially better activated by Mn^2+^ than by Mg^2+^ *in vitro*, and manganese activates mTORC1 signaling *in vivo* similarly both in yeast and in mammalian cells ^9^. The physiological relevance of Mn^2+^ in *T. cruzi* requires further research; however, it was recently reported that loss of the Golgi-localized Mn^2+^Ca^2+^/H^+^ exchanger alters normal protein glycosylation, host cell invasion, and intracellular replication; and protein glycosylation can be completely restored by Mn^2+^ but not by Mg^2+^, Ca^2+^ or Zn^2+^, revealing the important role of Mn^2+^ for host parasite interaction ^10^.


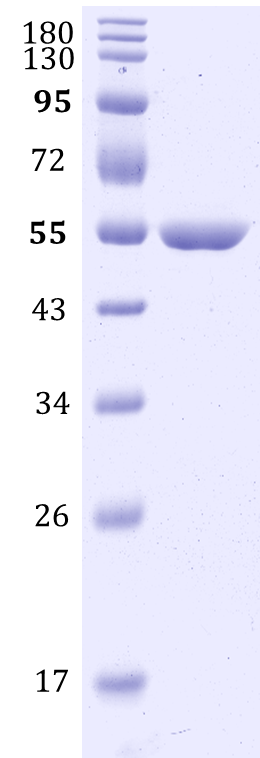


**(a)**

**(b)**

Fig. S15: Full-length *Tc*Akt SDS-PAGE and kinase activity analysis (a) SDS-PAGE of purified recombinant full-length *Tc*Akt-6His (53.2 kDa). (b) *Tc*Akt kinase activity compared to HsAkt3 activity: synthetic peptide substrate RPRAATF. TcAkt was promptly assayed post-purification; whereas HsAkt-3, acquired in the commercial kit, underwent thawing before assay, a procedure susceptible to diminishing its kinase activity. *n* = 3, error bars: mean ± SD. Statistical analysis was performed using the unpaired t-test with Welch's correction, ∗ p < 0.05 (GraphPad Prism version 8.0.1).

**(c)**

**(d)**

**(a)**

**(b)**

Fig. S16: Evaluation of effect of divalent cations (Mg^2+^, Mn^2+^ or both) and different nucleotides (ADP, ATP, ATPɤS, and AMP-PnP) on *Tc*Akt thermal stability was tested by differential scanning fluorimetry (DSF). The minimum of the derivative function of fluorescence intensity corresponds to a mid-point of the unfolding curve of the protein (Tm). Full-length *Tc*Akt: (a) in the absence of divalent cations, (b) in the presence of Mg^2+^, (c) Mn^2+^, or (d) both, Mg^2+^ and Mn^2+^. The interaction was evaluated with adenosine tri-/diphosphate ATP (blue) and ADP (violet), adenylyl imido-diphosphate AMP-PnP (green), or adenosine 5′-(ɤ-thio)triphosphate ATPɤS (brown).

Fig. S17: Histogram of ΔTm values for full-length *Tc*Akt upon cation and nucleotide binding. ΔTm values were calculated by subtracting the control Tm value (buffer, no nucleotide) from the measured Tm value. Nucleotide-binding stabilizes *Tc*Akt (increase in Tm) in a divalent cation-dependent manner. Tm increased with ADP (ΔTm = 7.9 ± 0.2 °C/ Mn^2+^; ΔTm = 8.0 ± 0.2 °C/ Mn^2+^& Mg^2+^), ATP (ΔTm = 5.0 ± 0.2 °C/ Mn^2+^; ΔTm = 5.1 ± 0.2 °C/ Mn^2+^& Mg^2+^), and ATPɤS (ΔTm = 5.9 ± 0.2 °C/ Mn^2+^; ΔTm = 6.2 ± 0.2 °C/ Mn^2+^& Mg^2+^). Incubation with any nucleotides and Mg^2+^ alone do not promote thermal stabilization, considering a thermal shift in Tm > 2° C as a robust indicator of binding ^11^.

Hydrogen bonds

--------------

<----- A T O M 1 -----> <----- A T O M 2 ----->

Atom Atom Res Res Atom Atom Res Res

no. name name no. Chain no. name name no. Chain Distance

1. 216 NZ LYS 15 A <--> 4531 O PHE 291 B 2.55

2. 1173 O HIS 73 A <--> 2670 ND2 ASN 174 B 2.71

Non-bonded contacts

-------------------

<----- A T O M 1 -----> <----- A T O M 2 ----->

Atom Atom Res Res Atom Atom Res Res

no. name name no. Chain no. name name no. Chain Distance

1. 215 CE LYS 15 A <--> 4531 O PHE 291 B 3.85

2. 216 NZ LYS 15 A <--> 4528 N PHE 291 B 3.59

3. 216 NZ LYS 15 A <--> 4529 CA PHE 291 B 3.74

4. 216 NZ LYS 15 A <--> 4530 C PHE 291 B 3.50

5. 216 NZ LYS 15 A <--> 4531 O PHE 291 B 2.55

6. 216 NZ LYS 15 A <--> 4532 CB PHE 291 B 3.64

7. 233 O PHE 16 A <--> 5253 OD1 ASN 337 B 3.89

8. 234 CB PHE 16 A <--> 5299 CD2 TYR 340 B 3.63

9. 234 CB PHE 16 A <--> 5301 CE2 TYR 340 B 3.56

10. 235 CG PHE 16 A <--> 5301 CE2 TYR 340 B 3.50

11. 235 CG PHE 16 A <--> 5302 CZ TYR 340 B 3.68

12. 236 CD1 PHE 16 A <--> 5301 CE2 TYR 340 B 3.17

13. 236 CD1 PHE 16 A <--> 5302 CZ TYR 340 B 3.13

14. 236 CD1 PHE 16 A <--> 5303 OH TYR 340 B 3.08

15. 237 CD2 PHE 16 A <--> 4715 CD2 LEU 303 B 3.36

16. 238 CE1 PHE 16 A <--> 5302 CZ TYR 340 B 3.40

17. 238 CE1 PHE 16 A <--> 5303 OH TYR 340 B 2.94

18. 239 CE2 PHE 16 A <--> 4715 CD2 LEU 303 B 3.52

19. 240 CZ PHE 16 A <--> 4637 CD1 ILE 298 B 3.71

20. 240 CZ PHE 16 A <--> 5303 OH TYR 340 B 3.86

21. 604 NZ LYS 36 A <--> 4775 CG PRO 307 B 3.88

22. 773 O THR 47 A <--> 4487 OD1 ASN 288 B 3.34

23. 775 CG2 THR 47 A <--> 4474 OG SER 287 B 3.85

24. 1161 O ASP 72 A <--> 2633 O GLN 172 B 3.67

25. 1172 C HIS 73 A <--> 2670 ND2 ASN 174 B 3.59

26. 1173 O HIS 73 A <--> 2669 CG ASN 174 B 3.43

27. 1173 O HIS 73 A <--> 2671 OD1 ASN 174 B 3.32

28. 1173 O HIS 73 A <--> 2670 ND2 ASN 174 B 2.71

29. 1188 CA LEU 74 A <--> 2670 ND2 ASN 174 B 3.84

30. 1189 C LEU 74 A <--> 2670 ND2 ASN 174 B 3.55

31. 1206 N SER 75 A <--> 2670 ND2 ASN 174 B 3.15

32. 1207 CA SER 75 A <--> 2670 ND2 ASN 174 B 3.73

33. 1225 NZ LYS 76 A <--> 4497 O PHE 289 B 3.81

Number of hydrogen bonds: 2

Number of non-bonded contacts: 33

Fig. S18: Interface contacts of *Tc*Akt (PDBsum output ^12^): PH domain corresponds to chain A, Kinase domain corresponds to chain B. Contacts are divided in H-bonds and non-bonded contacts. Atom 1 is located in the PH domain, atom 2 is located in the kinase domain.


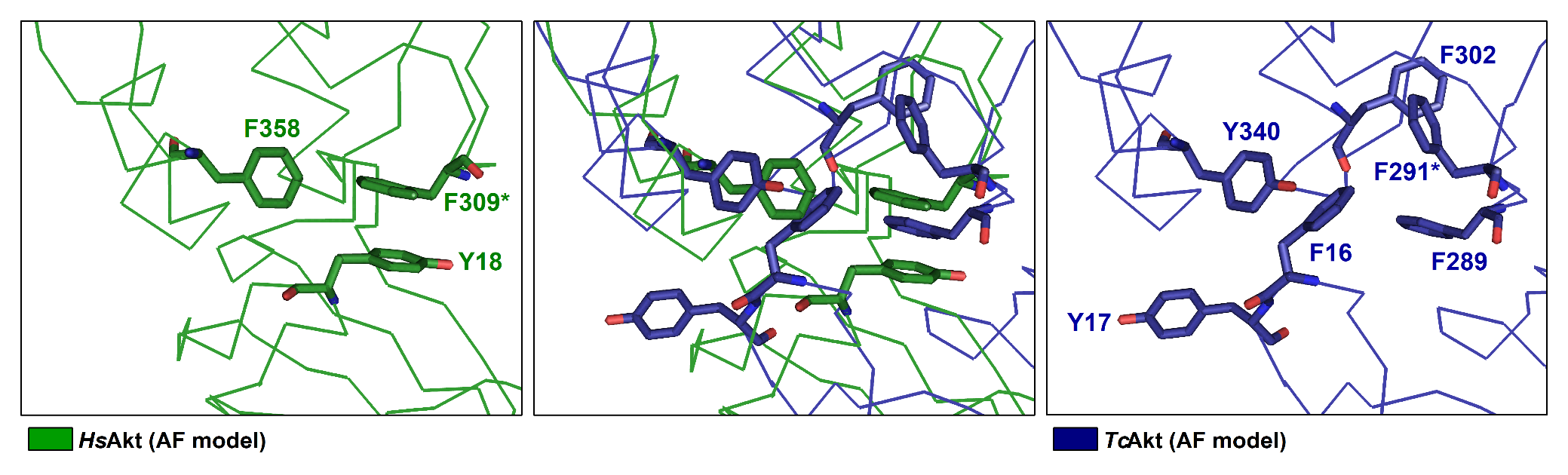


Fig. S19: Comparison of aromatic cluster in *Tc*Akt and *Hs*Akt: Aromatic cluster of human Akt1 (AF model) shown in green (left) and *Tc*Akt (AF model) shown in dark blue (right). Superimposition of both aromatic clusters (middle). Aromatic residues shown in sticks. Conserved residues are marked with an asterisk. Atom colors: nitrogen (blue), oxygen (red).


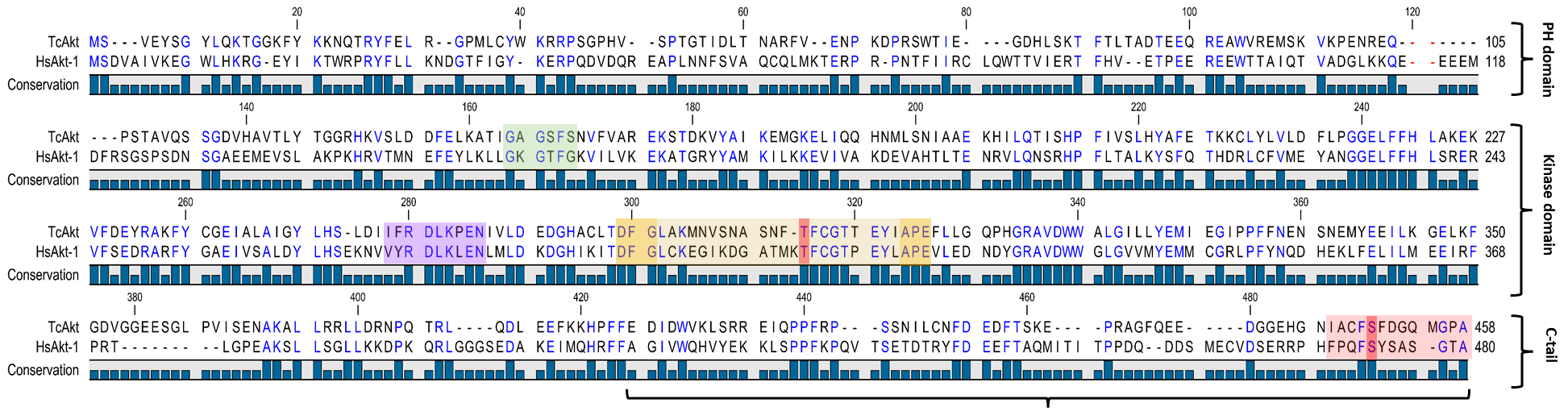
Fig. S21: Pairwise sequence alignment (PSA) of *Tc*Akt and *Hs*Akt1 (*Tc*Akt Q4D6D3), HsAkt1 P31749). PH domain, kinase domain and C-tail are indicated. Conserved residues are shown in blue font, less conserved positions in black, and insertions in red. The G-loop (GxGxΦG) is highlighted in green, DFG and APE motifs are indicated in yellow. The h-motif is highlighted in pink. P-sites are highlighted in red. The percentage of identity between the sequences is proportional to the height of the blue bars. Sequence similarity (SS) of full-length sequences is 52.6%, SS for kinase domains is 64.4% and SS for PH domains is 36.2%. Alignment was performed with CLC Genomics Workbench 23.0.5, which includes ClustalW and Muscle algorithms.

**SI Section 3:**

**Akt-*like* proteins from *Trypanosoma* and *Leishmania* reveal differences in PIP binding pocket and interdomain linker region**

We performed sequential analysis of Akt-*like* proteins from different *Trypanosoma* and *Leishmania* species (Fig. S22, S23), which are closely related protozoan parasites within the Trypanosomatidae family. *Tc*Akt shares a sequence identity range from 41 to 58 % with orthologs in other Trypanosomatid pathogens like *T. brucei* (57.64%), *L. major* (41.03%), *L. braziliensis* (41.26%) and *L. infantum* (41.03%). Conserved residues are highlighted in the full‑length structure of *Tc*Akt (Fig. 10).

The overall structure of *Trypanosoma* spp. and *Leishmania* spp. differs especially in the length of flexible regions. The linker sequence between the PH domain and the kinase domain is significantly shorter in *Leishmania* spp. (Fig. S23) possibly resulting in a different intramolecular interface. Moreover, the C-tail is longer in *Leishmania* spp. compared to *Trypanosoma* spp. which was also described by Varela-M et al. ^13^.

Crucial regions in the kinase domain tend to be conserved among *Trypanosoma* spp. and *Leishmania* spp.: G-loop (GXGXΦG), key residues in the catalytic loop, DFG and APE motif in the activation loop, threonine p-site (T290) in activation loop (Fig S23). The hydrophobic motif in the C-tail displays a similar amino acid sequence but is not conserved: YTY in *Leishmania* spp., F(S/T)F in *Trypanosoma* spp.

The PH domains show significant differences among *Trypanosoma* spp. and *Leishmania* spp.: In *Trypanosoma* spp. the hydrophobic tip (F(Y/H)) that is crucial for the interaction with the kinase domain is found in all species except for *T. brucei* (SS). In *Leishmania* spp. this motif is not found. The PIP ligand interacting residues differ among *Leishmania* spp. and *Trypanosoma* spp.: K11 and K76 are conserved among *Trypanosoma* (except *T. brucei*) (Fig. S22) but not in *Leishmania* (Fig. S23), while R23 is conserved in all mentioned strains except *L. mexicana* (Fig. S23). Basic residues in loop β3-β4 are not conserved.

The difference in residues located in the binding pocket could hint different ligand specificities and interaction modes. Additionally, the short linker of *Leishmania* spp. indicates a different position of the PH domain, thus potentially also a different mechanism of action for kinase activation.


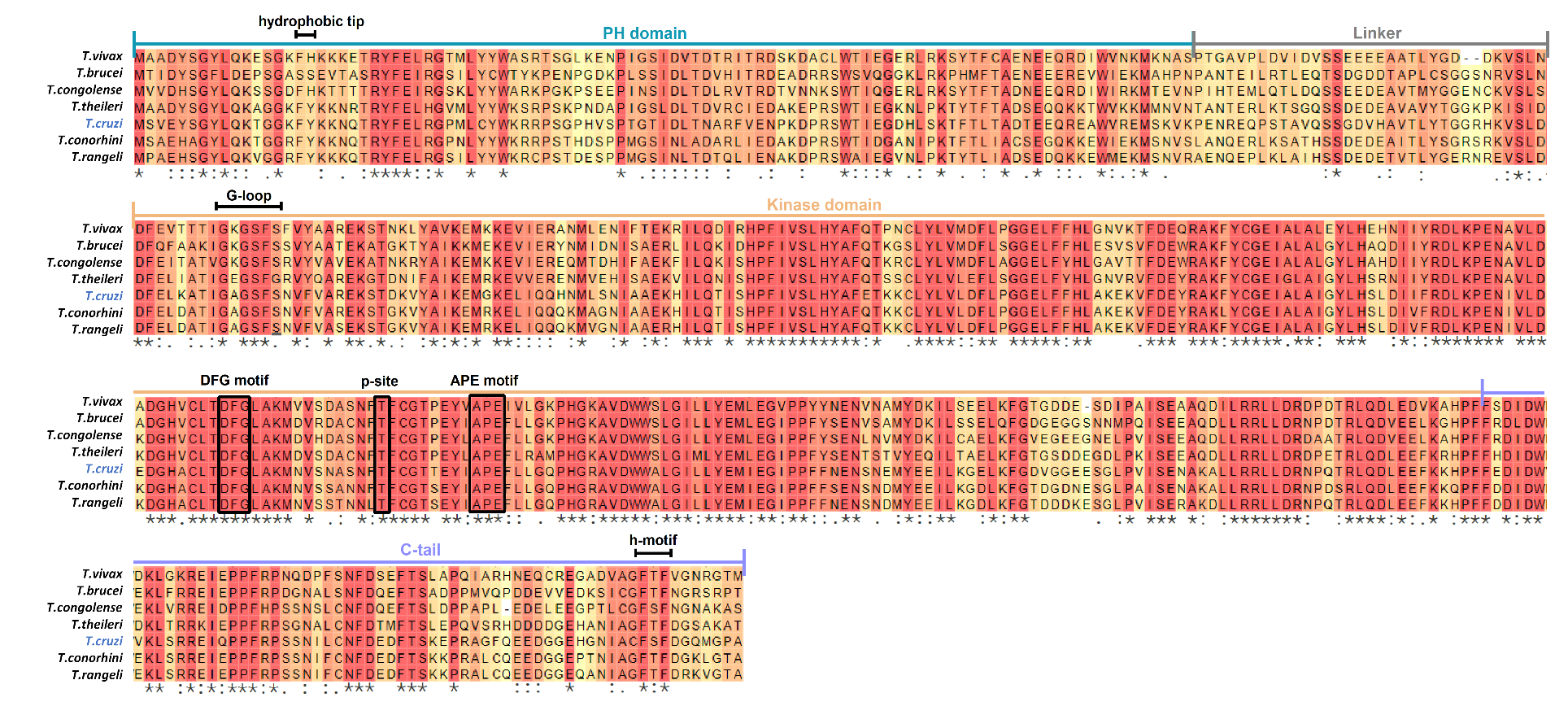


Fig. S22: MSA (Clustal O 1.2.4) of different *Trypanosoma* species: *T. cruzi* (Q4D6D3), *T. brucei* (Q584T1), *T. congolense* (F9W709), *T. conorhini* (A0A3R7L258), *T. rangeli* (A0A061J7L2), *T. theileri* (A0A1X0P3J5), *T. vivax* (G0TWP8). Residues are colored by conservation with a gradient from high conservation (red) to low conservation (yellow). Gaps are shown in white. Domains are marked in different colors: PH domain (turquoise), linker (grey), kinase domain (sand), C-tail (lavender). Activity essential regions are marked in black. Sequences were selected by BLAST search. Conserved residues are marked with an asterisk (*)). Less conserved residues are marked with dots (:,.)).


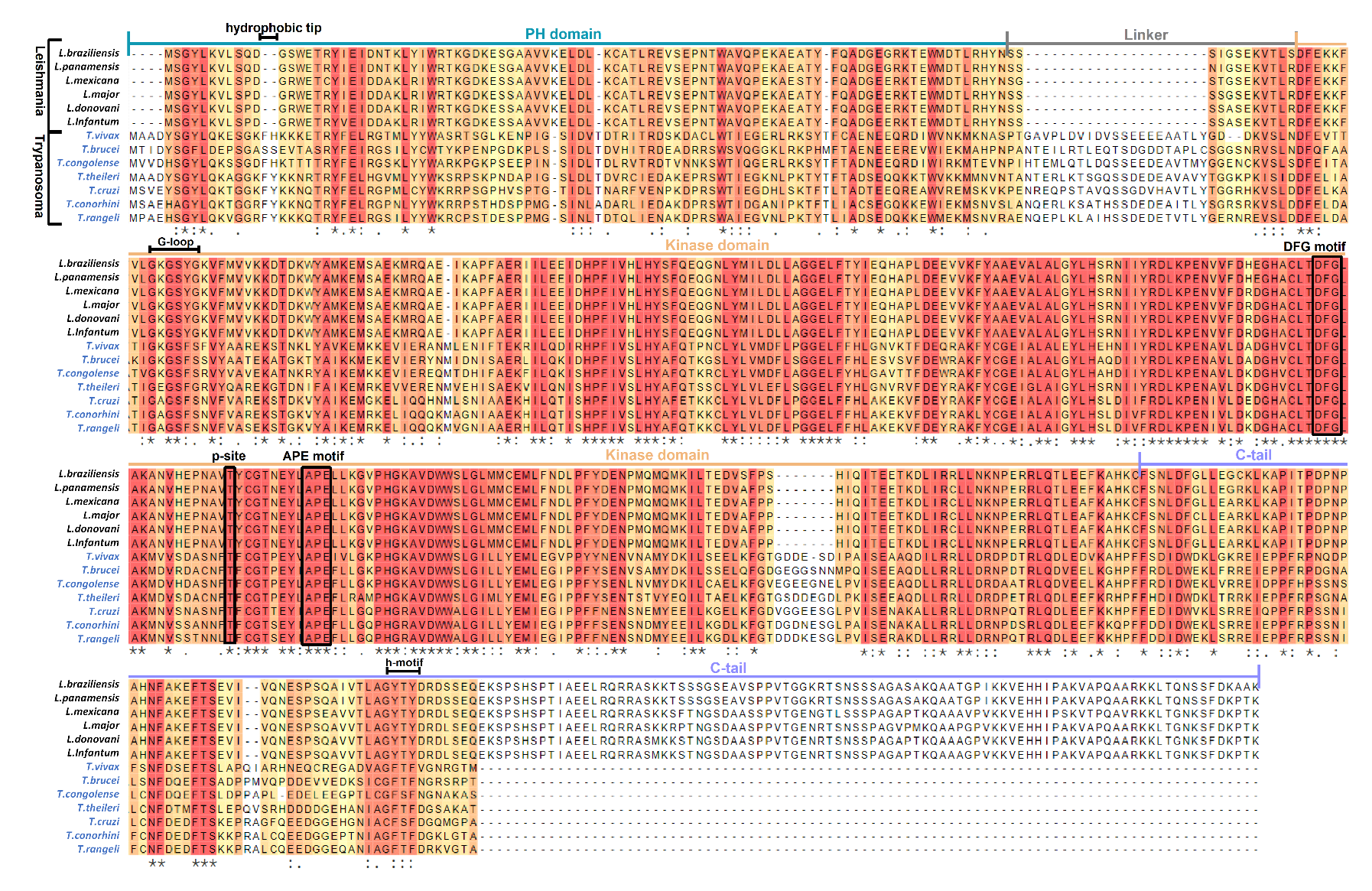


Fig. S23: MSA (Clustal O 1.2.4) of different *Trypanosoma* and *Leishmania* species: *T. cruzi* (Q4D6D3), *T. brucei* (Q584T1), *T. congolense* (F9W709), *T. conorhini* (A0A3R7L258), *T. rangeli* (A0A061J7L2), *T. theileri* (A0A1X0P3J5), *T. vivax* (G0TWP8), *L. braziliensis* (A4HI35), *L. donovani* (E9BLH8), *L. infantum* (A4I5B1), *L. major* (Q4Q7M5), *L. mexicana* (E9B0K7), *L. panamensis* (A0A0F6QP47). Residues are colored by conservation with a gradient from high conservation (red) to low conservation (yellow). Gaps are shown in white. Domains are marked in different colors: PH domain (turquoise), linker (grey), kinase domain (sand), C-tail (lavender). Activity essential regions are marked in black. Sequences were selected by BLAST search. Conserved residues are marked with an asterisk (*)). Less conserved residues are marked with dots (:,.)).

**SI Section 4:**

**Docking studies of *Tc*Akt and human Akt inhibitors capivasertib and PIT-1**

The AF model of *Tc*Akt was used for docking experiments with *Hs*Akt inhibitors capivasertib ^14^ and PIP_3_ competitor PIT-1 ^15^.

*TcAkt binds human Akt inhibitor capivasertib in a similar manner compared to HsAKt but reveals a distinct ligand stereospecifity*

According to docking experiments with AF structure of *Tc*Akt, human Akt inhibitor capivasertib has similar binding sites in *Hs*Akt and *Tc*Akt. In *Hs*Akt, E234 emerged as a critical residue with predominant interactions with inhibitors (S)-capivasertib and (R)-capivasertib, accompanied by A230 and E228 forming two hydrogen bonds with inhibitors, as depicted in Fig. 12. Analogous interactions were observed in *Tc*Akt, where E218 played a pivotal role with multiple interactions with inhibitors. At the same time, L214 and D212 established two hydrogen bonds with inhibitors, resembling the interaction pattern observed in *Hs*Akt. Interestingly, *Tc*Akt displayed a distinct affinity for (S)-capivasertib over (R)-capivasertib, whereas *Hs*Akt exhibited a less pronounced stereoisomer preference (Table S5).

Table S5: Docking of capivasertib stereoisomers to *Hs*Akt and *Tc*Akt including calculated docking scores in kcal/mol.

| Protein | 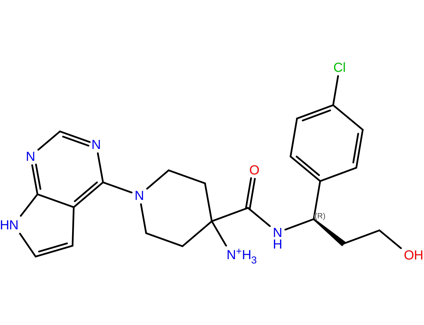(R)-Capivasertib | | 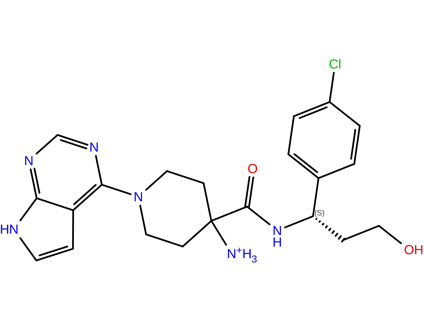(S)-Capivasertib |
| --- | --- | --- | --- |
| *Hs*Akt docking score [kcal/mol] | -9.462 | | -8.806 |
| *Tc*Akt docking score [kcal/mol] | -3.646 | | -9.945 |
|  | |  | |
|  | | | |

*Human PIP_3_ competitor recognizes basic PIP_3_ binding pocket of HsAkt and TcAkt*

Docking studies with the PH-domains of *Hs*Akt and *Tc*Akt and inhibitor PIT-1 demonstrate a similar binding preference (Table S6), with positively charged residues predominating interactions. In *Hs*Akt, K14, R23, and R86 are engaged in ionic interactions with PIT-1. In *Tc*Akt PIT-1 binding is achieved via three lysine residues at positions 11, 18, and 76 in TcAkt. Both HsAkt and TcAkt exhibited one cation‑π interaction with PIT-1 (Fig. 11). Taken together, the overall basic charge of the Akt PIP_3_ binding site enables PIT-1 binding and compensates the low sequential and structural similarity of this region.

Table S6: Results of PIT-1 docking with PH domain of respective Akt protein; the numbers shown are docking energy in kcal/mol.

| Inhibitor | docking energy to *Hs*Akt [kcal/mol] | docking energy to *Tc*Akt [kcal/mol] |
| --- | --- | --- |
| PIT-1 | -4.227 | -4.428 |

|  |  |
| --- | --- |

**M9 minimal medium**

Table S7: Components and concentrations of M9 minimal medium

| M9 minimal medium | Concentration |
| --- | --- |
| Na_2_HPO_4_  KH_2_PO_4_  pH 7.2 | 50 mM  25 mM |
| NaCl  MgSO_4_  Microsalts (from stock)  Vitamins (from stock)  ^15^NH_4_Cl  ^13^C-Glucose | 10 mM  1 mM  0.1% (v/v)  0.1% (v/v)  0.15% (w/v)  0.3% (w/v) |

Table S8: Components and concentrations of microsalts stock (1000x) and vitamins stock (1000x)

| Microsalts stock 1000x | Concentration |
| --- | --- |
| ZnCl_2_  MgCl_2_  CaCl2  H_3_BO_3_  FeCl_3_  CuCl_2_  CoCl_2_  (NH_4_)_6_Mo_7_O_24_ | 1500 mM  1000 mM  150 mM  50 mM  20 mM  800 µM  150 µM  15 µM |
| Vitamins stock 1000x | **Concentration** |
| Biotin  Thiamine | 1 mg/mL  1 mg/mL |

**Cloning**

*E. coli* codon-optimized nucleotide sequence *Tc*Akt-PH:

ATGAGTGTTGAATACAGCGGTTATCTGCAGAAAACCGGTGGCAAATTTTATAAAAAGAATCAGACCCGTTACTTCGAACTGCGTGGTCCGATGCTGTGCTATTGGAAACGCCGTCCGAGCGGTCCGCATGTTAGCCCGACCGGCACCATTGATCTGACCAATGCCCGCTTTGTGGAAAATCCGAAAGATCCGCGTAGTTGGACCATTGAAGGTGACCATCTGAGTAAAACCTTTACCCTGACCGCAGATACCGAAGAACAGCGCGAAGCATGGGTTCGTGAAATGAGTAAAGTGAAACCGGAAAATCGTGAACAG

The nucleotide sequence was inserted into a standard vector pET-28a(+) with C-terminal 6xHis-tag:

Amino acid sequence of *Tc*Akt-PH (UniProtKB entry Q4D6D3_TRYCC):

MGSVEYSGYLQKTGGKFYKKNQTRYFELRGPMLCYWKRRPSGPHVSPTGTIDLTNARFVENPKDPRSWTIEGDHLSKTFTLTADTEEQREAWVREMSKVKPENREQLEHHHHHH

Nucleotide sequence full-length *Tc*Akt (1374 bp):

ATGAGTGTGGAATATAGCGGTTATCTGCAGAAAACCGGCGGCAAATTTTATAAAAAGAATCAGACCCGTTACTTCGAACTGCGCGGTCCGATGCTGTGTTATTGGAAACGTCGTCCGAGCGGCCCGCATGTTAGTCCGACCGGTACCATTGATCTGACCAATGCCCGTTTTGTTGAAAATCCGAAAGATCCGCGTAGCTGGACCATTGAAGGTGACCATCTGAGTAAAACCTTTACCCTGACCGCAGATACCGAAGAACAGCGCGAAGCCTGGGTTCGCGAAATGAGCAAAGTGAAACCGGAAAATCGTGAACAGCCGAGCACCGCCGTGCAGAGCAGCGGTGACGTGCATGCAGTTACCCTGTATACCGGTGGCCGCCATAAAGTTAGTCTGGATGATTTTGAACTGAAAGCAACCATTGGCGCAGGTAGCTTTAGCAATGTTTTTGTGGCCCGTGAAAAAAGCACCGATAAAGTTTATGCAATTAAGGAAATGGGCAAAGAACTGATTCAGCAGCATAATATGCTGAGCAATATTGCCGCAGAAAAACATATTCTGCAGACCATTAGCCATCCGTTTATTGTGAGTCTGCATTATGCATTTGAAACCAAAAAATGCCTGTATCTGGTGCTGGATTTTCTGCCGGGCGGCGAACTGTTTTTCCATCTGGCCAAAGAAAAAGTGTTTGATGAATATCGTGCAAAATTCTATTGCGGCGAAATTGCACTGGCCATTGGTTATCTGCATAGCCTGGATATTATTTTTCGTGATCTGAAACCGGAAAACATTGTTCTGGATGAAGATGGCCATGCATGTCTGACCGATTTTGGTCTGGCAAAAATGAATGTTAGCAATGCAAGCAATTTCACCTTTTGCGGCACCACCGAATATATTGCACCGGAATTTCTGCTGGGCCAGCCGCATGGCCGTGCAGTGGATTGGTGGGCACTGGGCATTCTGCTGTATGAAATGATTGAAGGTATTCCGCCGTTTTTCAATGAAAATAGTAATGAAATGTACGAGGAGATTCTGAAAGGTGAACTGAAATTTGGTGACGTGGGTGGTGAAGAAAGTGGTCTGCCGGTTATTAGTGAAAATGCAAAAGCACTGCTGCGCCGTCTGCTGGATCGCAATCCGCAGACCCGCCTGCAGGATCTGGAAGAATTCAAAAAACATCCGTTTTTCGAAGATATCGATTGGGTGAAACTGAGTCGCCGCGAAATTCAGCCGCCGTTTCGTCCGAGTAGCAATATTCTGTGTAATTTTGATGAGGATTTCACCAGTAAAGAACCGCGTGCCGGTTTTCAGGAAGAAGATGGTGGCGAACATGGCAATATTGCCTGTTTTAGCTTTGATGGCCAGATGGGTCCGGCA

The nucleotide sequence was inserted into a standard vector pET-28a(+) with C-terminal 6xHis-tag:

MGSVEYSGYLQKTGGKFYKKNQTRYFELRGPMLCYWKRRPSGPHVSPTGTIDLTNARFVENPKDPRSWTIEGDHLSKTFTLTADTEEQREAWVREMSKVKPENREQPSTAVQSSGDVHAVTLYTGGRHKVSLDDFELKATIGAGSFSNVFVAREKSTDKVYAIKEMGKELIQQHNMLSNIAAEKHILQTISHPFIVSLHYAFETKKCLYLVLDFLPGGELFFHLAKEKVFDEYRAKFYCGEIALAIGYLHSLDIIFRDLKPENIVLDEDGHACLTDFGLAKMNVSNASNFTFCGTTEYIAPEFLLGQPHGRAVDWWALGILLYEMIEGIPPFFNENSNEMYEEILKGELKFGDVGGEESGLPVISENAKALLRRLLDRNPQTRLQDLEEFKKHPFFEDIDWVKLSRREIQPPFRPSSNILCNFDEDFTSKEPRAGFQEEDGGEHGNIACFSFDGQMGPAHHHHHH

**TALOS+ predictions**


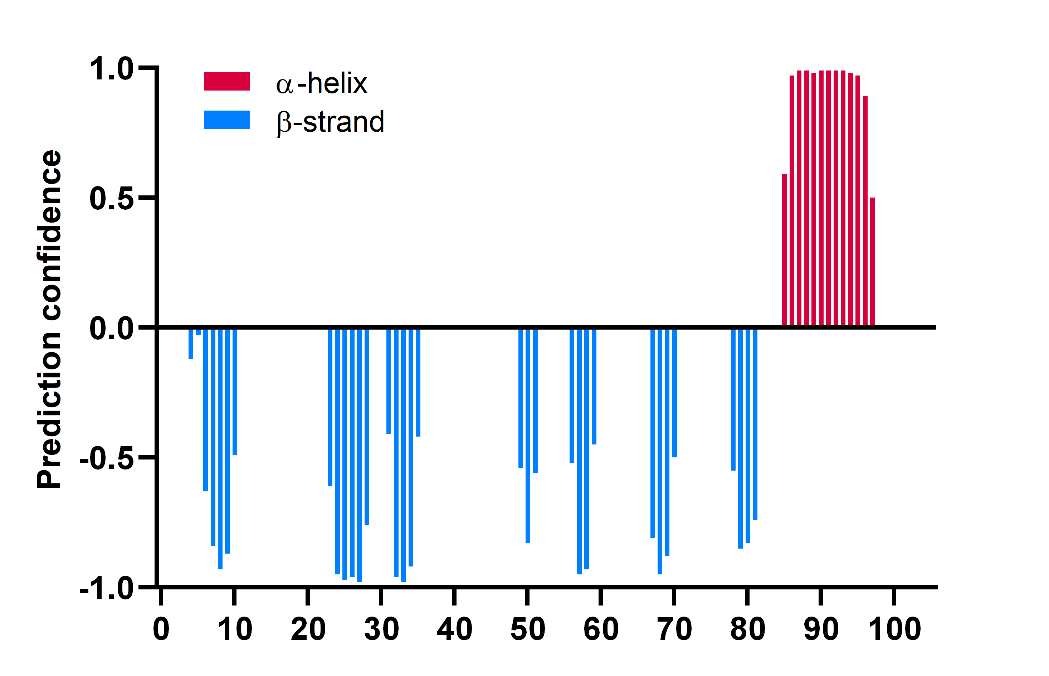


Fig. S24: Secondary structure prediction by TALOS+ ^2^. β-strands (blue) are presented in negative values (0 to ‑1), α-helix (red) is presented in positive values (0 to 1). Prediction confidence is highest at values 1/-1 and lowest at value 0. Empty areas represent loop regions or termini. Prediction confidence results from backbone torsion angles that were predicted based on NMR chemical shift data.

**References**

1. Bhattacharya, A., Tejero, R. & Montelione, G. T. Evaluating protein structures determined by structural genomics consortia. *Proteins Struct. Funct. Genet.* **66**, 778–795 (2007).

2. Shen, Y., Vernon, R., Baker, D. & Bax, A. De novo protein structure generation from incomplete chemical shift assignments. *J. Biomol. NMR* **43**, 63–78 (2009).

3. Shen, Y. *et al.* Consistent blind protein structure generation from NMR chemical shift data. *Proc. Natl. Acad. Sci. U. S. A.* **105**, 4685–4690 (2008).

4. Pettersen, E. F. *et al.* UCSF ChimeraX: Structure visualization for researchers, educators, and developers. *Protein Sci.* **30**, 70–82 (2021).

5. Frishman, D. & Argos, P. Knowledge-Based Protein Secondary Structure Assignment. *Proteins Struct. Funct. Genet.* 566–579 (1995).

6. Humphrey, W., Dalke, A. & Schulten, K. VMD: Visual Molecular Dynamics. *J. Mol. Graph.* 33–38 (1996).

7. Jumper, J. *et al.* Highly accurate protein structure prediction with AlphaFold. *Nature* **596**, 583–589 (2021).

8. Massayuki Kikuti, C., Tersariol, I. L. S. & Schenkman, S. Divalent metal requirements for catalysis and stability of the RNA triphosphatase from Trypanosoma cruzi. *Mol. Biochem. Parasitol.* **150**, 83–95 (2006).

9. Nicastro, R. *et al.* Manganese is a physiologically relevant TORC1 activator in yeast and mammals. *Elife* **11**, 1–20 (2022).

10. Ramakrishnan, S., Unger, L. M., Baptista, R. P., Cruz-Bustos, T. & Docampo, R. Deletion of a Golgi protein in Trypanosoma cruzi reveals a critical role for Mn2+ in protein glycosylation needed for host cell invasion and intracellular replication. *PLoS Pathog.* **17**, 1–27 (2021).

11. Boivin, S., Kozak, S. & Meijers, R. Optimization of protein purification and characterization using Thermofluor screens. *Protein Expr. Purif.* **91**, 192–206 (2013).

12. Laskowski, R. A. Enhancing the functional annotation of PDB structures in PDBsum using key figures extracted from the literature. *Bioinformatics* **23**, 1824–1827 (2007).

13. Varela, R. E. M., Ochoa, R., Muskus, C. E., Muro, A. & Mollinedo, F. Identification of a RAC/AKT-like gene in Leishmania parasites as a putative therapeutic target in leishmaniasis. *Parasites and Vectors* **10**, 1–10 (2017).

14. Addie, M. *et al.* Discovery of 4-amino-N-[(1S)-1-(4-chlorophenyl)-3-hydroxypropyl]-1-(7H- pyrrolo[2,3-d]pyrimidin-4-yl)piperidine-4-carboxamide (AZD5363), an orally bioavailable, potent inhibitor of Akt kinases. *J. Med. Chem.* **56**, 2059–2073 (2013).

15. Miao, R. *et al.* Akt: A Potential Drug Target for Metabolic Syndrome. *Front. Physiol.* **13**, (2022).
